# Supplementary material for: LC-DAD–ESI-MS/MS and NMR Analysis of Conifer Wood Specialized Metabolites
Source: Cells. 2022 Oct 21;11(20):3332. doi: 10.3390/cells11203332 (PMC9600761; doi:10.3390/cells11203332)
Supplement: Supplementary file 1 [file cells-11-03332-s001.zip › cells-1921806-supplementary.pdf]

Article

LC-DAD-ESI-MS/MS and NMR analysis of conifer wood specialized metabolites

Andrzej Patyra [1,2,3,*], Marta Katarzyna Dudek [4] and Anna Karolina Kiss [1,*]

[1] Department of Pharmacognosy and Molecular Basis of Phytotherapy, Medical University of Warsaw, Warsaw, Poland
[2] Doctoral School, Medical University of Warsaw, Warsaw, Poland
[3] Institut des Biomolécules Max Mousseron, Université de Montpellier, CNRS, ENSCM, Montpellier, France
[4] Structural Studies Department, Centre of Molecular and Macromolecular Studies, Polish Academy of Sciences, Łódź, Poland
* Correspondence: andrzej.patyra@wum.edu.pl (A.P.); akiss@wum.edu.pl (A.K.K.)

Supplementary Materials

Figure S1. LC-DAD chromatogram of *Abies alba* Mill. branch wood methanolic extract taken at 190-450 nm. 6

Figure S2. LC-DAD chromatogram of *Pinus sylvestris L.* branch wood methanolic extract taken at 190-450 nm. 6

Figure S3. LC-DAD chromatogram of *Pinus mugo* Turra branch wood methanolic extract taken at 190-450 nm. 7

Figure S4. LC-DAD chromatogram of *Pinus cembra* L. branch wood methanolic extract taken at 190-450 nm. 7

Figure S5. LC-DAD chromatogram of *Pinus strobus* L. branch wood methanolic extract taken at 190-450 nm. 8

Figure S6. LC-DAD chromatogram of *Pinus ×rhaetica* Brügger branch wood methanolic extract taken at 190-450 nm. 8

Figure S7. LC-DAD chromatogram of *Larix decidua* Mill. branch wood methanolic extract taken at 190-450 nm. 9

Figure S8. LC-DAD chromatogram of *Larix polonica* Rac. branch wood methanolic extract taken at 190-450 nm. 9

Figure S9. LC-DAD chromatogram of *Larix kaempferi* Lamb. branch wood methanolic extract taken at 190-450 nm. 10

Figure S10. LC-DAD chromatogram of *Pseudotsuga menziesii* Mirb. branch wood methanolic extract taken at 190-450 nm. 10

Figure S11. LC-DAD chromatogram of *Tsuga canadensis* Carrière branch wood methanolic extract taken at 190-450 nm. 11

Figure S12. LC-DAD chromatogram of *Picea abies* L. branch wood methanolic extract taken at 190-450 nm. 11

Figure S13. LC-DAD chromatogram of *Picea glauca* (Moench) Voss branch wood methanolic extract taken at 190-450 nm. 12

Figure S14. ESI-MS^2 spectrum of molecular ion at m/z 305 – compound (1). 12
Figure S15. UV spectrum of compound (1). 13
Figure S16. ESI-MS^2 spectrum (negative ion mode) of molecular ion at m/z 577 – compound (2). 13
Figure S17. UV spectrum of compound (2). 14
Figure S18. ESI-MS^2 spectrum (negative ion mode) of molecular ion at m/z 577 – compound (3). 14
Figure S19. UV spectrum of compound (3). 15
Figure S20. ESI-MS^2 spectrum (negative ion mode) of molecular ion at m/z 865 – compound (4). 15
Figure S21. UV spectrum of compound (4). 16
Figure S22. ESI-MS^2 spectrum (negative ion mode) of molecular ion at m/z 289 – compound (5). 16
Figure S23. UV spectrum of compound (5). 17
Figure S24. ESI-MS^2 spectrum (negative ion mode) of molecular ion at m/z 577 – compound (6). 17
Figure S25. UV spectrum of compound (6). 18
Figure S26. ESI-MS^2 spectrum (negative ion mode) of molecular ion at m/z 289 – compound (7). 18
Figure S27. UV spectrum of compound (7). 19
Figure S28. ESI-MS^2 spectrum (negative ion mode) of molecular ion at m/z 319 – compound (8). 19
Figure S29. UV spectrum of compound (8). 20
Figure S30. ESI-MS^2 spectrum (negative ion mode) of molecular ion at m/z 375 – compound (9). 20
Figure S31. UV spectrum of compound (9). 21
Figure S32. ESI-MS^2 spectrum (negative ion mode) of molecular ion at m/z 405 – compound (10). 21
Figure S33. UV spectrum of compound (10). 22
Figure S34. ESI-MS^2 spectrum (negative ion mode) of molecular ion at m/z 287 – compound (11). 22
Figure S35. UV spectrum of compound (11). 23
Figure S36. ESI-MS^2 spectrum (negative ion mode) of molecular ion at m/z 375 – compound (12). 23

Figure S37. UV spectrum of compound (12). 24
Figure S38. ESI-MS^2 spectrum (negative ion mode) of molecular ion at m/z 465 – compound (13). 24
Figure S39. UV spectrum of compound (13). 25
Figure S40. ESI-MS^2 spectrum (negative ion mode) of molecular ion at m/z 405 – compound (14). 25
Figure S41. UV spectrum of compound (14). 26
Figure S42. ESI-MS^2 spectrum (negative ion mode) of molecular ion at m/z 577 – compound (15). 26
Figure S43. UV spectrum of compound (15). 27
Figure S44. ESI-MS^2 spectrum (negative ion mode) of molecular ion at m/z 421 – compound (16). 27
Figure S45. UV spectrum of compound (16). 28
Figure S46. ESI-MS^2 spectrum (negative ion mode) of molecular ion at m/z 389 – compound (17). 28
Figure S47. UV spectrum of compound (17). 29
Figure S48. ESI-MS^2 spectrum (negative ion mode) of molecular ion at m/z 303 – compound (18). 29
Figure S49. UV spectrum of compound (18). 30
Figure S50. ESI-MS^2 spectrum (negative ion mode) of molecular ion at m/z 465 – compound (19). 30
Figure S51. UV spectrum of compound (19). 31
Figure S52. ESI-MS^2 spectrum (negative ion mode) of molecular ion at m/z 359 – compound (20). 31
Figure S53. UV spectrum of compound (20). 32
Figure S54. ESI-MS^2 spectrum (negative ion mode) of molecular ion at m/z 419 – compound (21). 32
Figure S55. UV spectrum of compound (21). 33
Figure S56. ESI-MS^2 spectrum (negative ion mode) of molecular ion at m/z 373 – compound (22). 33
Figure S57. UV spectrum of compound (22). 34
Figure S58. ESI-MS^2 spectrum (negative ion mode) of molecular ion at m/z 361 – compound (23). 34
Figure S59. UV spectrum of compound (23). 35

Figure S60. ESI-MS^2 spectrum (negative ion mode) of molecular ion at m/z 287 – compound (24) 35
Figure S61. UV spectrum of compound (24) 36
Figure S62. ESI-MS^2 spectrum (negative ion mode) of molecular ion at m/z 317 – compound (25) 36
Figure S63. UV spectrum of compound (25) 37
Figure S64. ESI-MS^2 spectrum (negative ion mode) of molecular ion at m/z 557 – compound (26) 37
Figure S65. UV spectrum of compound (26) 38
Figure S66. ESI-MS^2 spectrum (negative ion mode) of molecular ion at m/z 405 – compound (27) 38
Figure S67. UV spectrum of compound (27) 39
Figure S68. ESI-MS^2 spectrum (negative ion mode) of molecular ion at m/z 555 – compound (28) 39
Figure S69. UV spectrum of compound (28) 40
Figure S70. ESI-MS^2 spectrum (negative ion mode) of molecular ion at m/z 373 – compound (29) 40
Figure S71. UV spectrum of compound (29) 41
Figure S72. ESI-MS^2 spectrum (negative ion mode) of molecular ion at m/z 357 – compound (30) 41
Figure S73. UV spectrum of compound (30) 42
Figure S74. ESI-MS^2 spectrum (negative ion mode) of molecular ion at m/z 301 – compound (31) 42
Figure S75. UV spectrum of compound (31) 43
Figure S76. ESI-MS^2 spectrum (negative ion mode) of molecular ion at m/z 357 – compound (32) 43
Figure S77. UV spectrum of compound (32) 44
Figure S78. ESI-MS^2 spectrum (negative ion mode) of molecular ion at m/z 271 – compound (33) 44
Figure S79. UV spectrum of compound (33) 45
Figure S80. ESI-MS^2 spectrum (negative ion mode) of molecular ion at m/z 211 – compound (34) 45
Figure S81. UV spectrum of compound (34) 46
Figure S82. ESI-MS^2 spectrum (negative ion mode) of molecular ion at m/z 255 – compound (35) 46

Figure S83. UV spectrum of compound (35). 47
Figure S84. ESI-MS² spectrum (negative ion mode) of molecular ion at m/z 313 – compound (36). 47
Figure S85. UV spectrum of compound (36). 48
Figure S86. ESI-MS² spectrum (positive ion mode) of molecular ion at m/z 227 – compound (37). 48
Figure S87. UV spectrum of compound (37). 49
Figure S88. ESI-MS² spectrum (positive ion mode) of molecular ion at m/z 265 – compound (38). 49
Figure S89. UV spectrum of compound (38). 50
Figure S90. ESI-MS² spectrum (positive ion mode) of molecular ion at m/z 303 – compound (39). 50
Figure S91. UV spectrum of compound (39). 51
Figure S92. ESI-MS² spectrum (positive ion mode) of molecular ion at m/z 303 – compound (40). 51
Figure S93. UV spectrum of compound (40). 52
Figure S94. ¹H NMR spectrum of compound (9). 53
Figure S95. ¹H NMR spectrum of compound (12). 54
Figure S96. ¹H NMR spectrum of compound (16). 55
Figure S97. ¹H NMR spectrum of compound (20). 56
Figure S98. ¹H NMR spectrum of compound (22). 57
Figure S99. ¹H NMR spectrum of compound (23). 58
Figure S100. ¹H NMR spectrum of compound (27). 59
Figure S101. ¹H NMR spectrum of compound (29). 60
Figure S102. ¹H NMR spectrum of compound (30). 61
Figure S103. ¹H NMR spectrum of compound (32). 62
Table S1. ¹H chemical shifts in ppm for compounds 9, 12, 16, 20, 22, 23, 27, 30 and 32 in methanol-d4 and 29 in chloroform-d (500 MHz, 295 K). 63
Table S2. Canonical SMILES of identified compounds 64

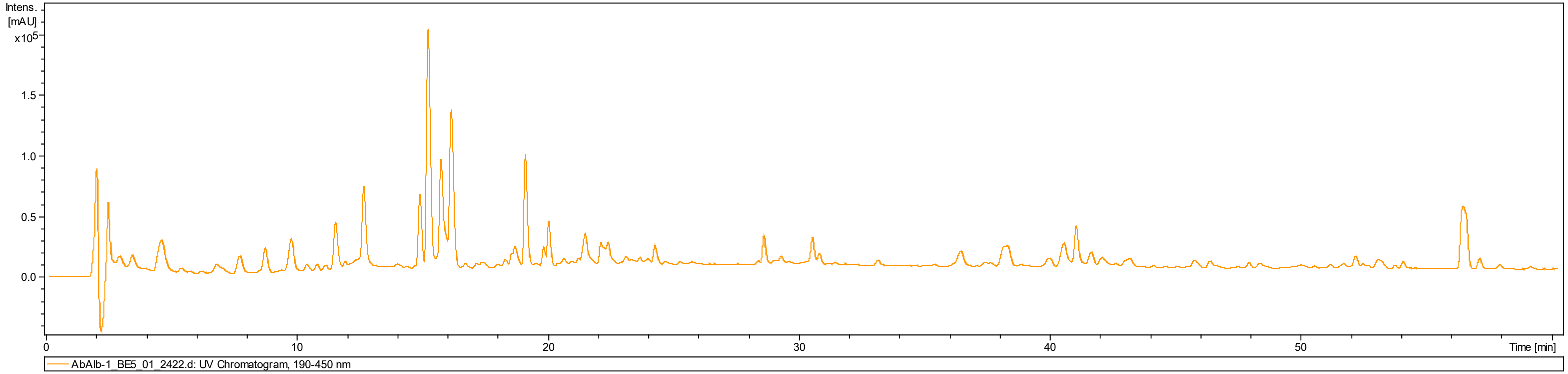

Figure S1. LC-DAD chromatogram of *Abies alba* Mill. branch wood methanolic extract taken at 190-450 nm.

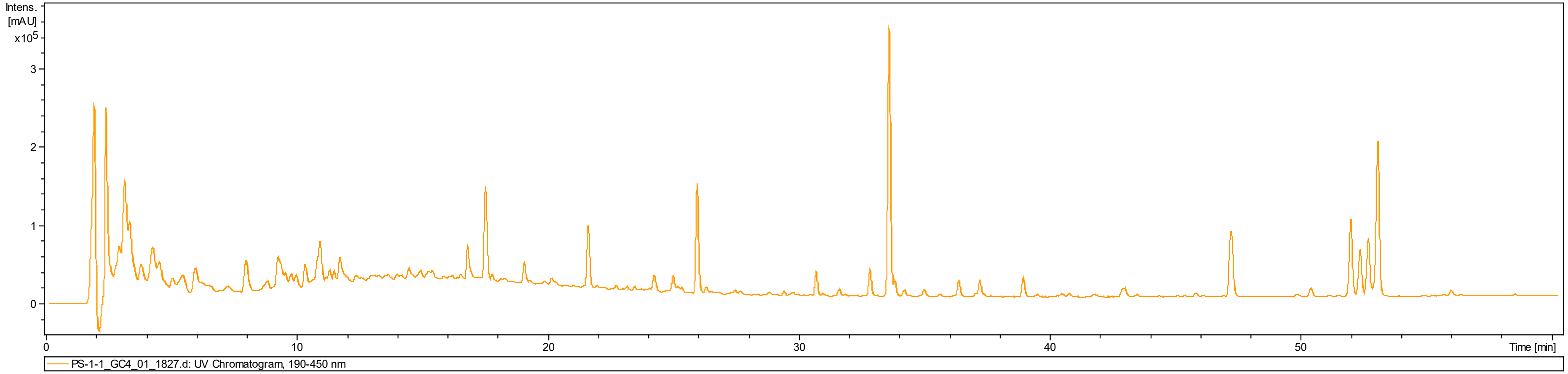

Figure S2. LC-DAD chromatogram of *Pinus sylvestris L*. branch wood methanolic extract taken at 190-450 nm.

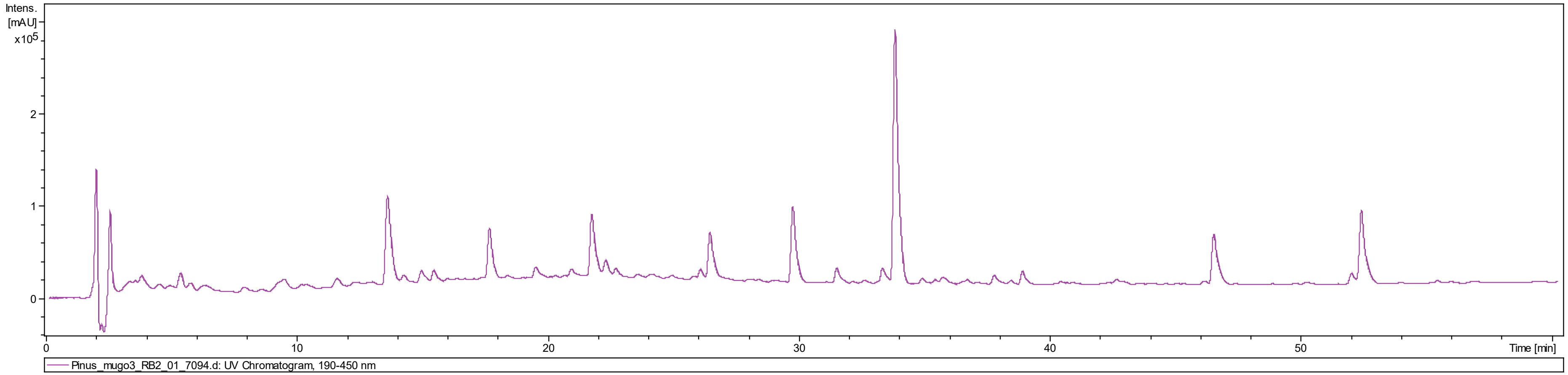

Figure S3. LC-DAD chromatogram of *Pinus mugo* Turra branch wood methanolic extract taken at 190-450 nm.

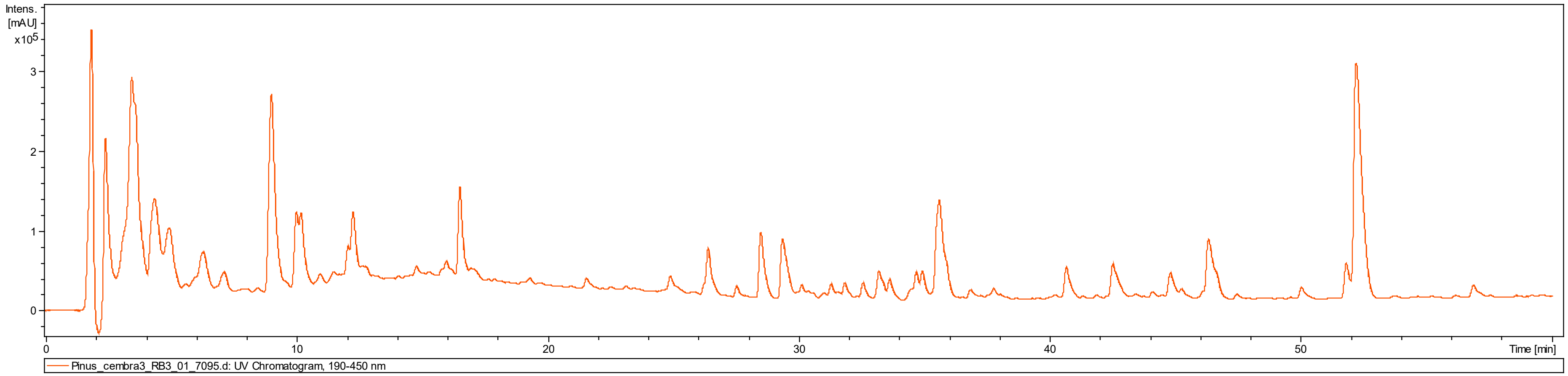

Figure S4. LC-DAD chromatogram of *Pinus cembra* L. branch wood methanolic extract taken at 190-450 nm.

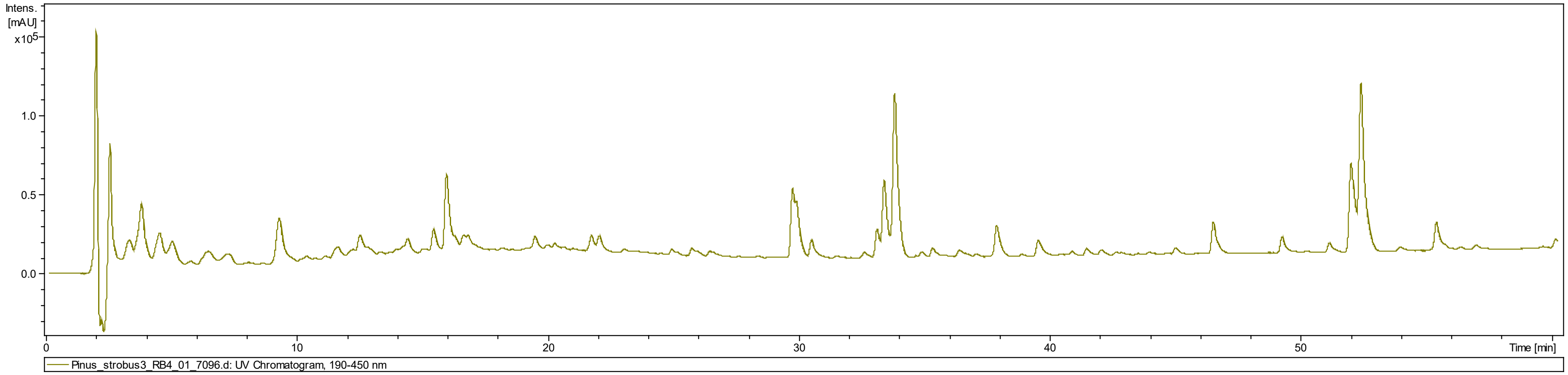

Figure S5. LC-DAD chromatogram of *Pinus strobus* L. branch wood methanolic extract taken at 190-450 nm.

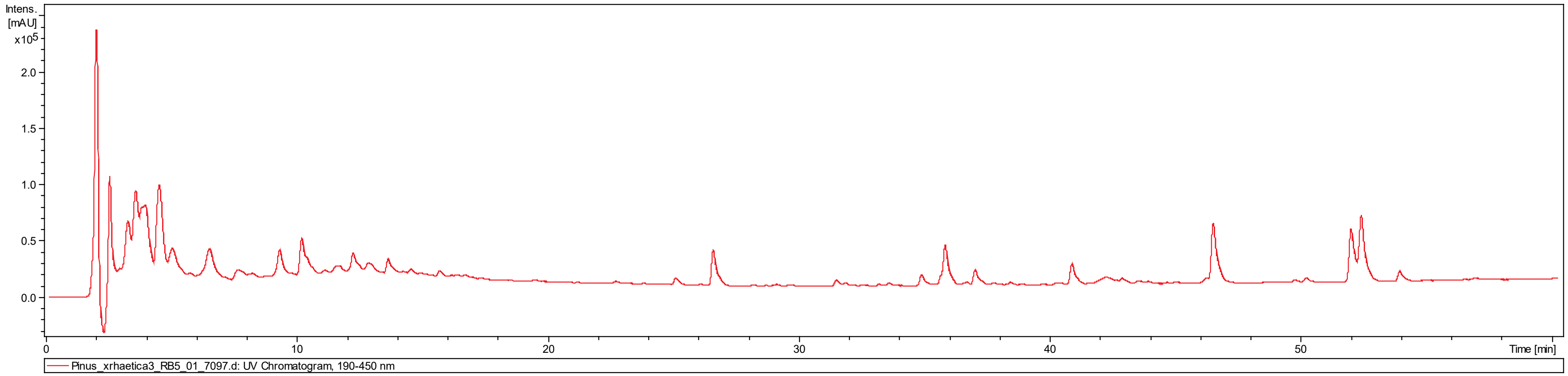

Figure S6. LC-DAD chromatogram of *Pinus ×rhaetica* Brügger branch wood methanolic extract taken at 190-450 nm.

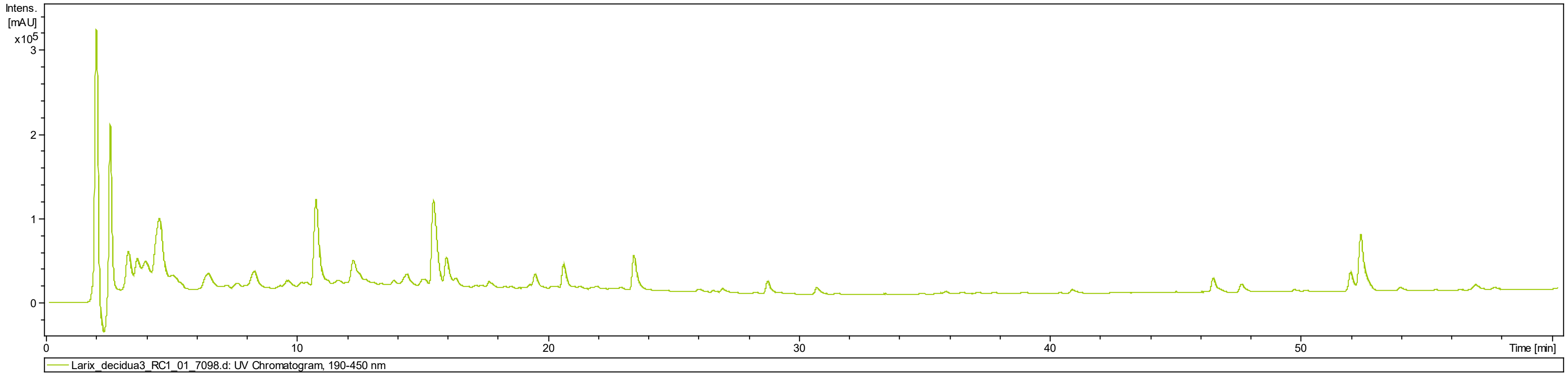

Figure S7. LC-DAD chromatogram of *Larix decidua* Mill. branch wood methanolic extract taken at 190-450 nm.

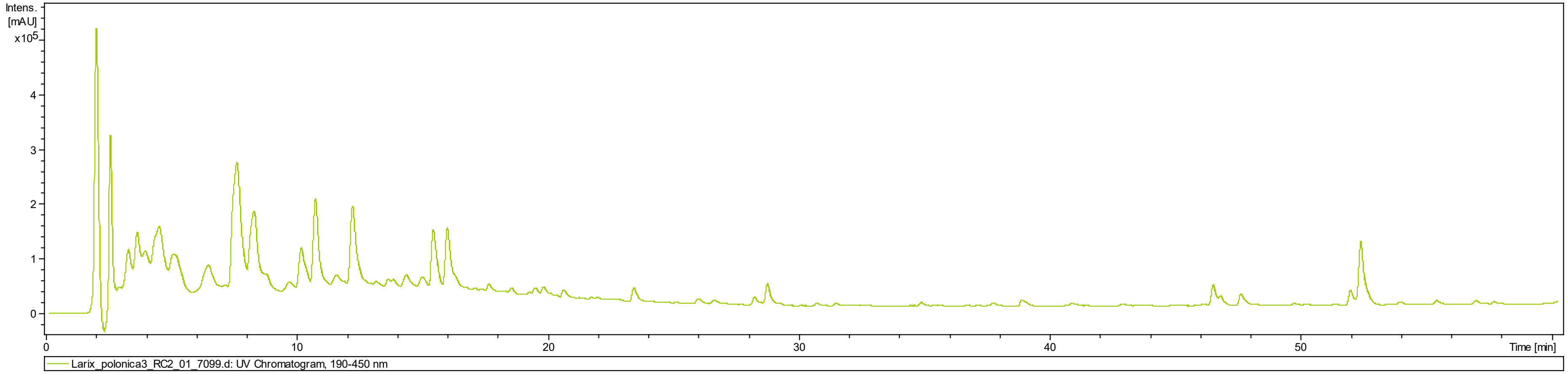

Figure S8. LC-DAD chromatogram of *Larix polonica* Rac. branch wood methanolic extract taken at 190-450 nm.

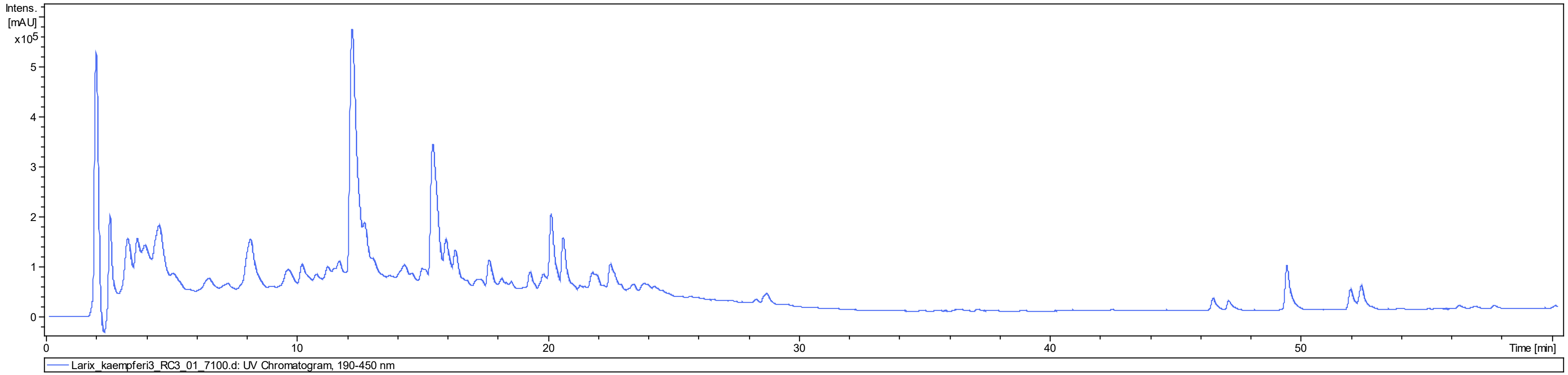

Figure S9. LC-DAD chromatogram of *Larix kaempferi* Lamb. branch wood methanolic extract taken at 190-450 nm.

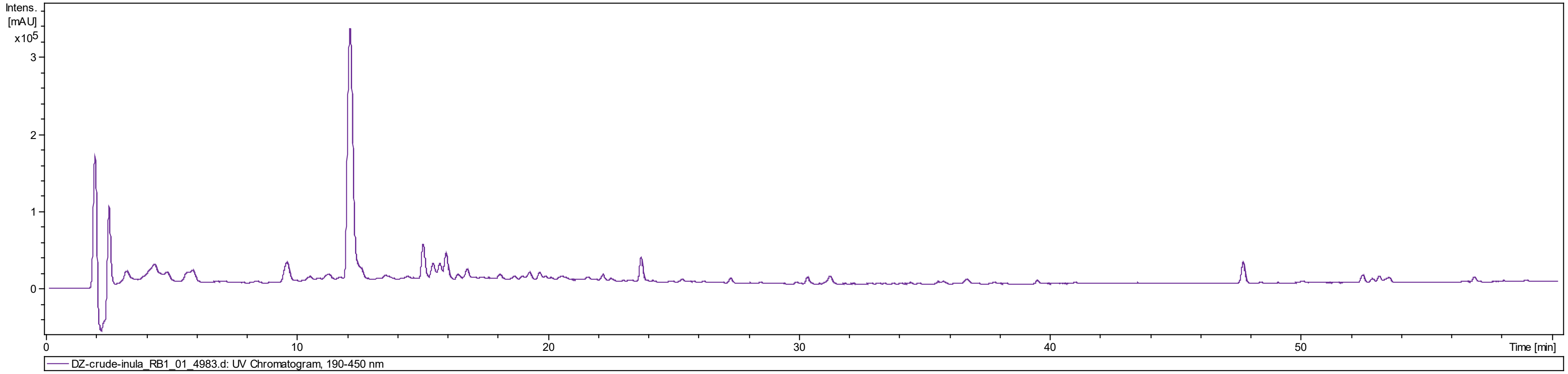

Figure S10. LC-DAD chromatogram of *Pseudotsuga menziesii* Mirb. branch wood methanolic extract taken at 190-450 nm.

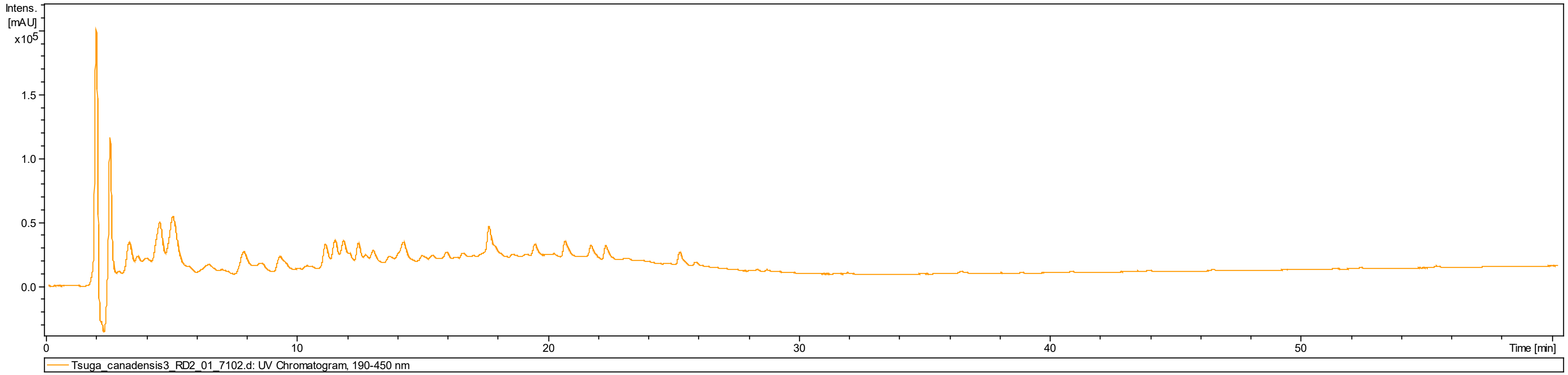

Figure S11. LC-DAD chromatogram of *Tsuga canadensis* Carrière branch wood methanolic extract taken at 190-450 nm.

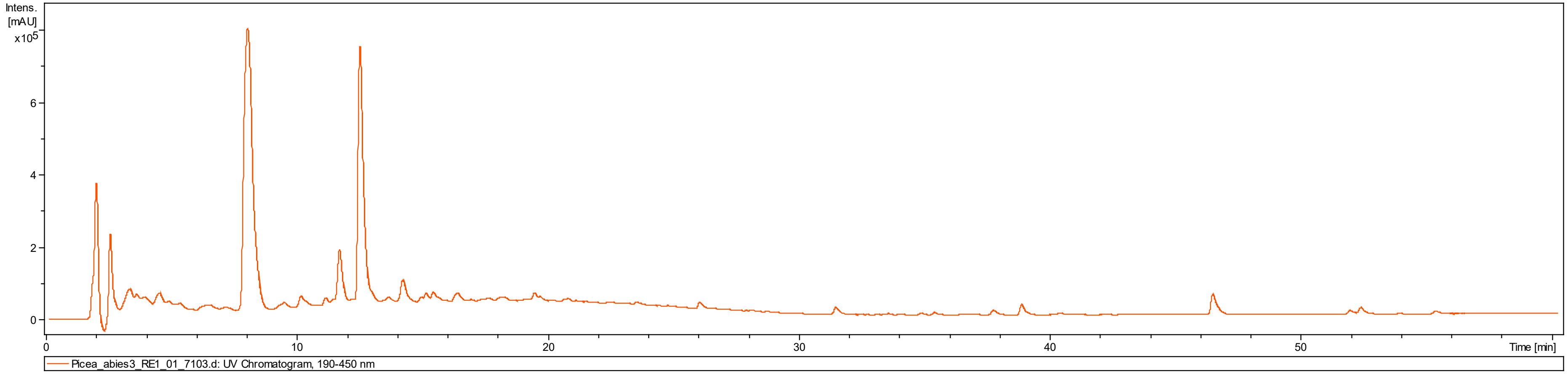

Figure S12. LC-DAD chromatogram of *Picea abies* L. branch wood methanolic extract taken at 190-450 nm.

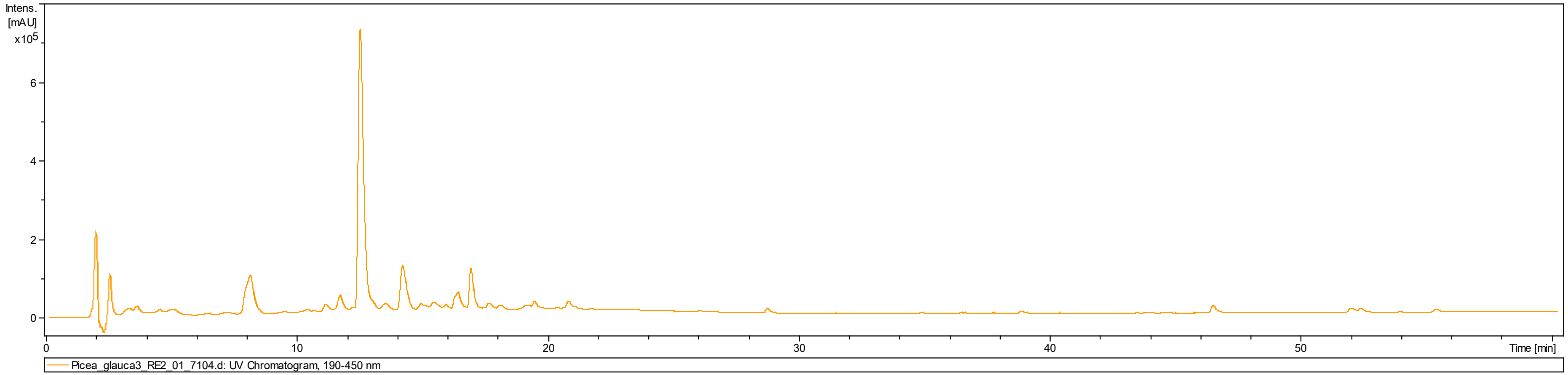

Figure S13. LC-DAD chromatogram of *Picea glauca* (Moench) Voss branch wood methanolic extract taken at 190-450 nm.

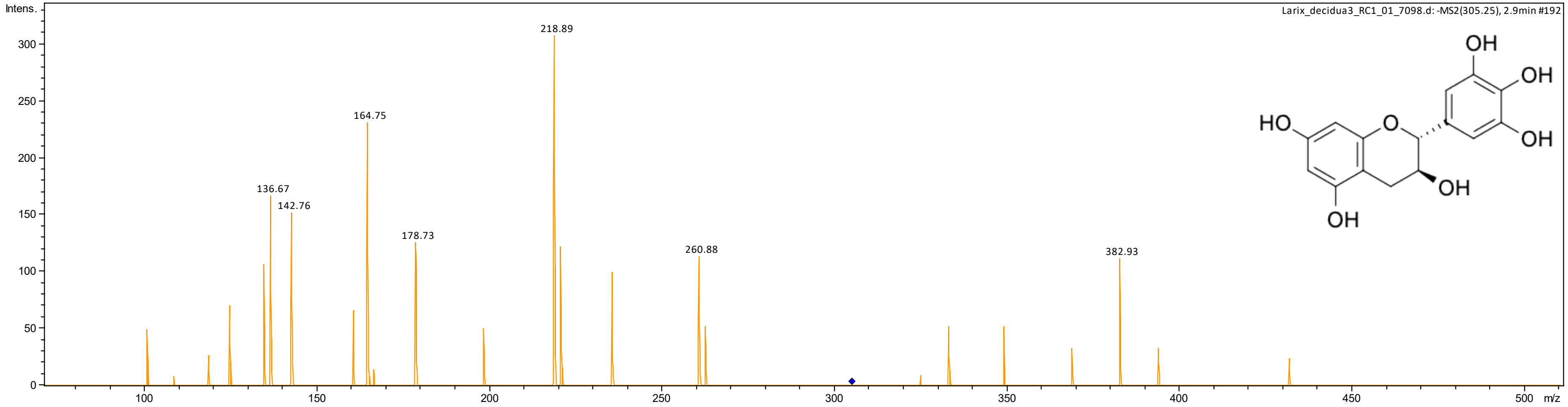

Figure S14. ESI-MS² spectrum of molecular ion at m/z 305 – compound (**1**).

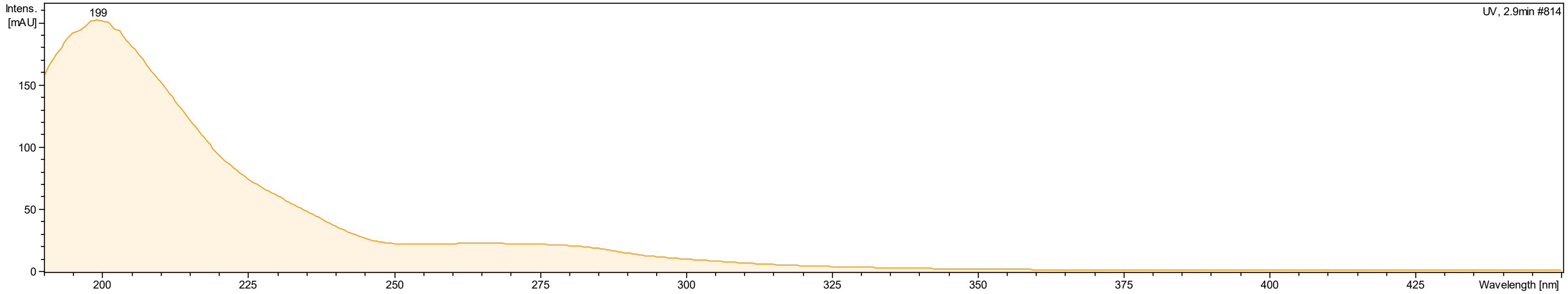

Figure S15. UV spectrum of compound (**1**).

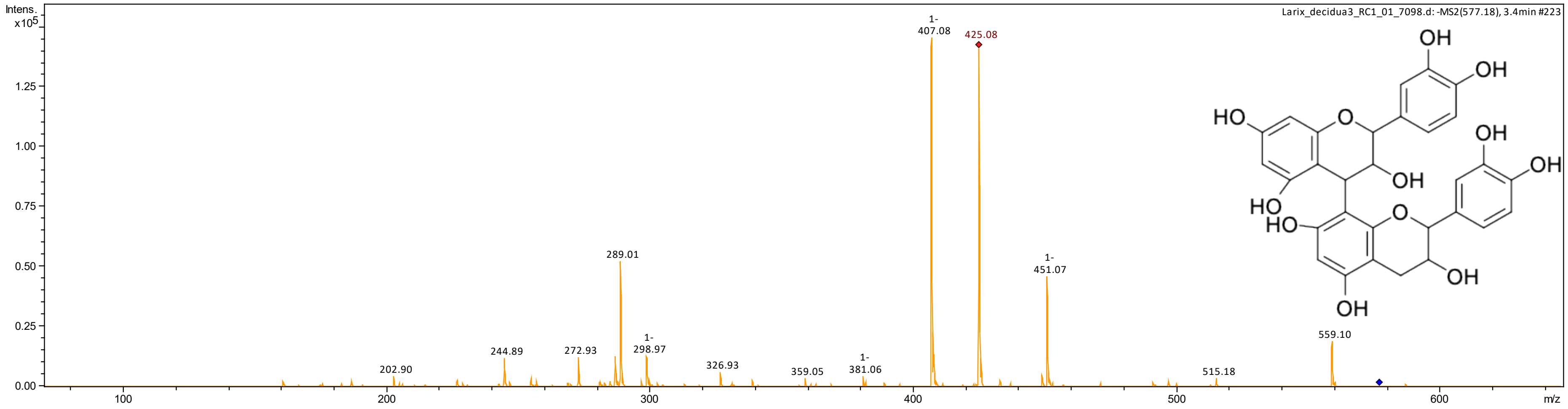

Figure S16. ESI-MS² spectrum (negative ion mode) of molecular ion at m/z 577 – compound (**2**).

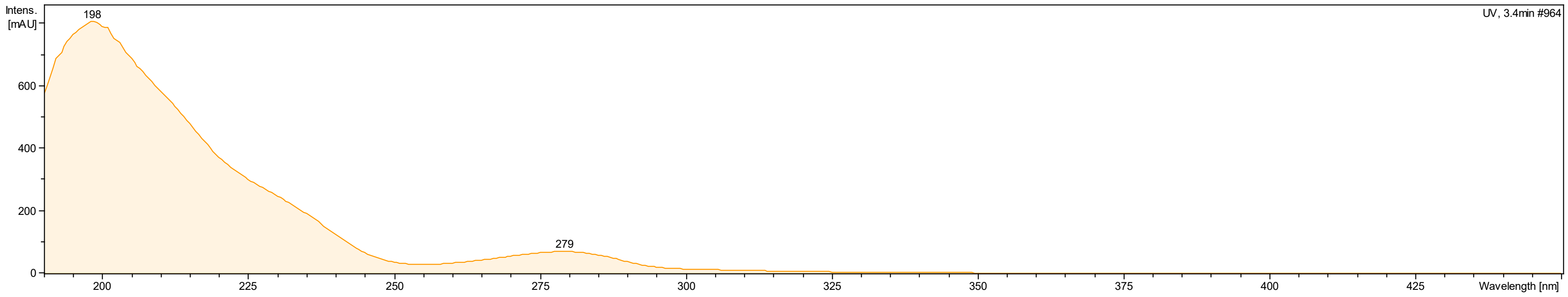

Figure S17. UV spectrum of compound (**2**).

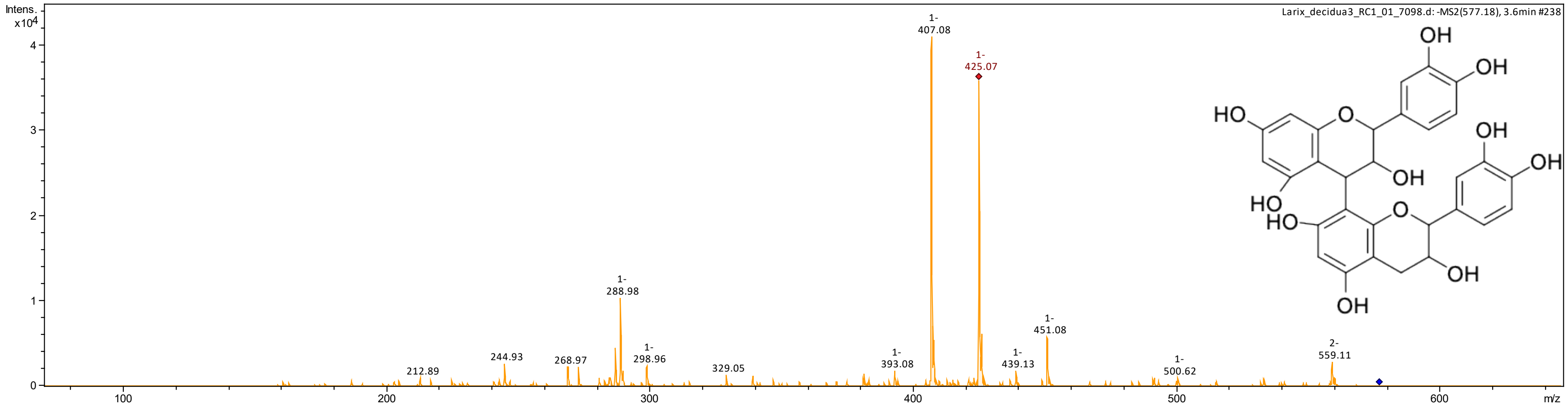

Figure S18. ESI-MS² spectrum (negative ion mode) of molecular ion at m/z 577 – compound (**3**).

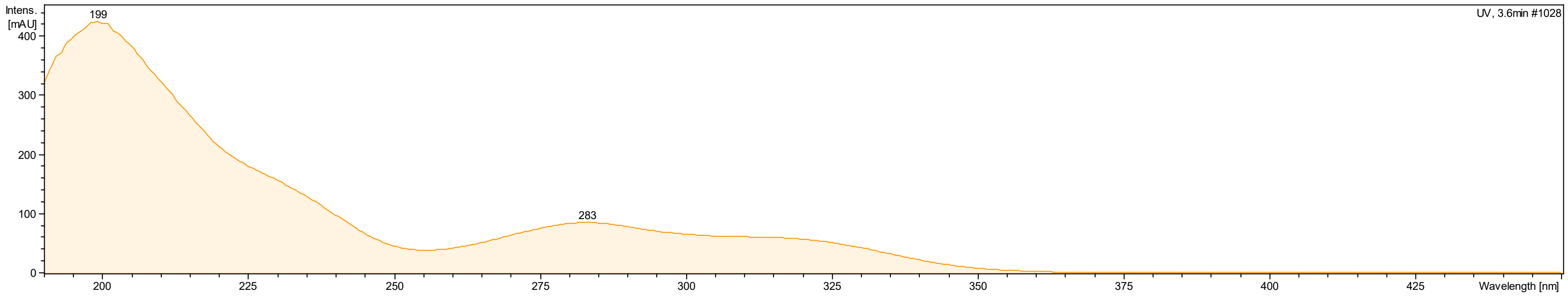

Figure S19. UV spectrum of compound (**3**).

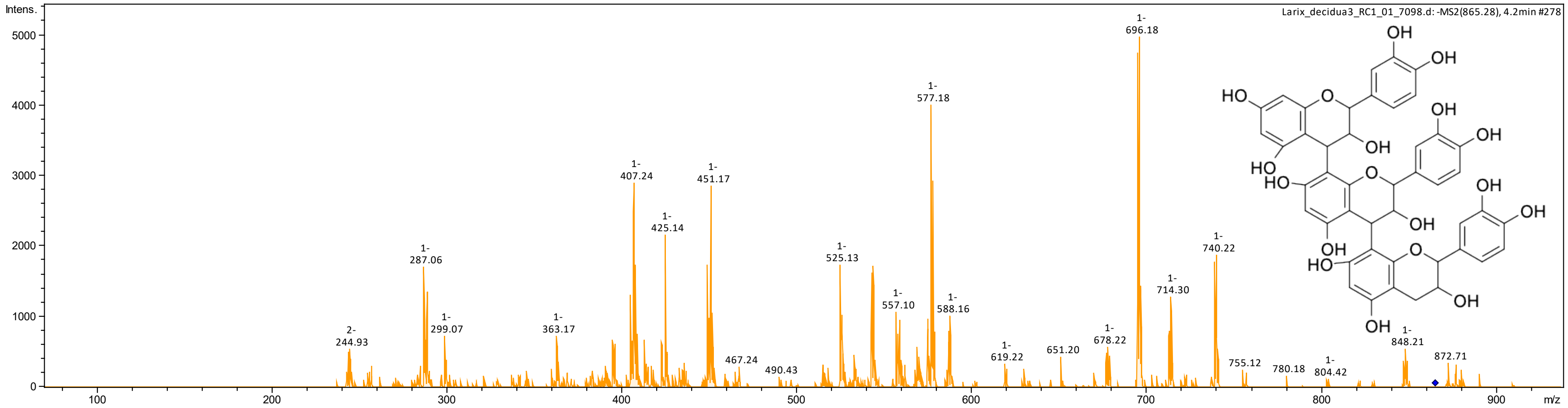

Figure S20. ESI-MS² spectrum (negative ion mode) of molecular ion at m/z 865 – compound (**4**).

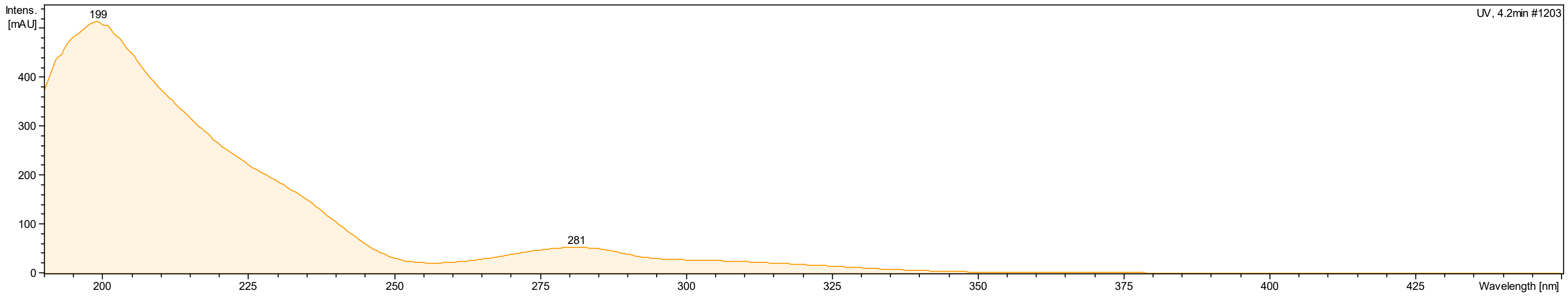

Figure S21. UV spectrum of compound (**4**).

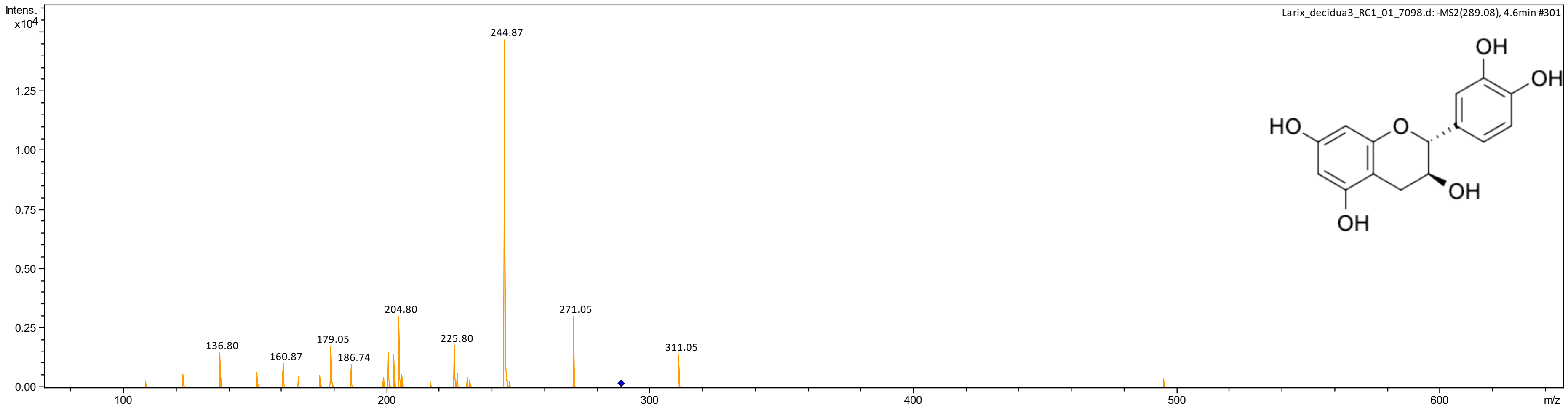

Figure S22. ESI-MS² spectrum (negative ion mode) of molecular ion at m/z 289 – compound (**5**).

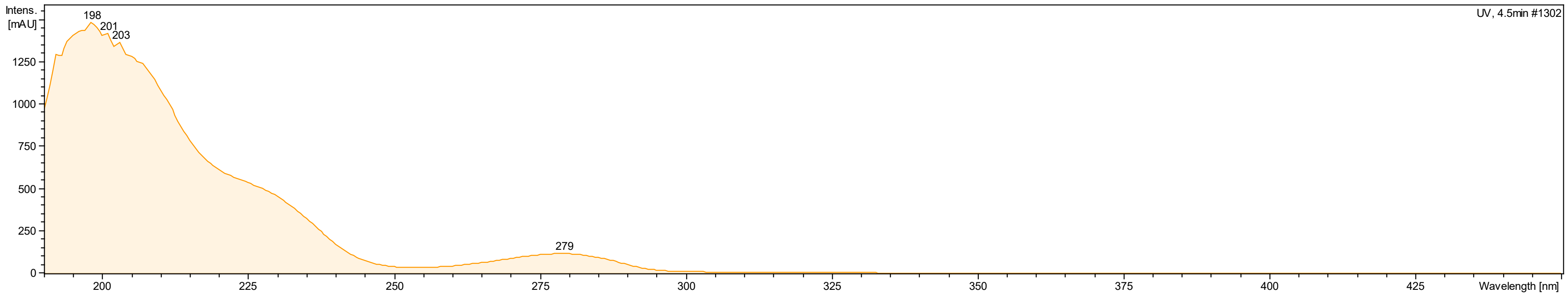

Figure S23. UV spectrum of compound (**5**).

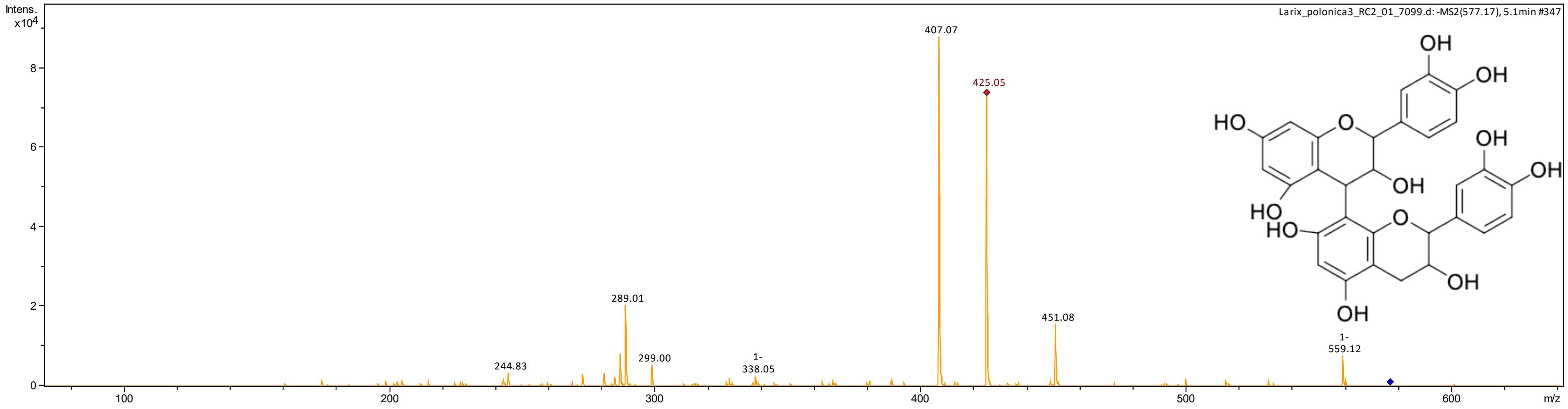

Figure S24. ESI-MS² spectrum (negative ion mode) of molecular ion at m/z 577 – compound (**6**).

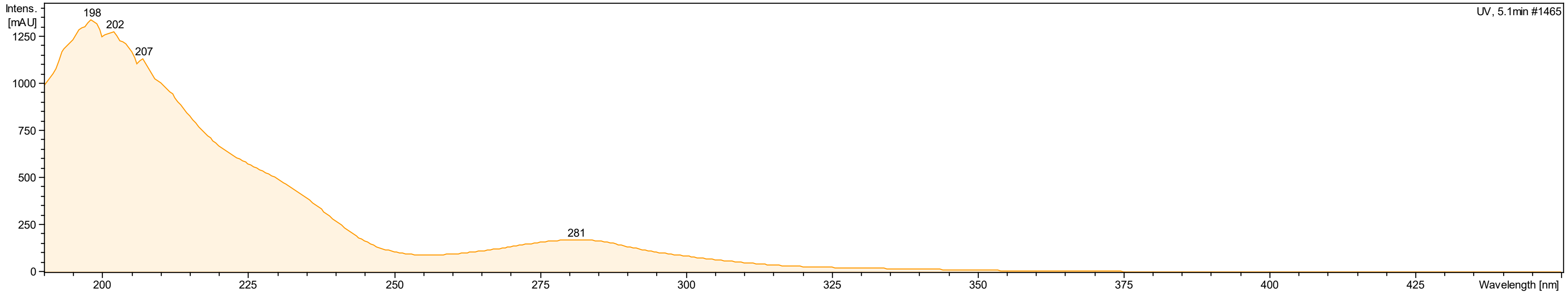

Figure S25. UV spectrum of compound (**6**).

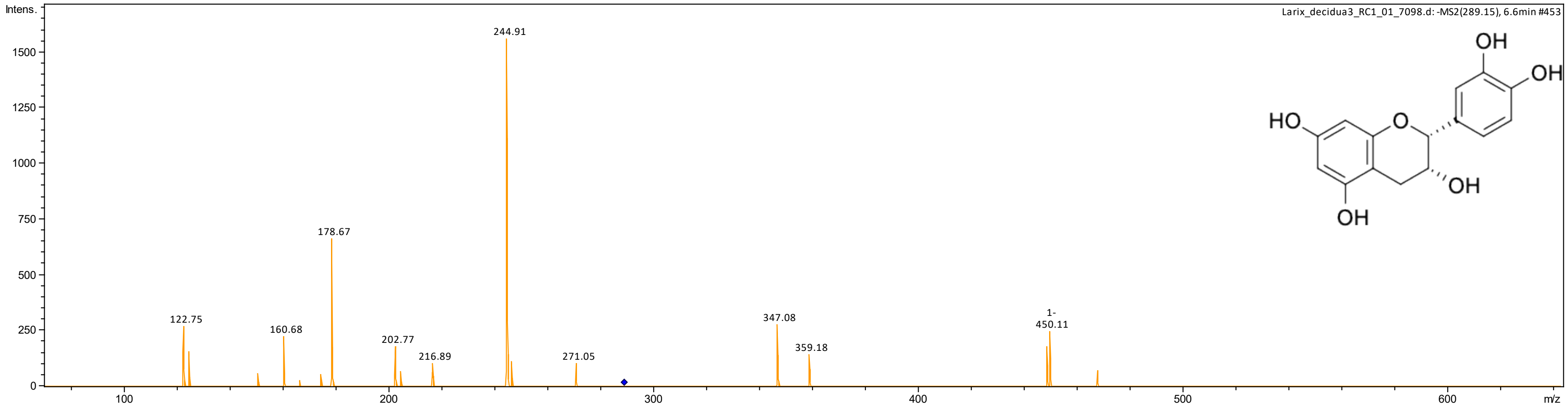

Figure S26. ESI-MS² spectrum (negative ion mode) of molecular ion at m/z 289 – compound (**7**).

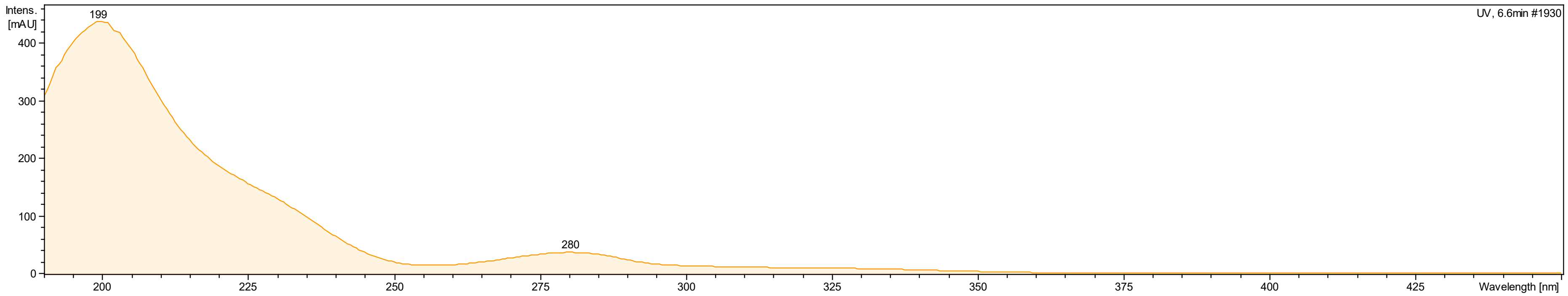

Figure S27. UV spectrum of compound (**7**).

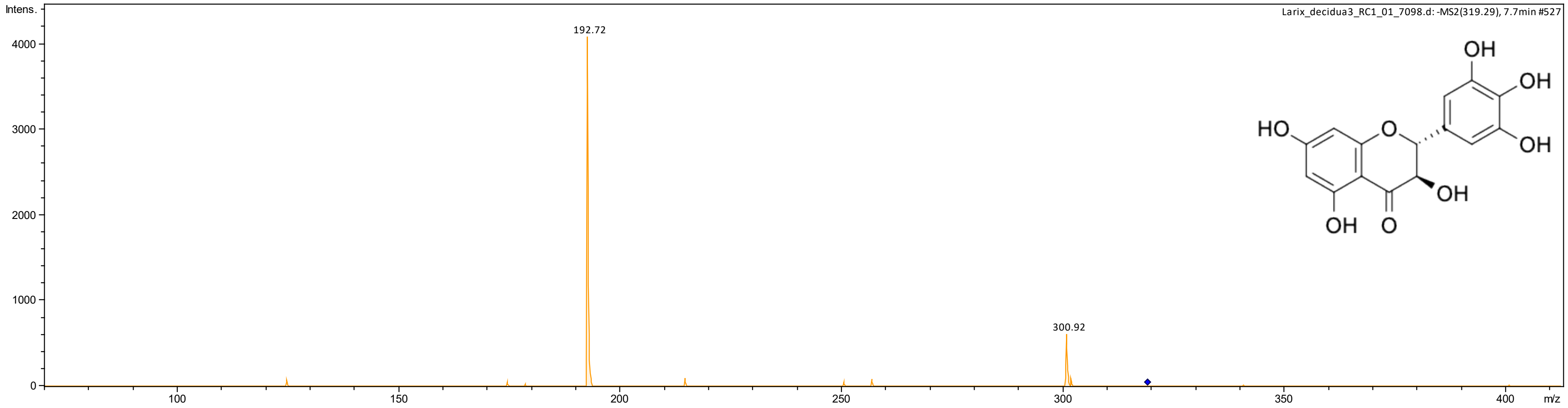

Figure S28. ESI-MS² spectrum (negative ion mode) of molecular ion at m/z 319 – compound (**8**).

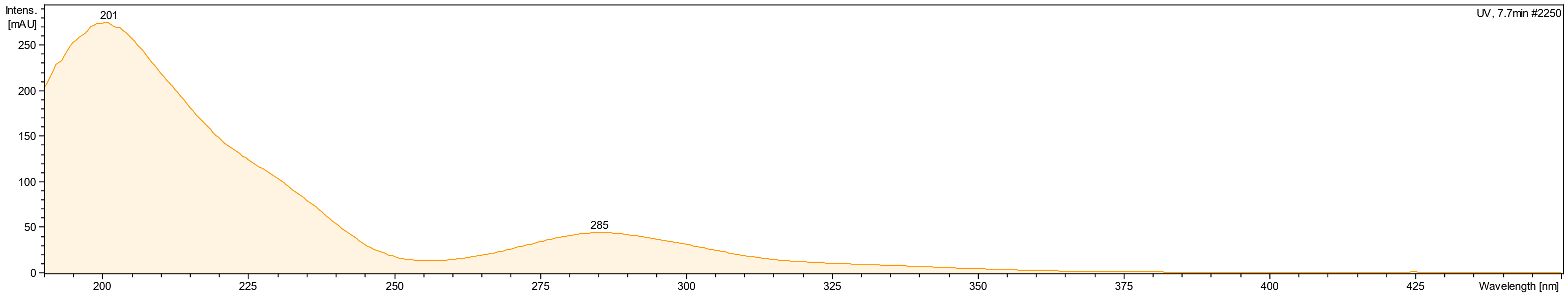

Figure S29. UV spectrum of compound (**8**).

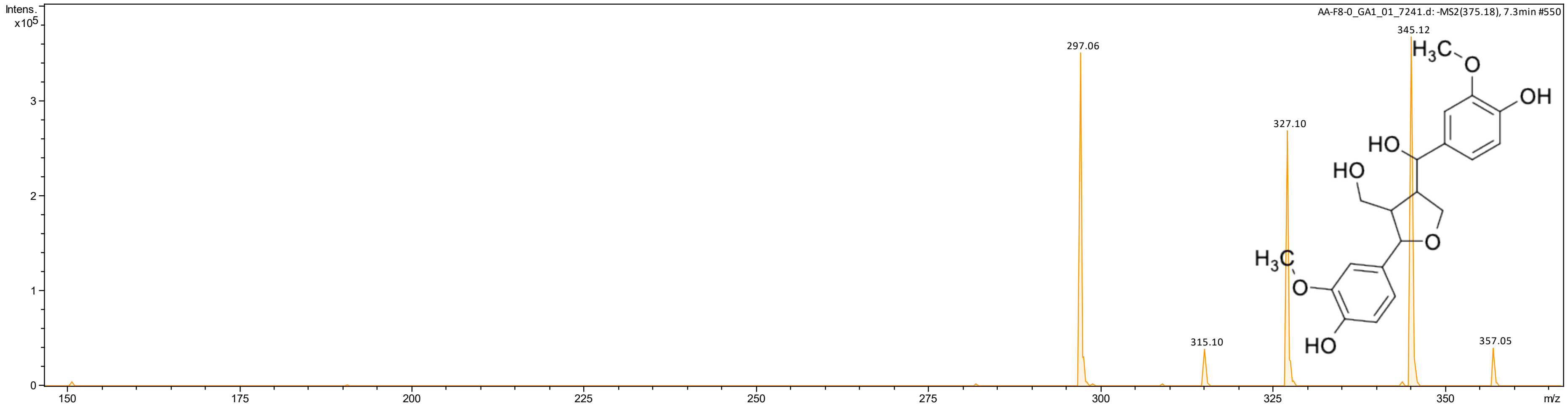

Figure S30. ESI-MS² spectrum (negative ion mode) of molecular ion at m/z 375 – compound (**9**).

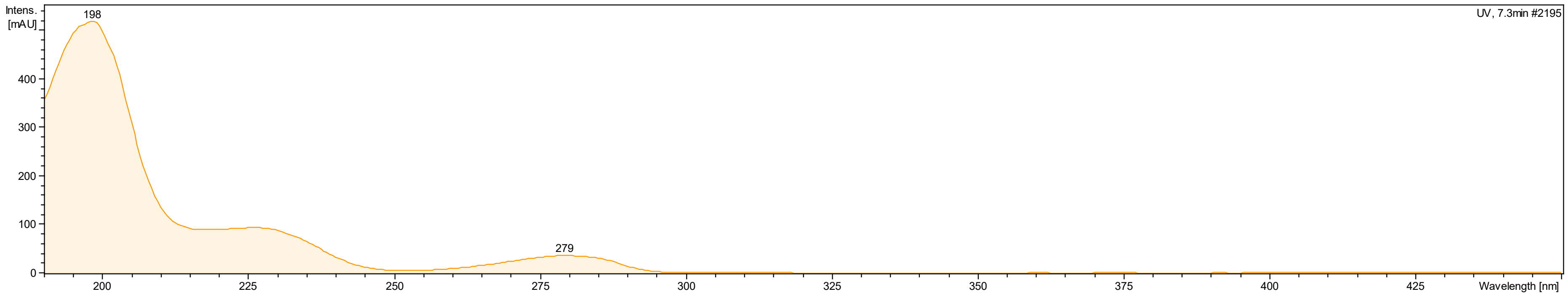

Figure S31. UV spectrum of compound (**9**).

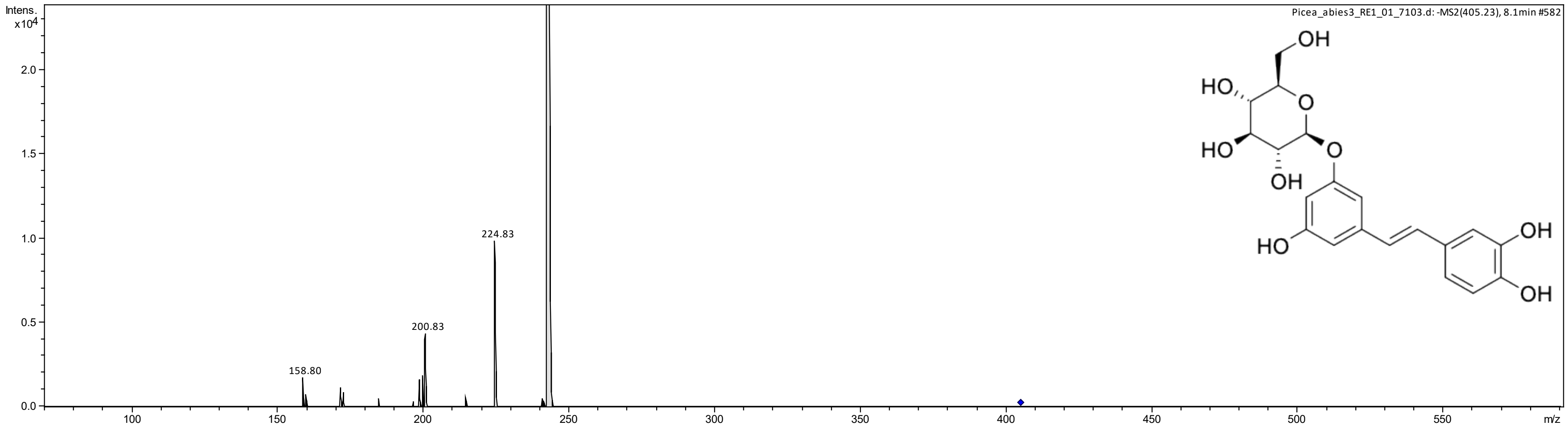

Figure S32. ESI-MS² spectrum (negative ion mode) of molecular ion at m/z 405 – compound (**10**).

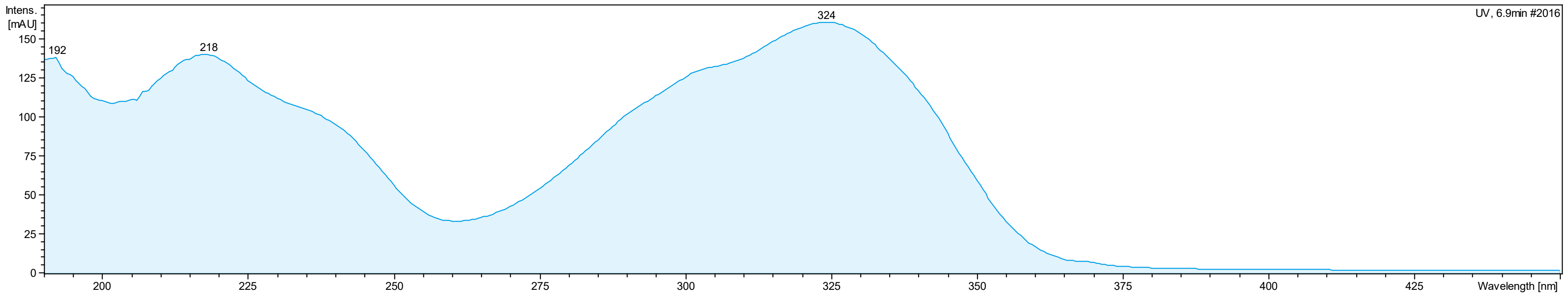

Figure S33. UV spectrum of compound (**10**).

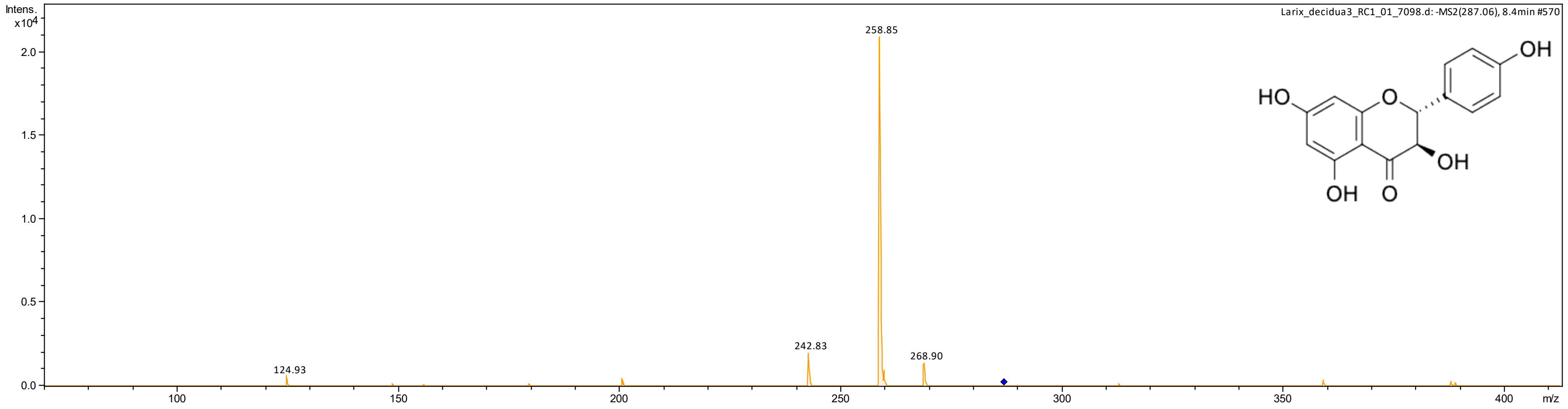

Figure S34. ESI-MS² spectrum (negative ion mode) of molecular ion at m/z 287 – compound (**11**).

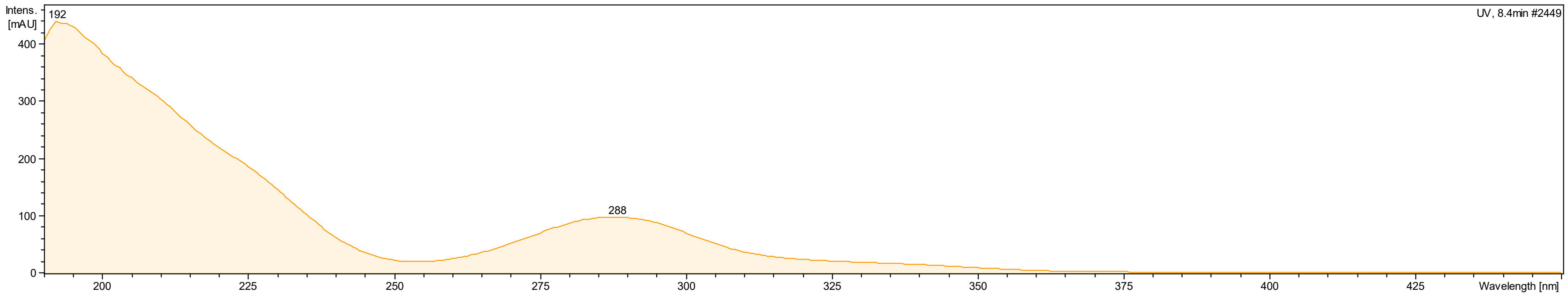

Figure S35. UV spectrum of compound (**11**).

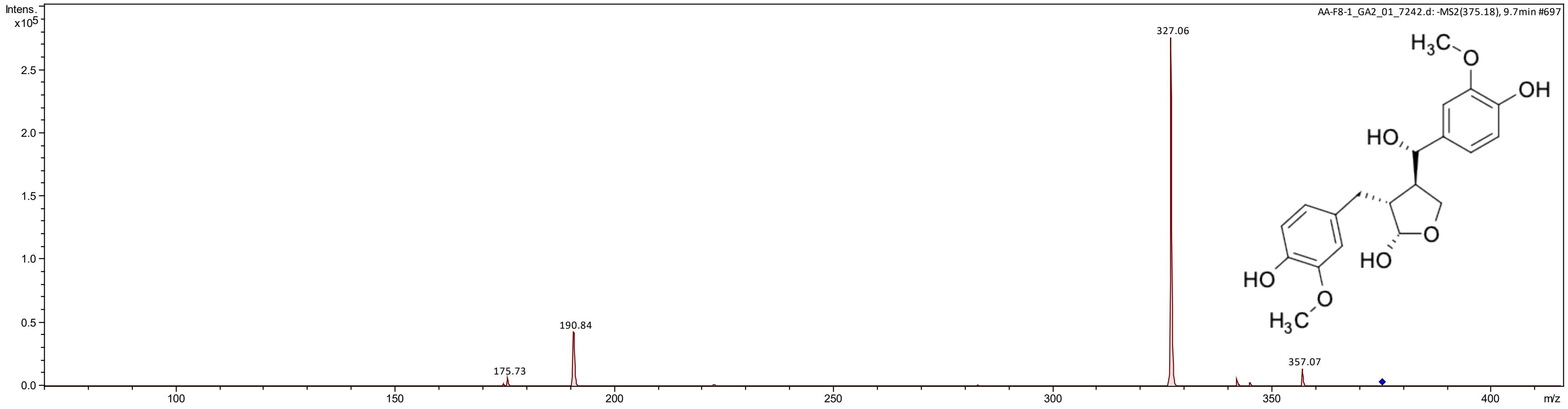

Figure S36. ESI-MS² spectrum (negative ion mode) of molecular ion at m/z 375 – compound (**12**).

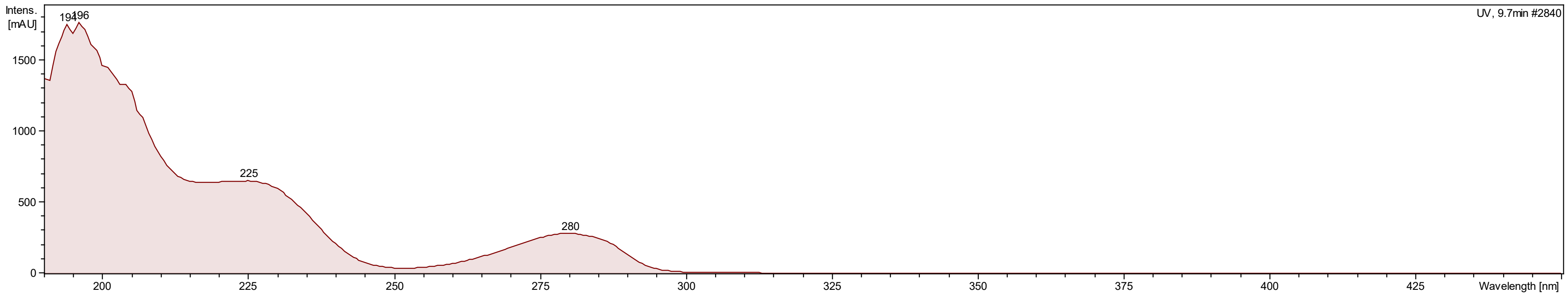

Figure S37. UV spectrum of compound (**12**).

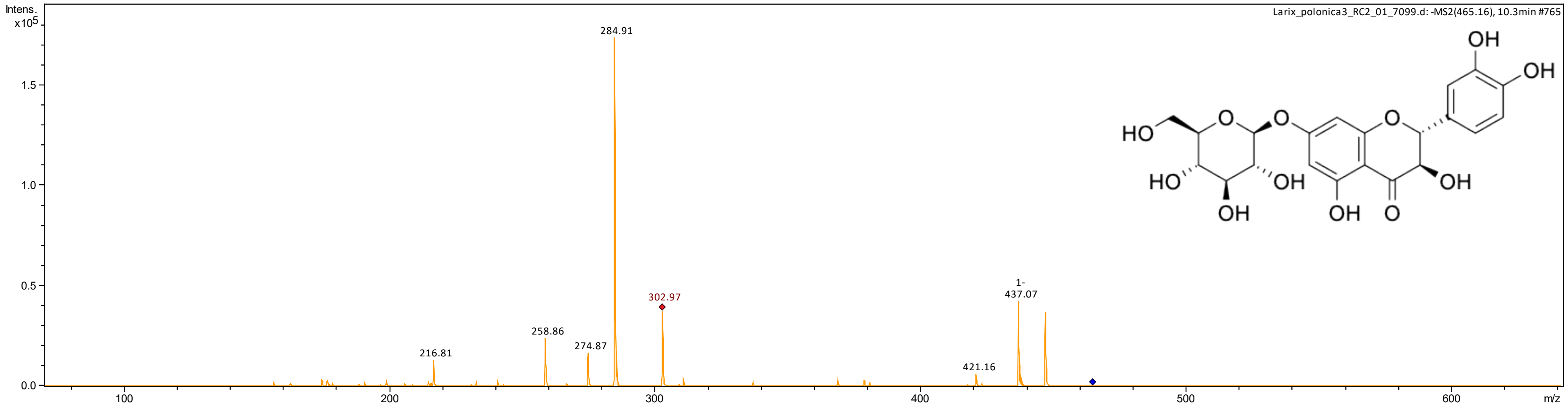

Figure S38. ESI-MS² spectrum (negative ion mode) of molecular ion at m/z 465 – compound (**13**).

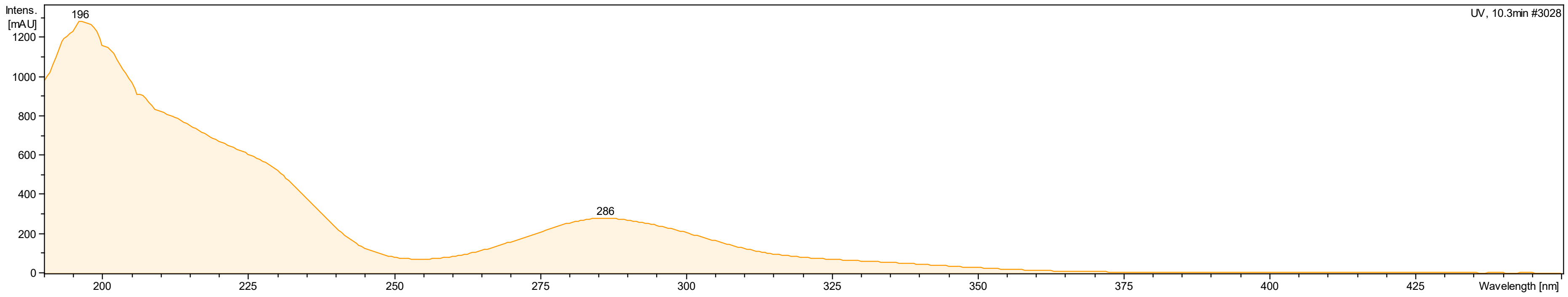

Figure S39. UV spectrum of compound (**13**).

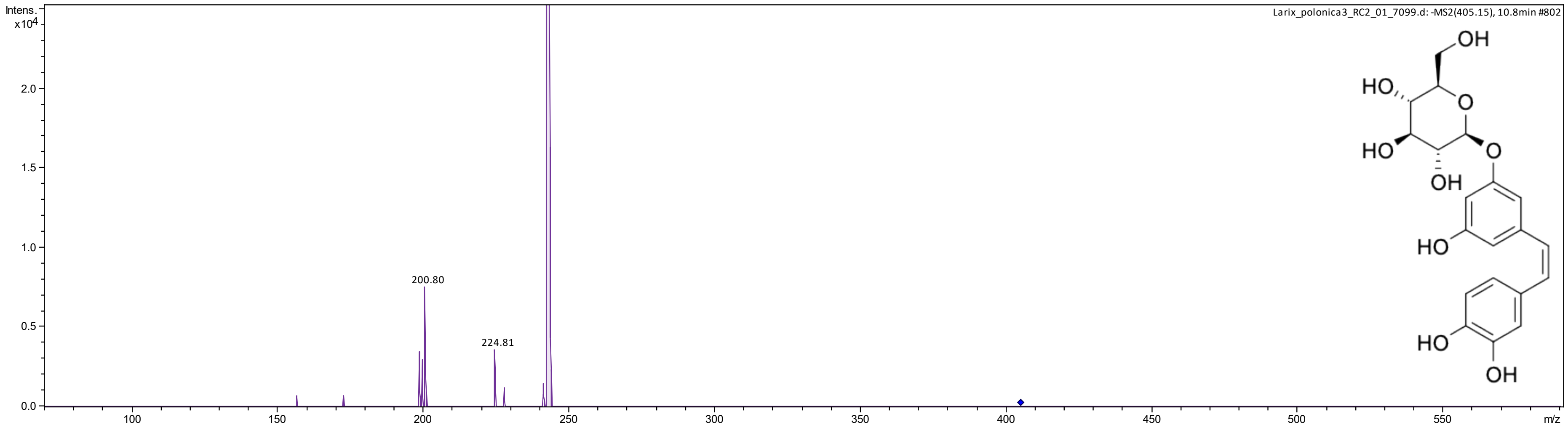

Figure S40. ESI-MS² spectrum (negative ion mode) of molecular ion at m/z 405 – compound (**14**).

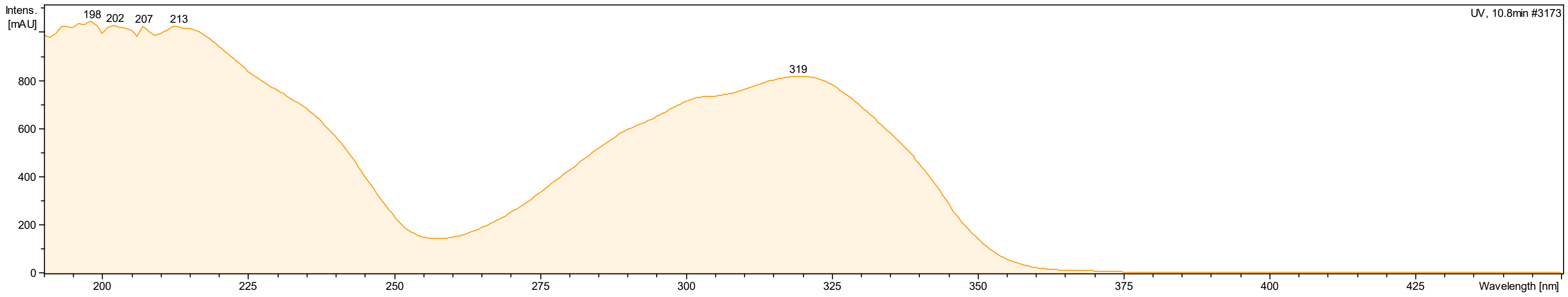

Figure S41. UV spectrum of compound (**14**).

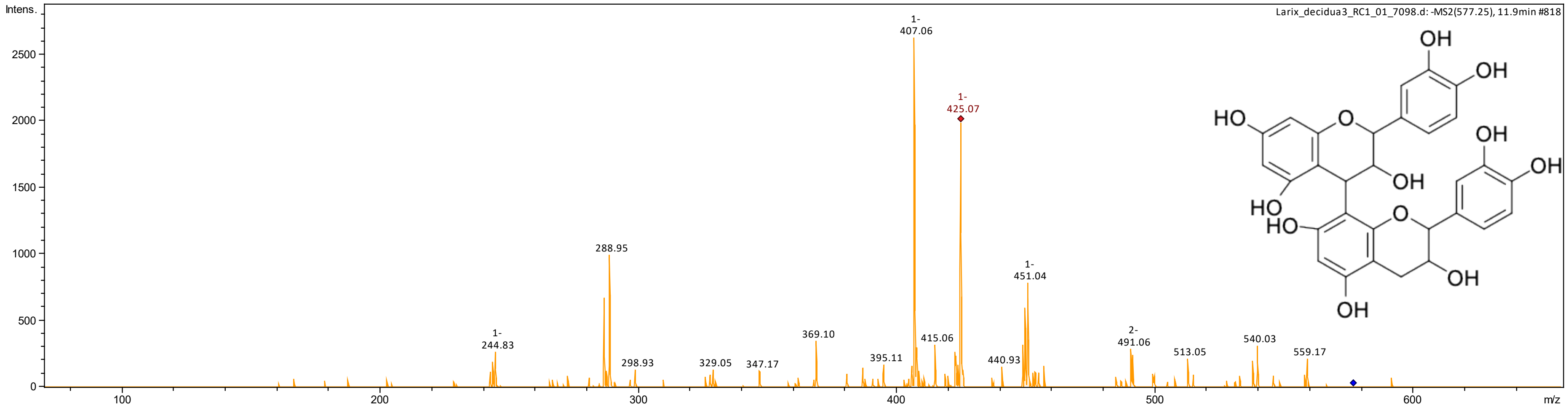

Figure S42. ESI-MS² spectrum (negative ion mode) of molecular ion at m/z 577 – compound (**15**).

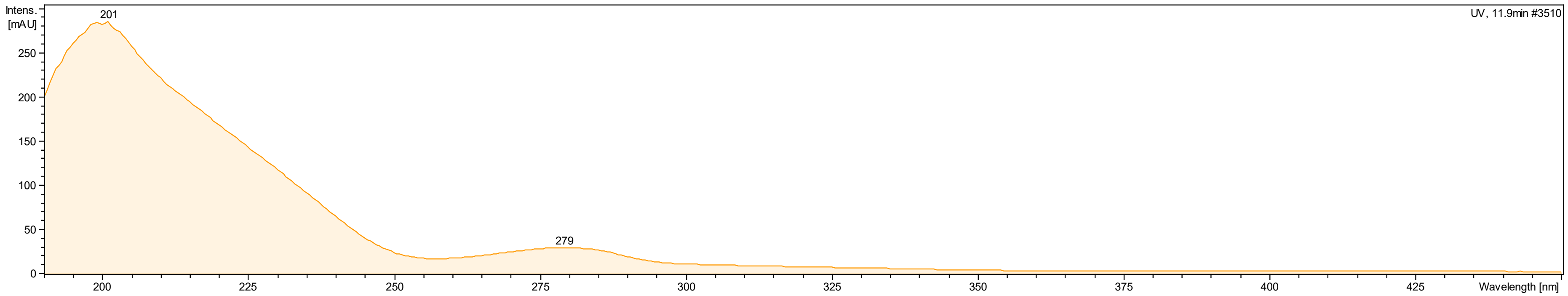

Figure S43. UV spectrum of compound (**15**).

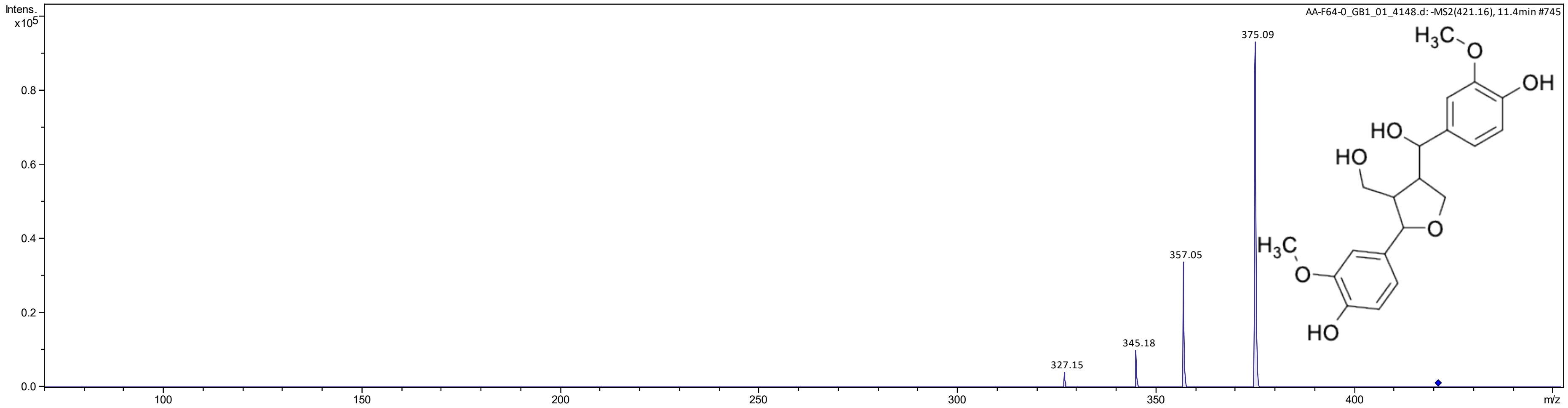

Figure S44. ESI-MS² spectrum (negative ion mode) of molecular ion at m/z 421 – compound (**16**).

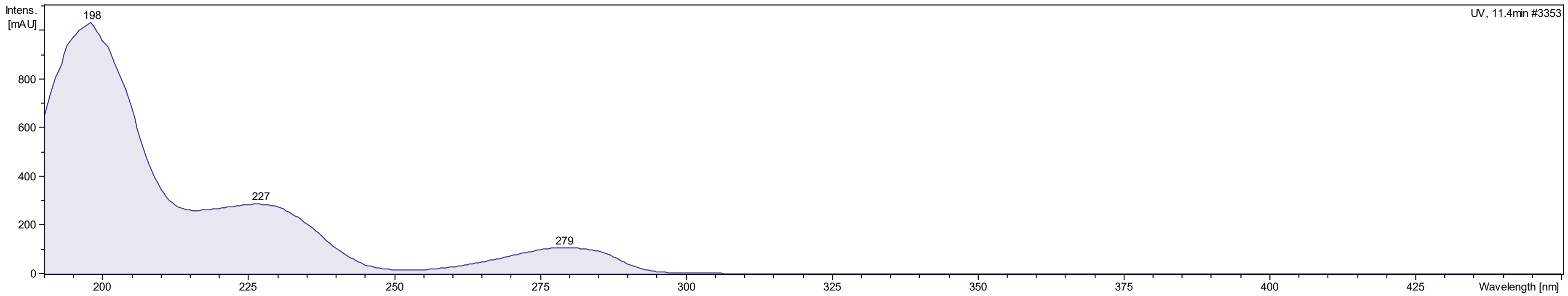

Figure S45. UV spectrum of compound (**16**).

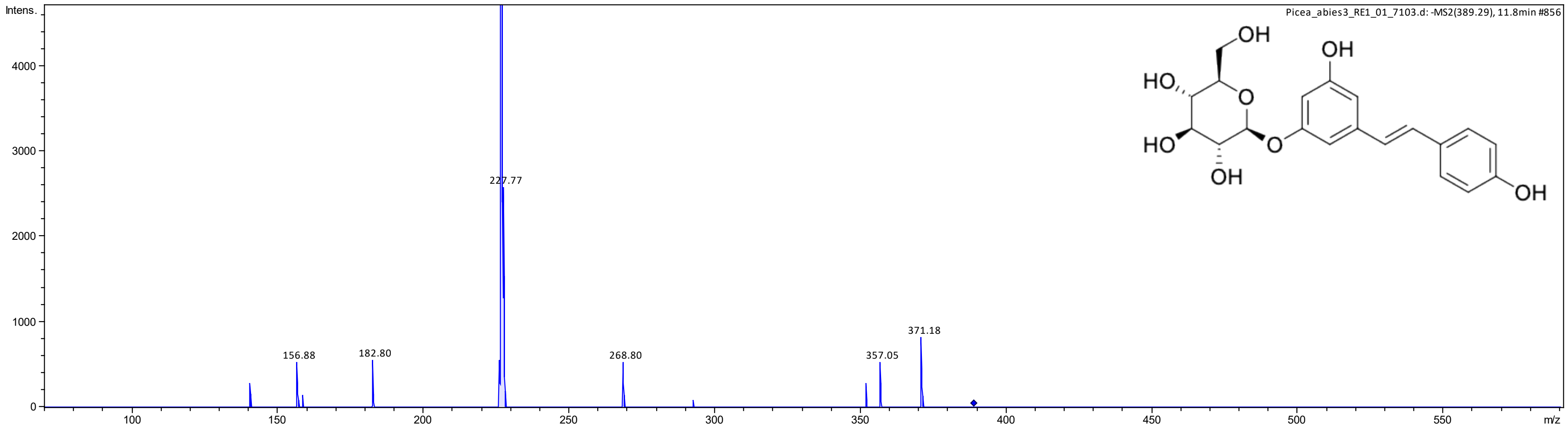

Figure S46. ESI-MS² spectrum (negative ion mode) of molecular ion at m/z 389 – compound (**17**).

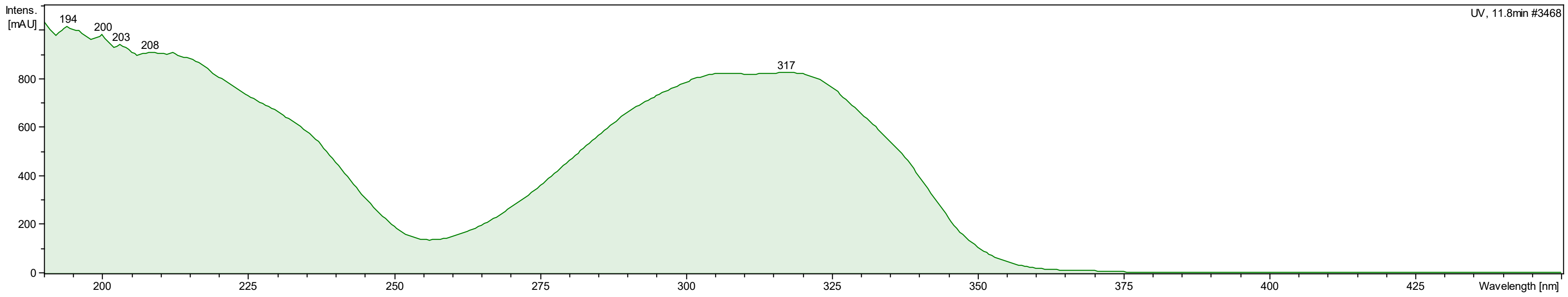

Figure S47. UV spectrum of compound (**17**).

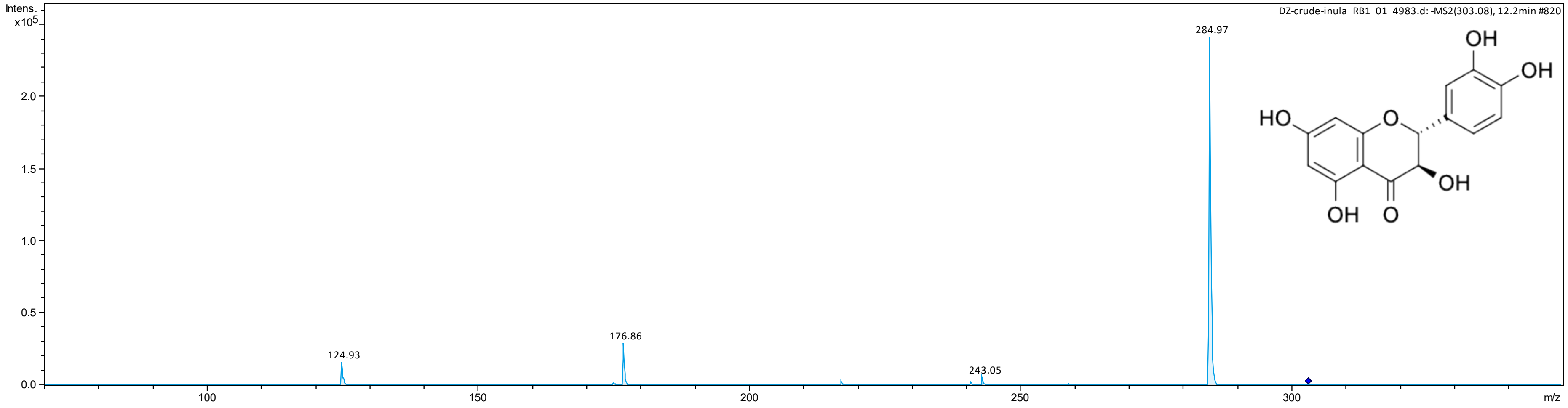

Figure S48. ESI-MS² spectrum (negative ion mode) of molecular ion at m/z 303 – compound (**18**).

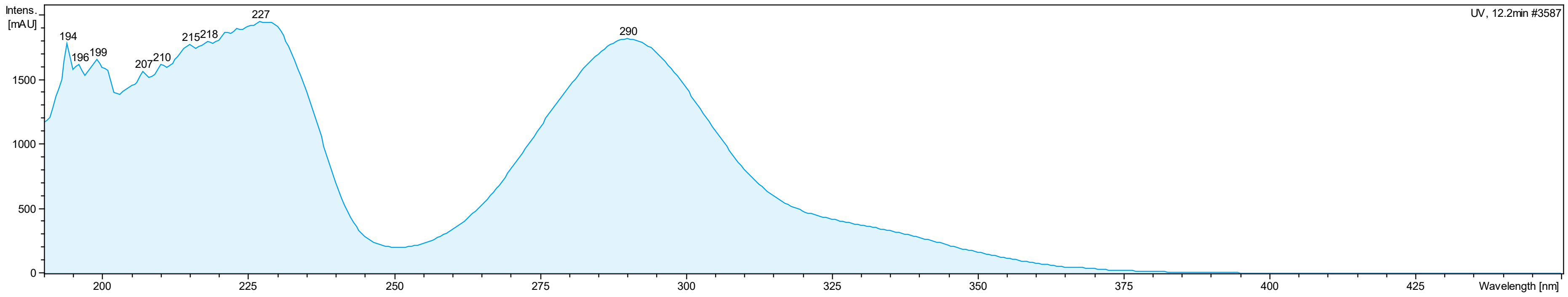

Figure S49. UV spectrum of compound (**18**).

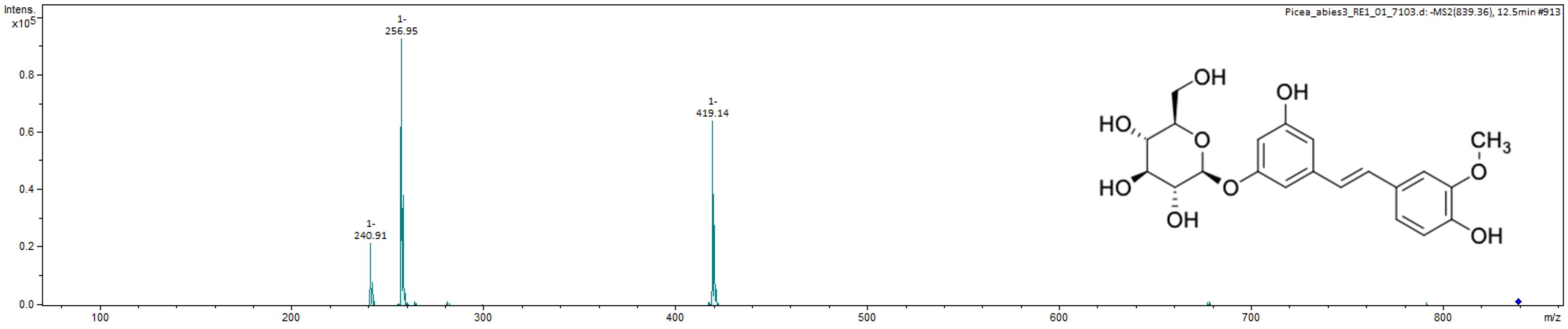

Figure S50. ESI-MS² spectrum (negative ion mode) of molecular ion at m/z 465 – compound (**19**).

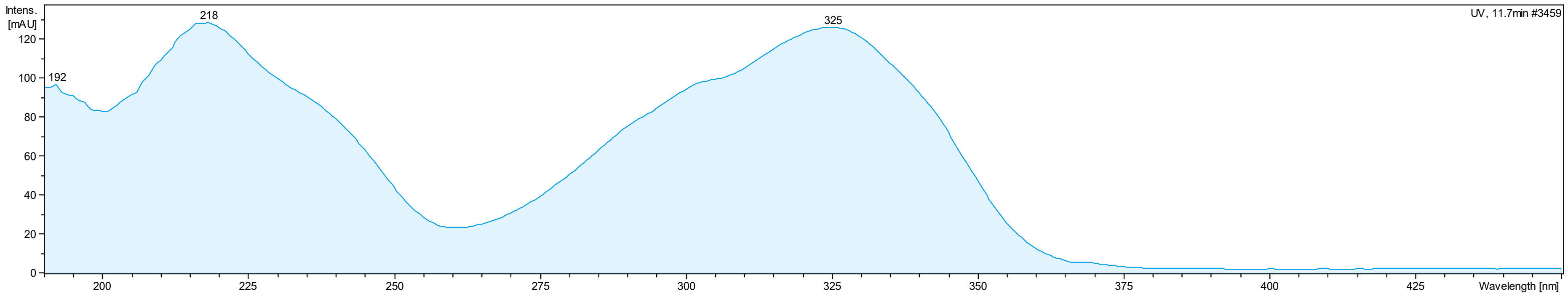

Figure S51. UV spectrum of compound (**19**).

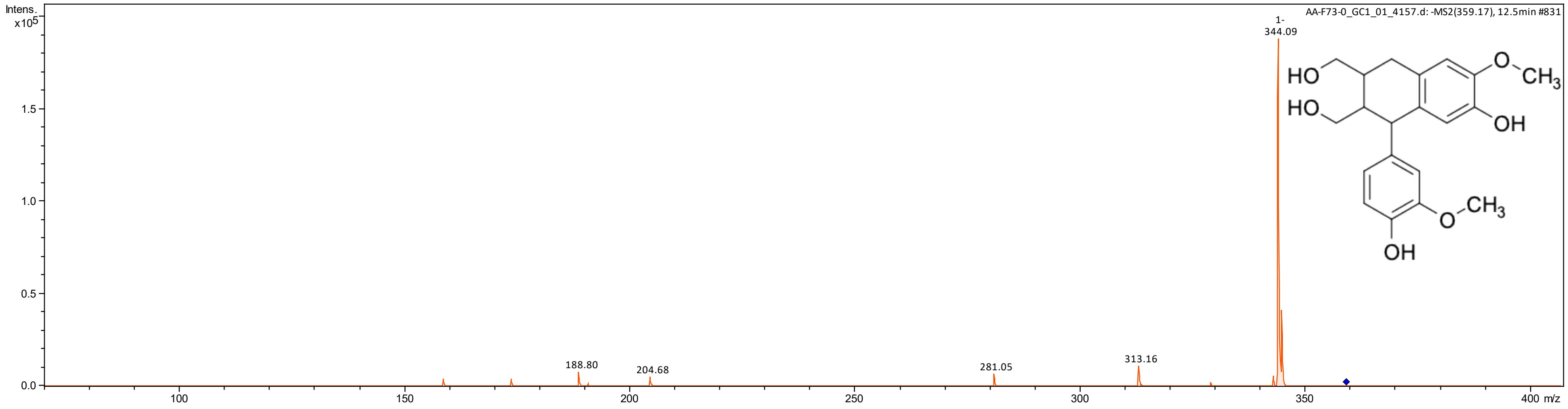

Figure S52. ESI-MS² spectrum (negative ion mode) of molecular ion at m/z 359 – compound (**20**).

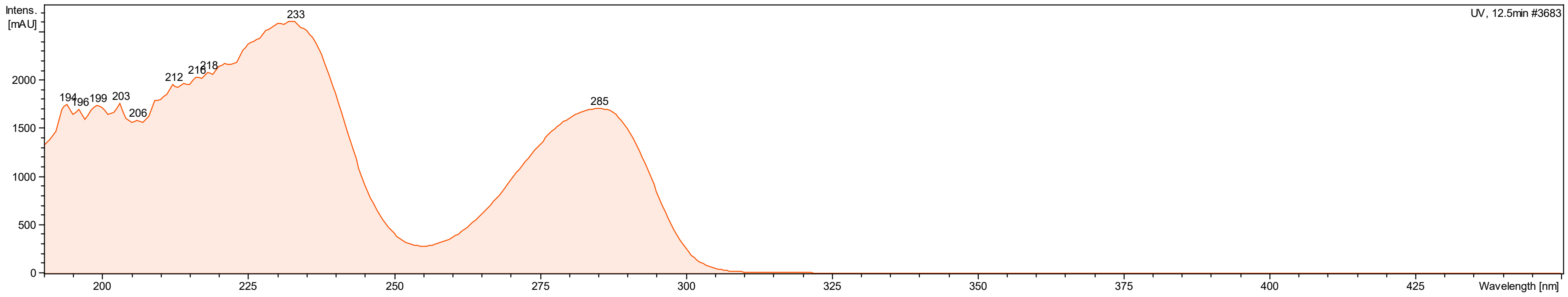

Figure S53. UV spectrum of compound (**20**).

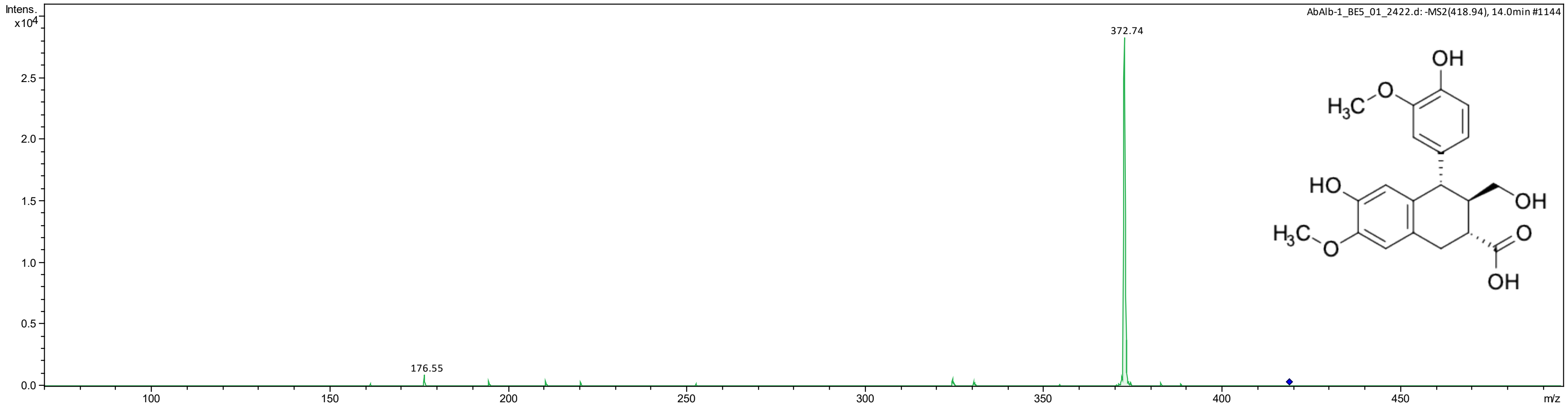

Figure S54. ESI-MS² spectrum (negative ion mode) of molecular ion at m/z 419 – compound (**21**).

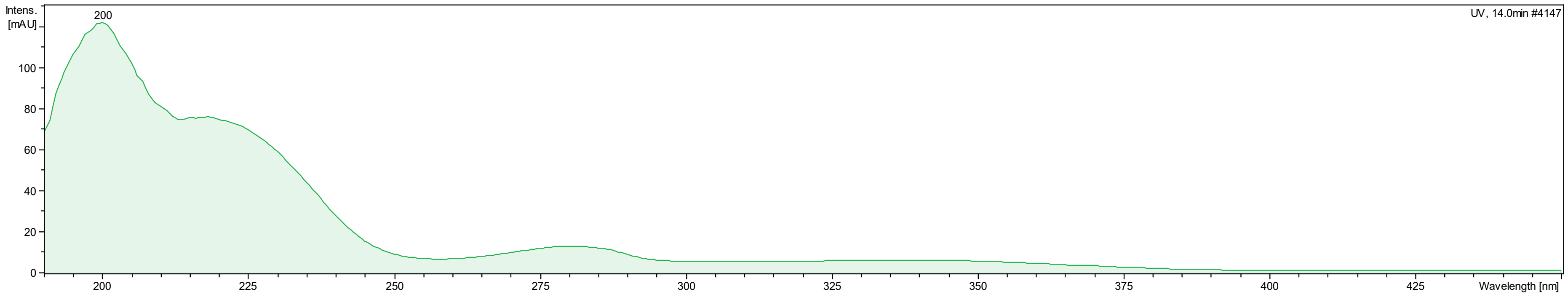

Figure S55. UV spectrum of compound (**21**).

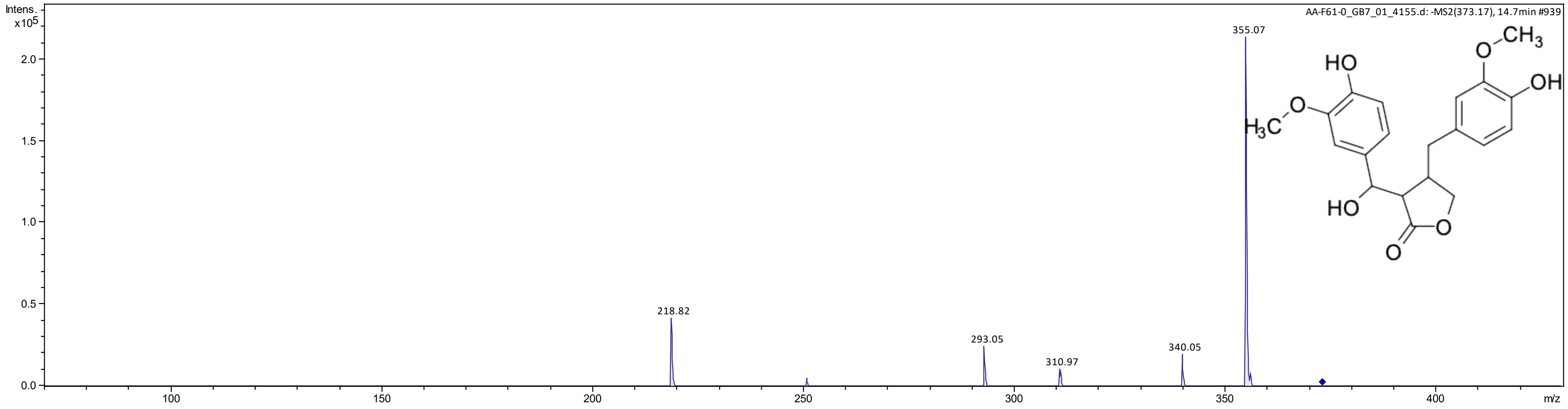

Figure S56. ESI-MS² spectrum (negative ion mode) of molecular ion at m/z 373 – compound (**22**).

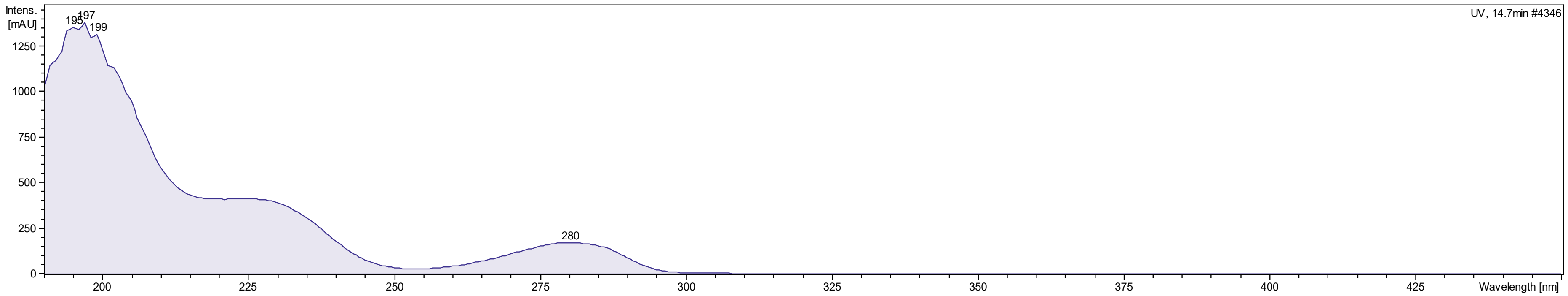

Figure S57. UV spectrum of compound (**22**).

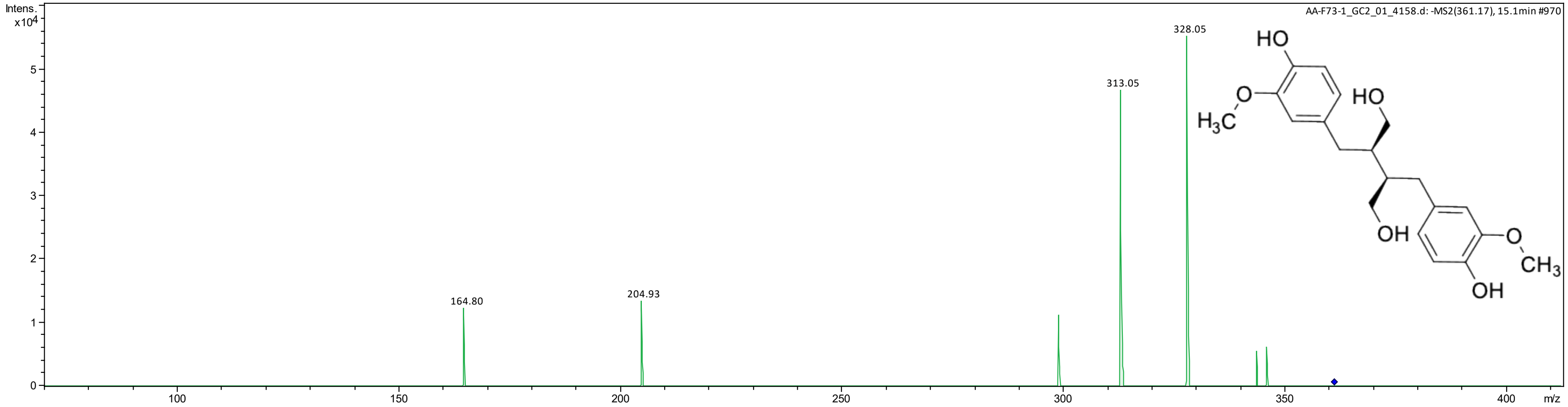

Figure S58. ESI-MS² spectrum (negative ion mode) of molecular ion at m/z 361 – compound (**23**).

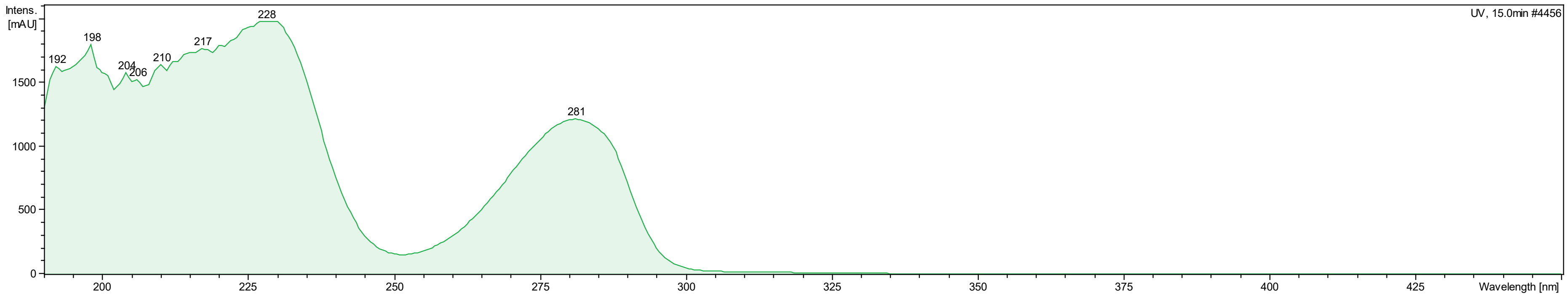

Figure S59. UV spectrum of compound (**23**).

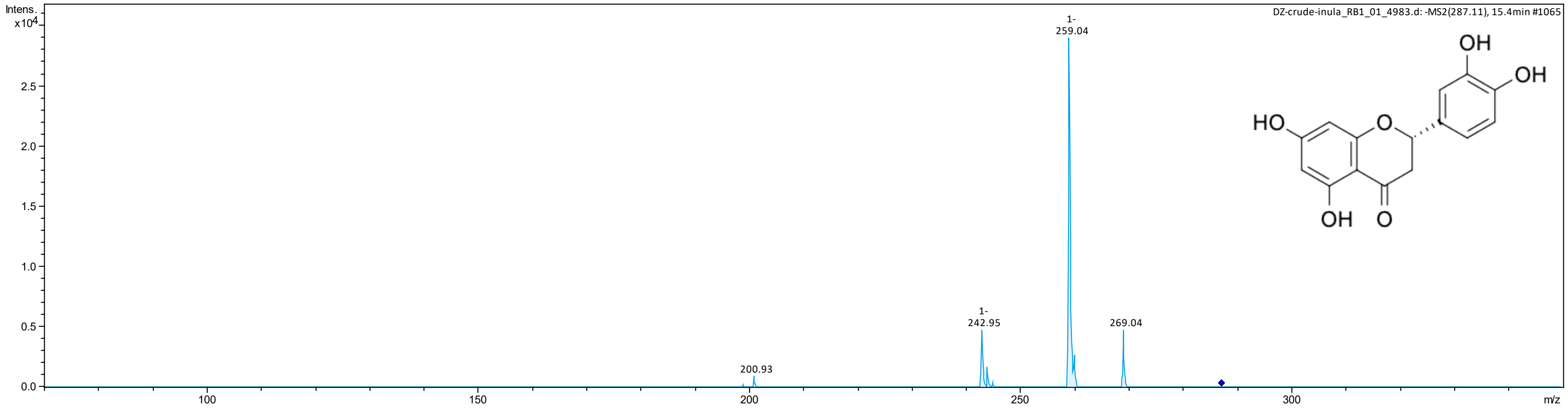

Figure S60. ESI-MS² spectrum (negative ion mode) of molecular ion at m/z 287 – compound (**24**).

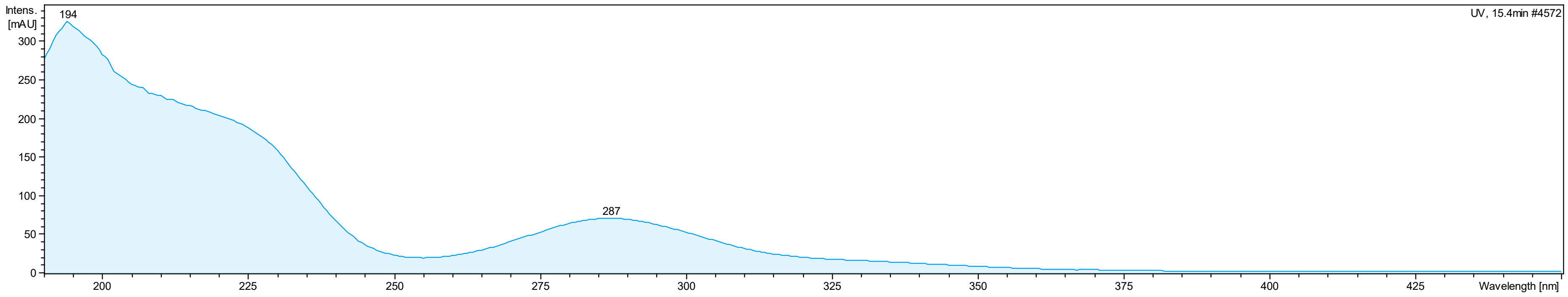

Figure S61. UV spectrum of compound (**24**).

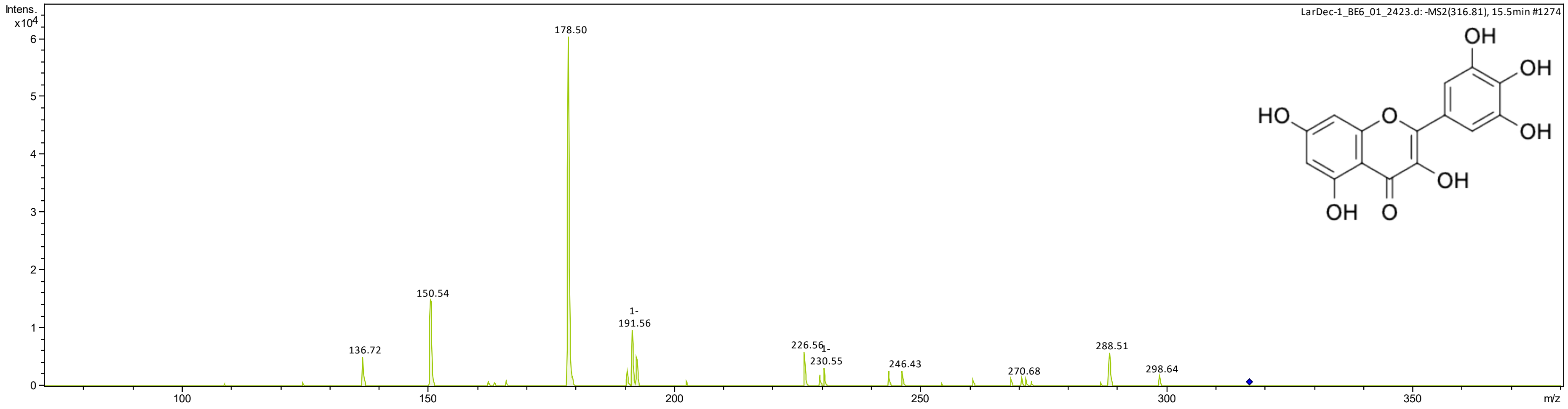

Figure S62. ESI-MS² spectrum (negative ion mode) of molecular ion at m/z 317 – compound (**25**).

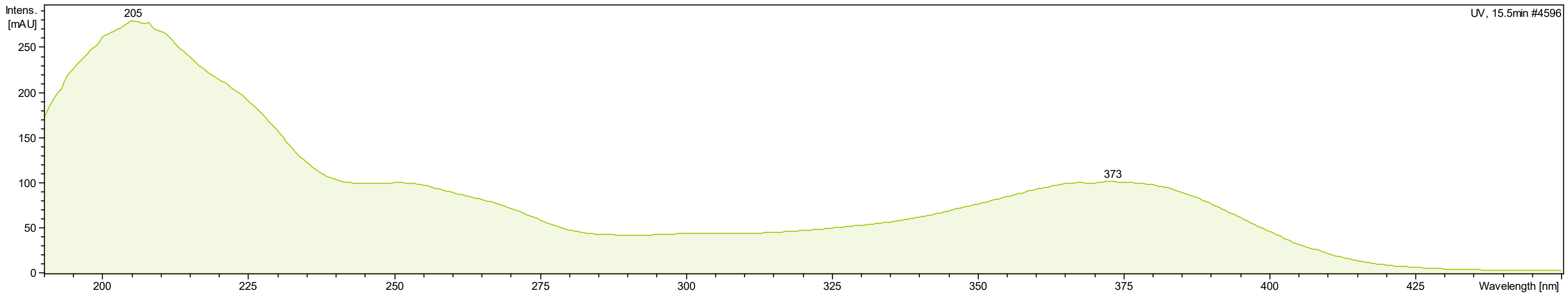

Figure S63. UV spectrum of compound (**25**).

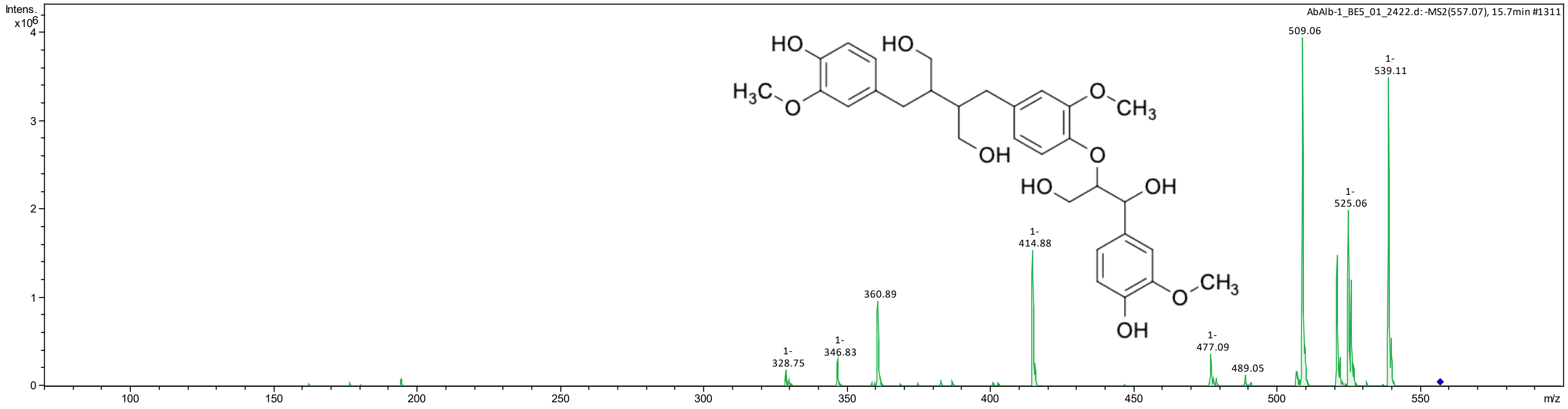

Figure S64. ESI-MS² spectrum (negative ion mode) of molecular ion at m/z 557 – compound (**26**).

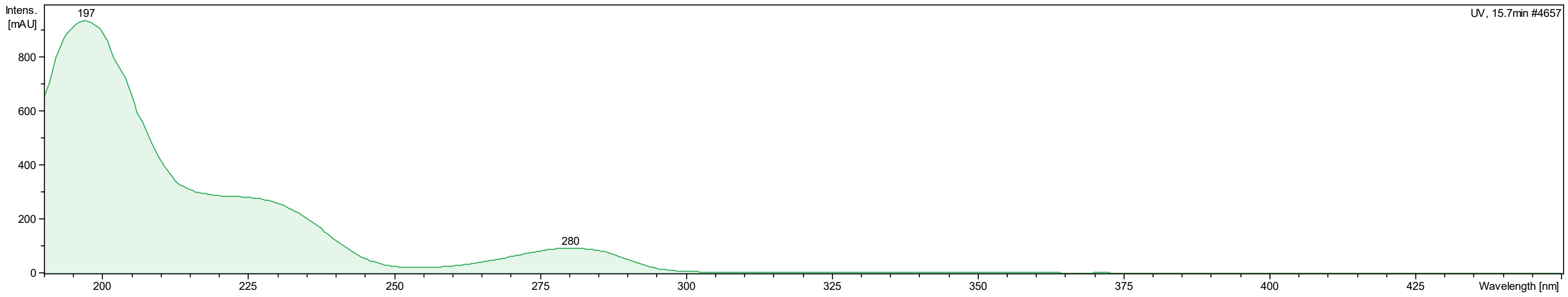

Figure S65. UV spectrum of compound (**26**).

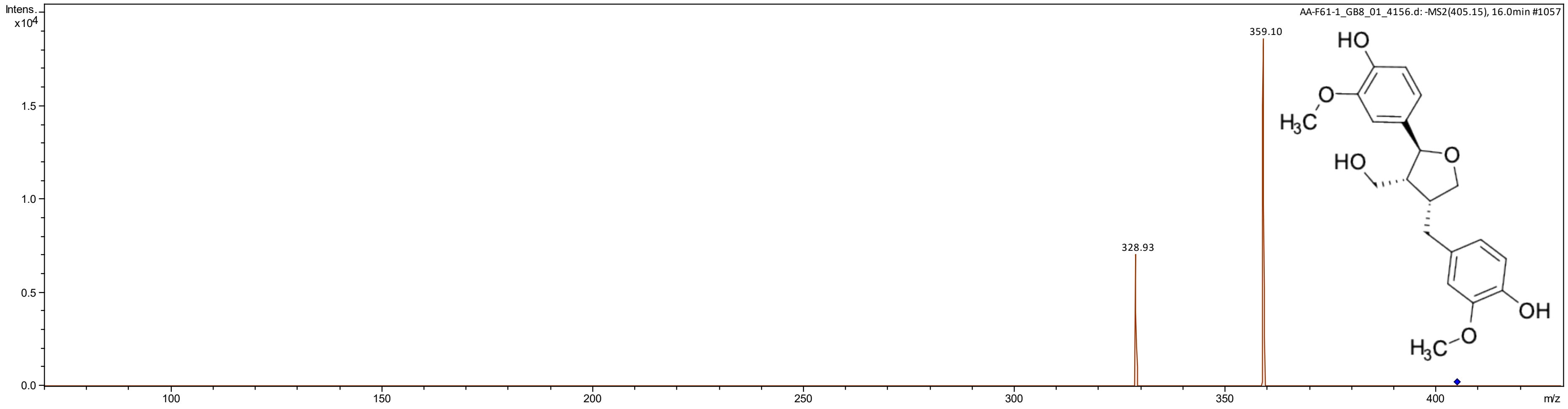

Figure S66. ESI-MS² spectrum (negative ion mode) of molecular ion at m/z 405 – compound (**27**).

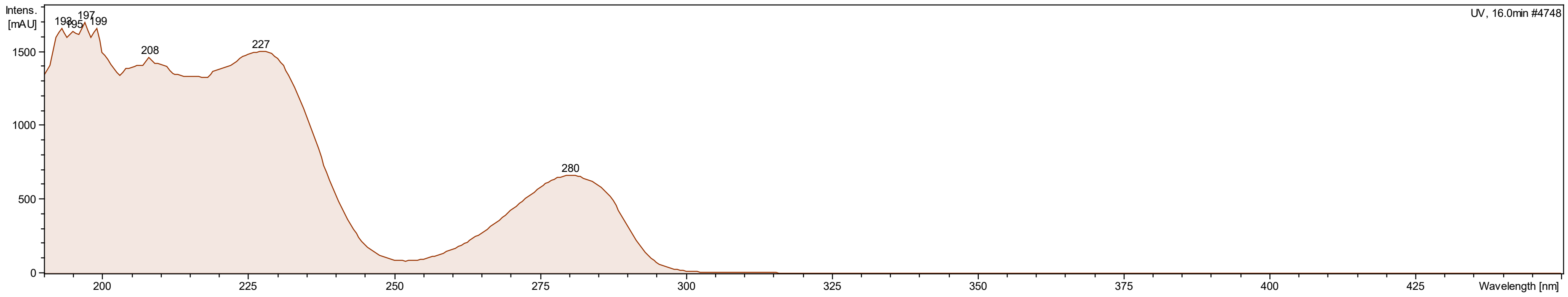

Figure S67. UV spectrum of compound (**27**).

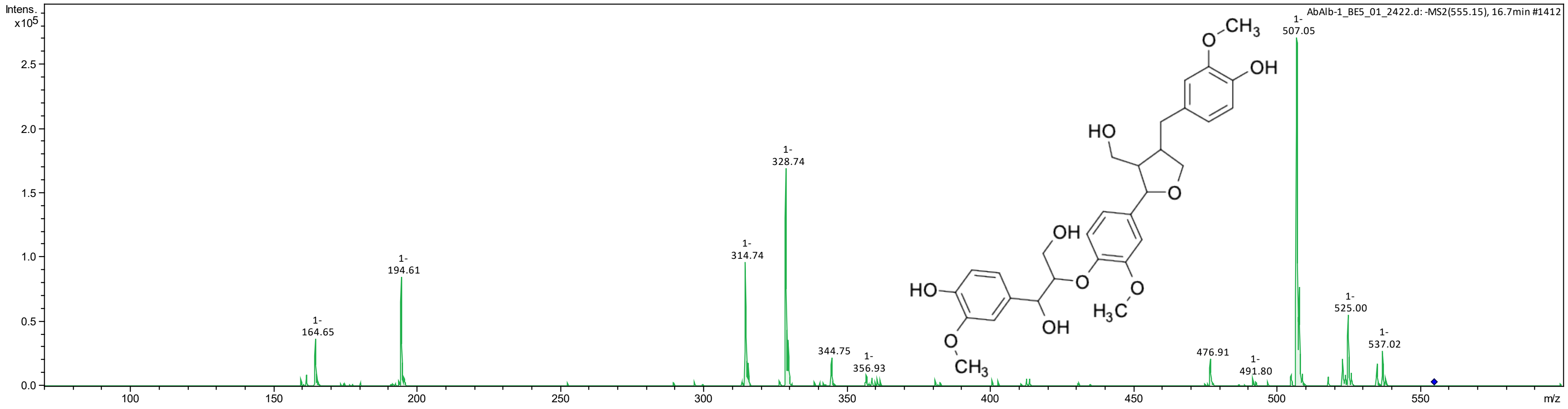

Figure S68. ESI-MS² spectrum (negative ion mode) of molecular ion at m/z 555 – compound (**28**).

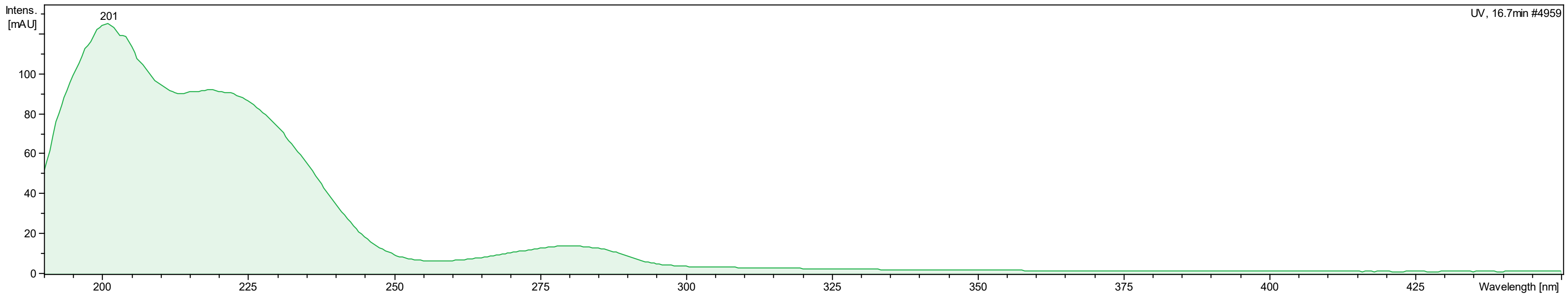

Figure S69. UV spectrum of compound (**28**).

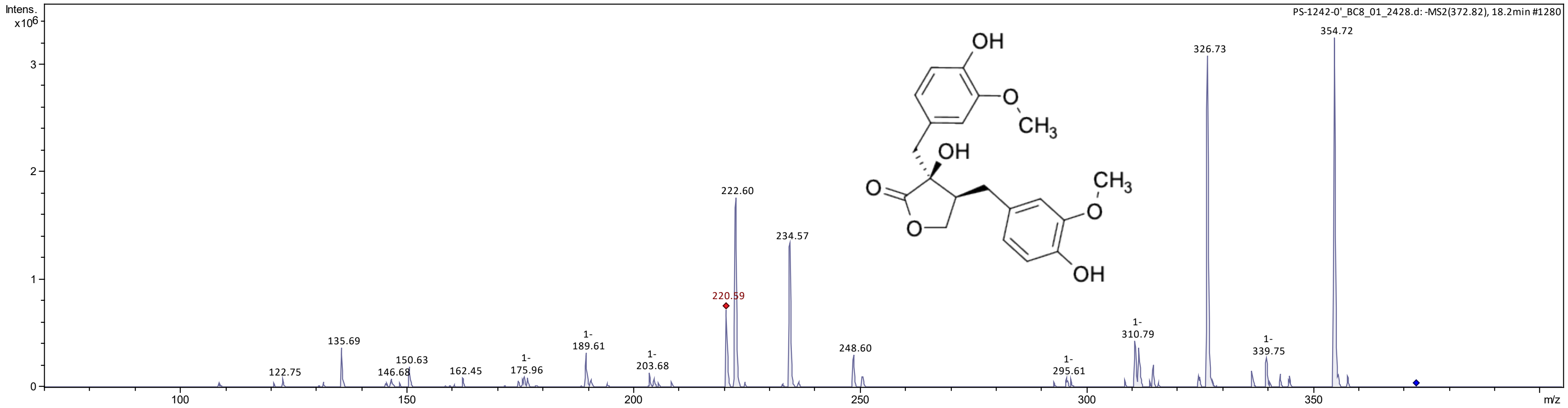

Figure S70. ESI-MS² spectrum (negative ion mode) of molecular ion at m/z 373 – compound (**29**).

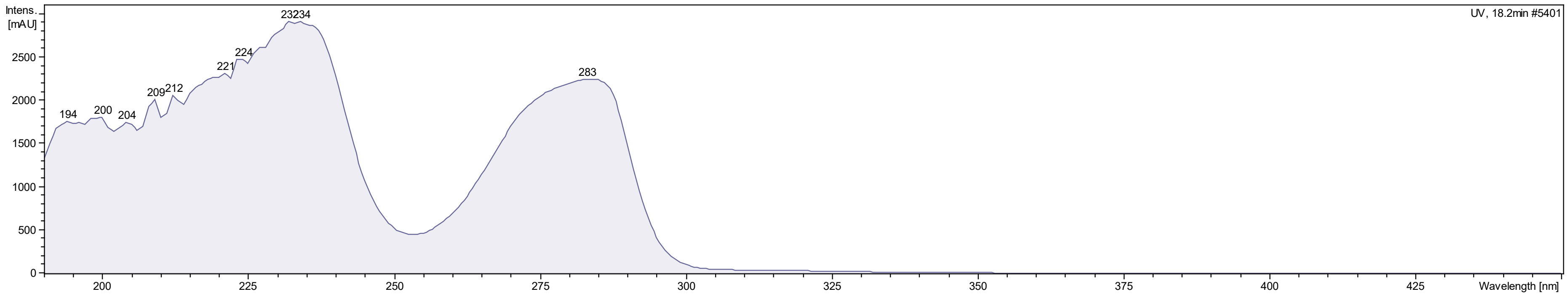

Figure S71. UV spectrum of compound (**29**).

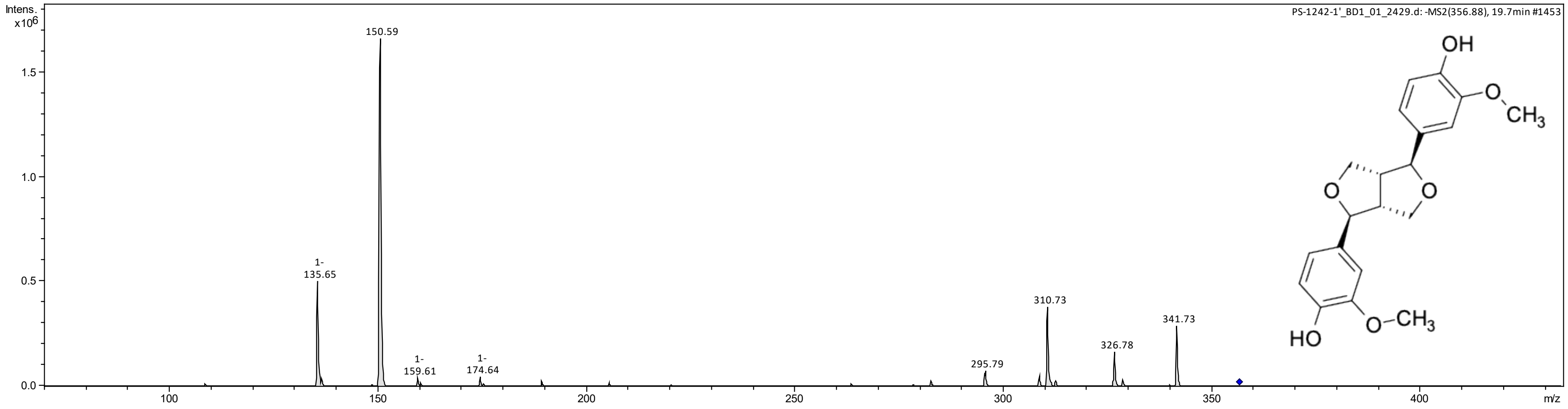

Figure S72. ESI-MS² spectrum (negative ion mode) of molecular ion at m/z 357 – compound (**30**).

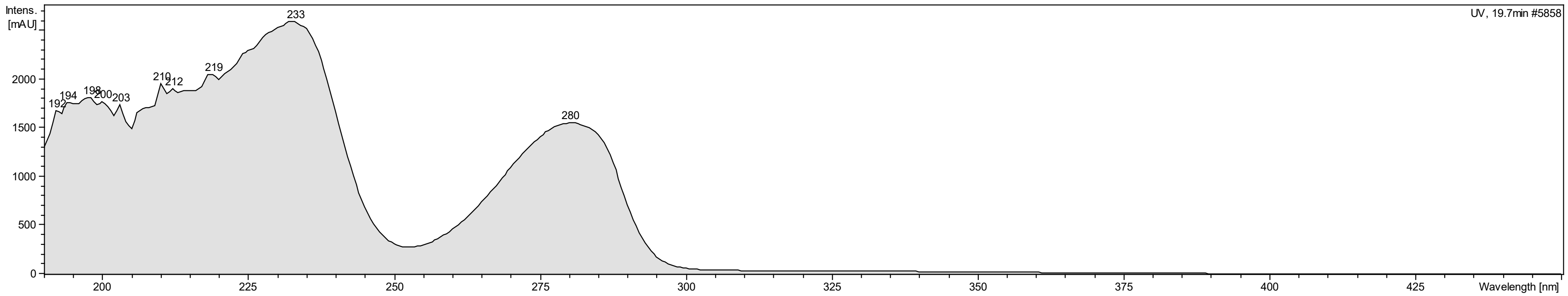

Figure S73. UV spectrum of compound (**30**).

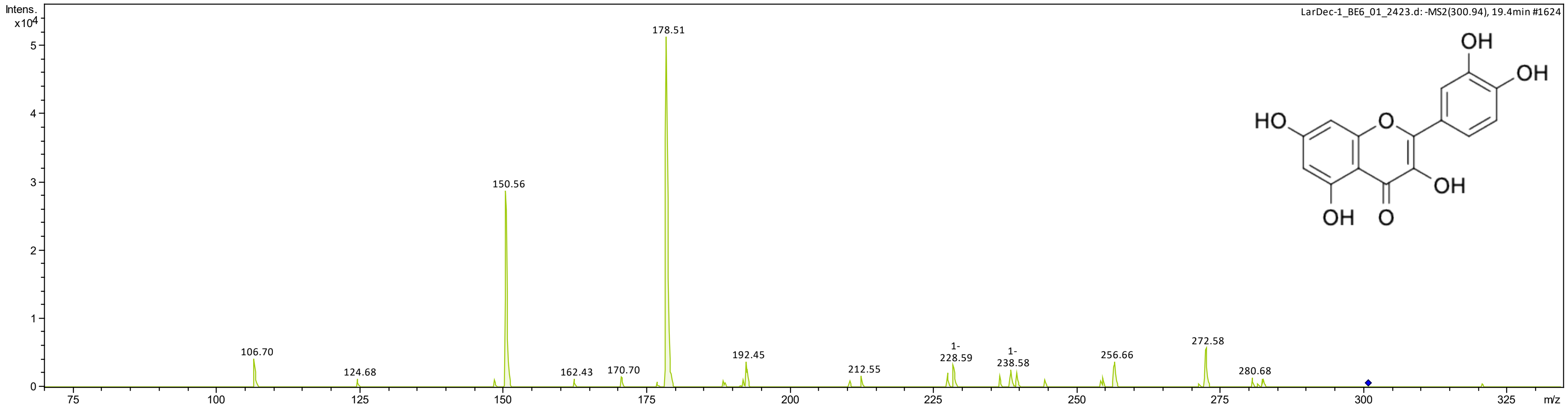

Figure S74. ESI-MS² spectrum (negative ion mode) of molecular ion at m/z 301 – compound (**31**).

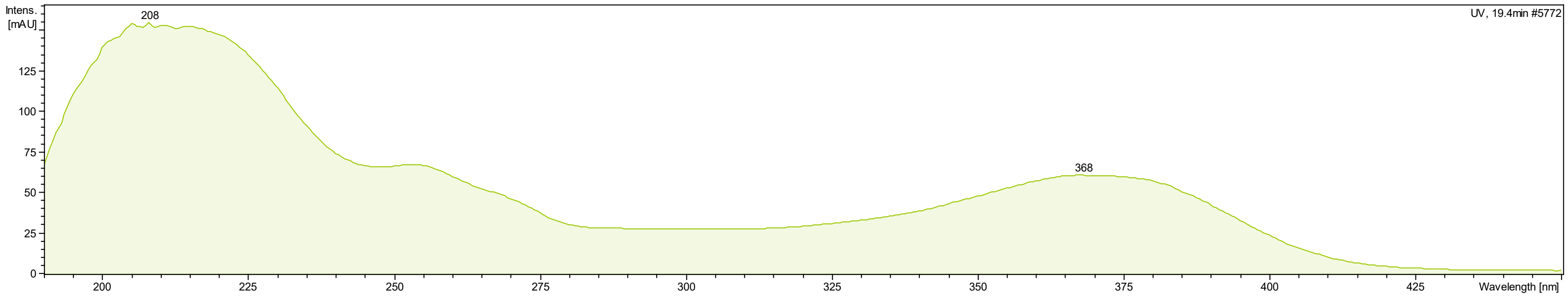

Figure S75. UV spectrum of compound (**31**).

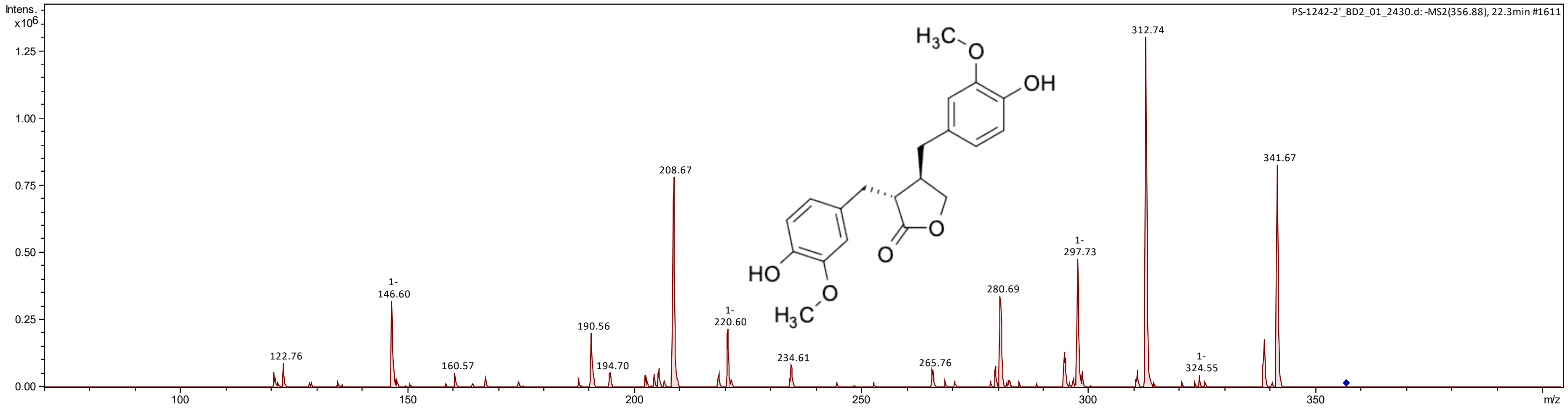

Figure S76. ESI-MS² spectrum (negative ion mode) of molecular ion at m/z 357 – compound (**32**).

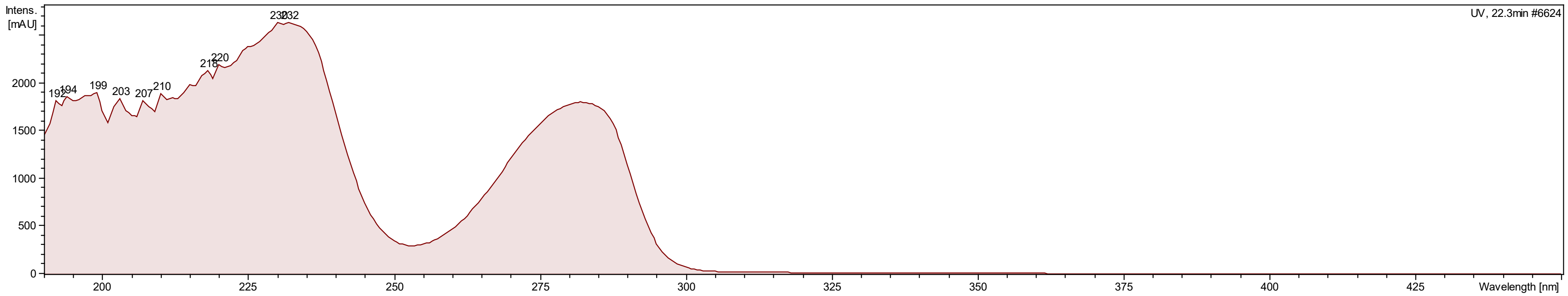

Figure S77. UV spectrum of compound (**32**).

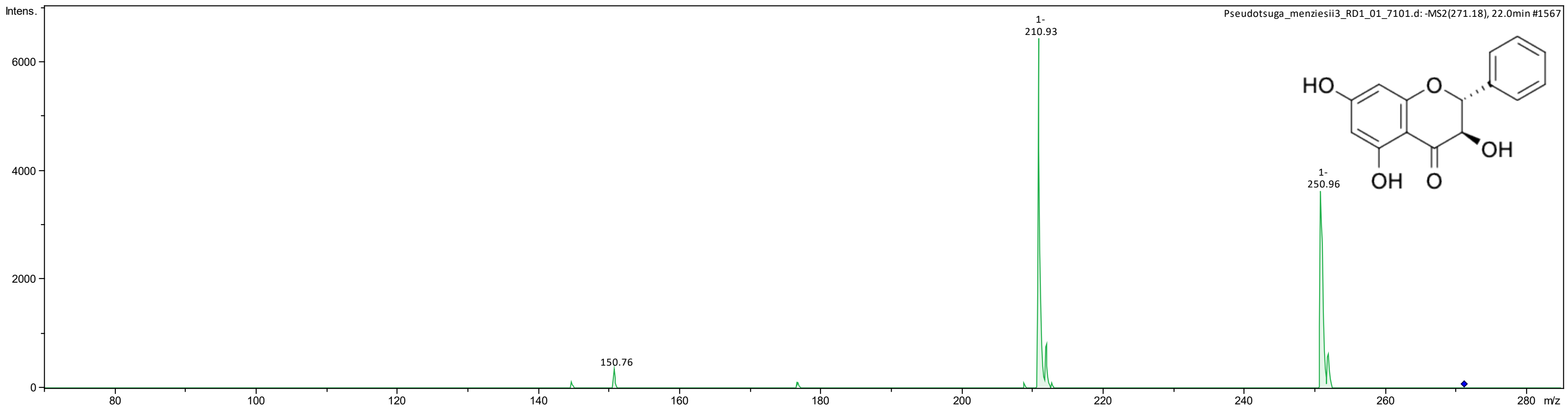

Figure S78. ESI-MS² spectrum (negative ion mode) of molecular ion at m/z 271 – compound (**33**).

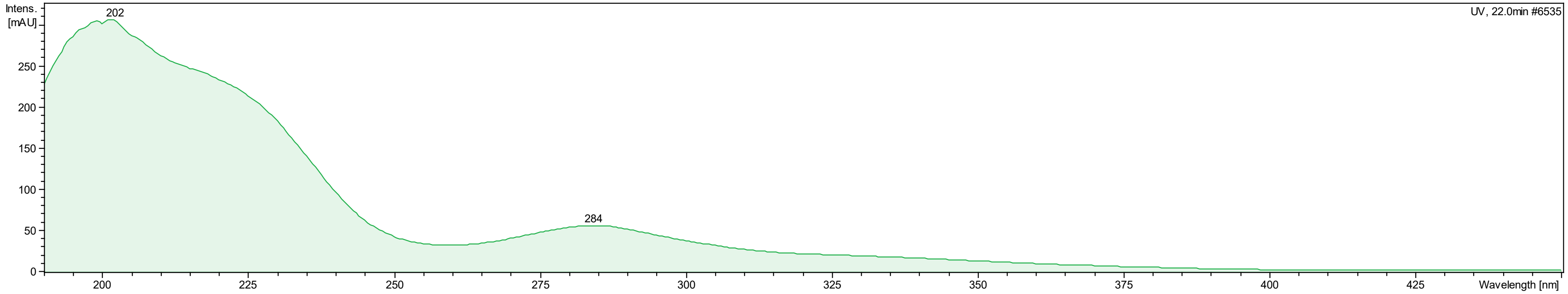

Figure S79. UV spectrum of compound (**33**).

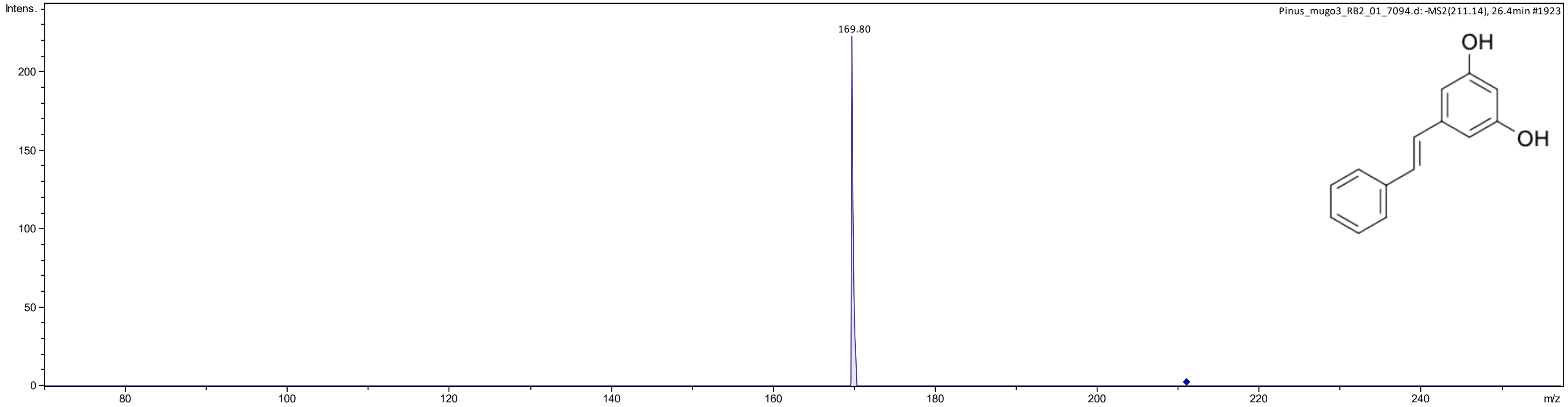

Figure S80. ESI-MS² spectrum (negative ion mode) of molecular ion at m/z 211 – compound (**34**).

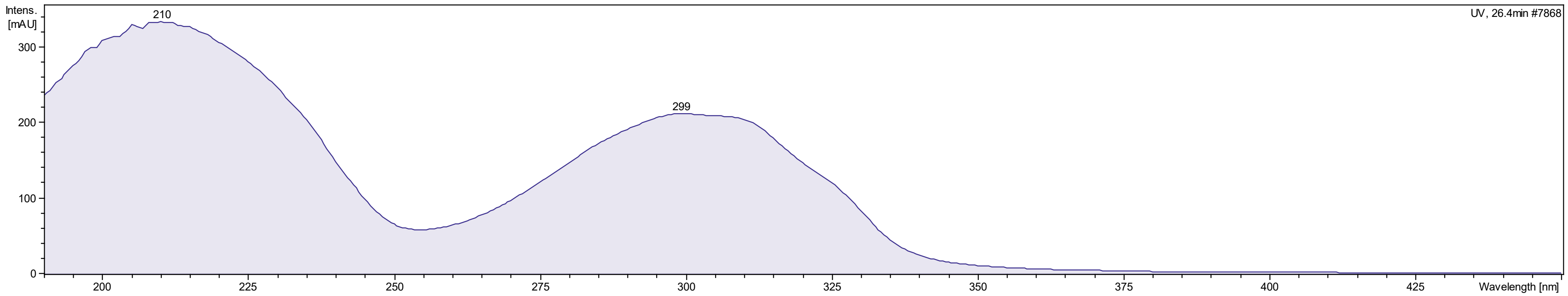

Figure S81. UV spectrum of compound (**34**).

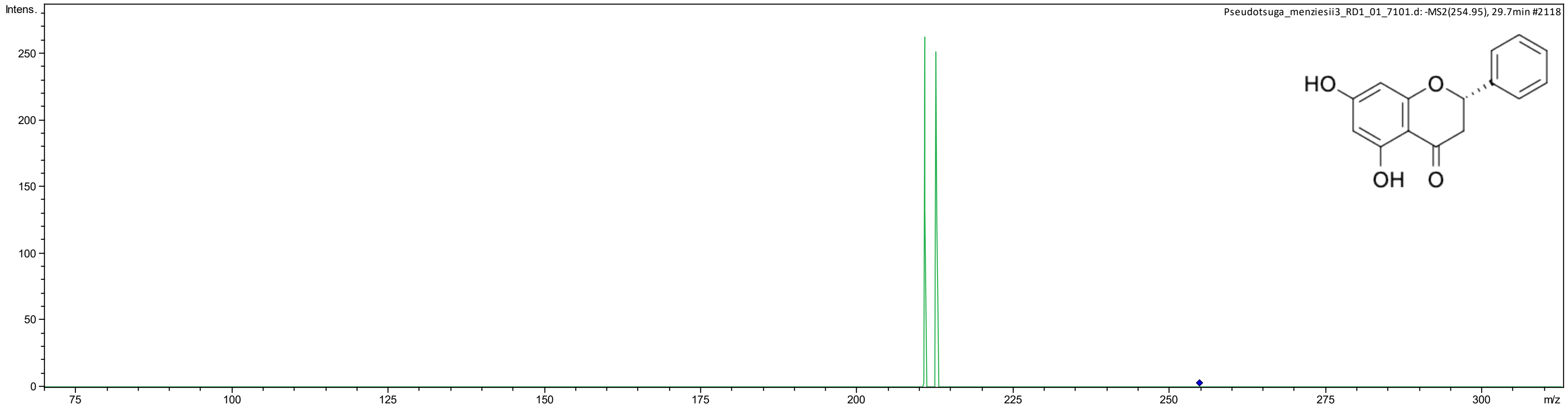

Figure S82. ESI-MS^2 spectrum (negative ion mode) of molecular ion at m/z 255 – compound (**35**).

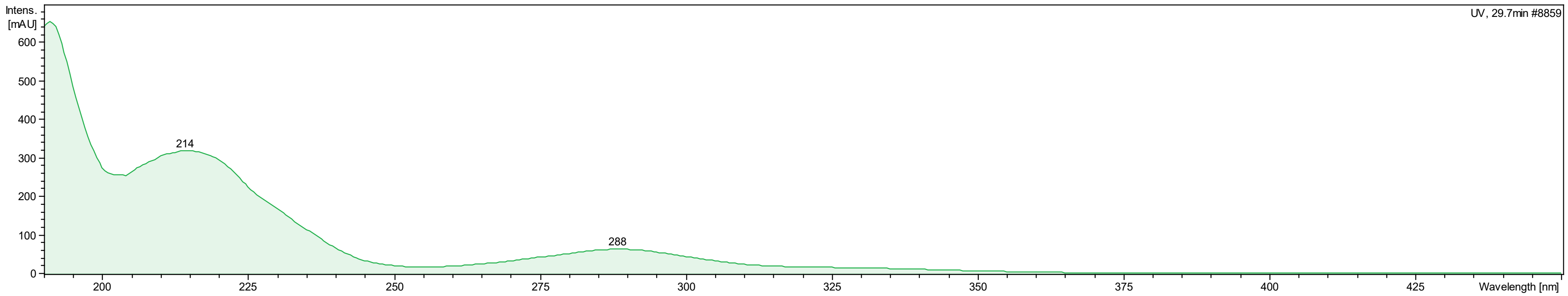

Figure S83. UV spectrum of compound (**35**).

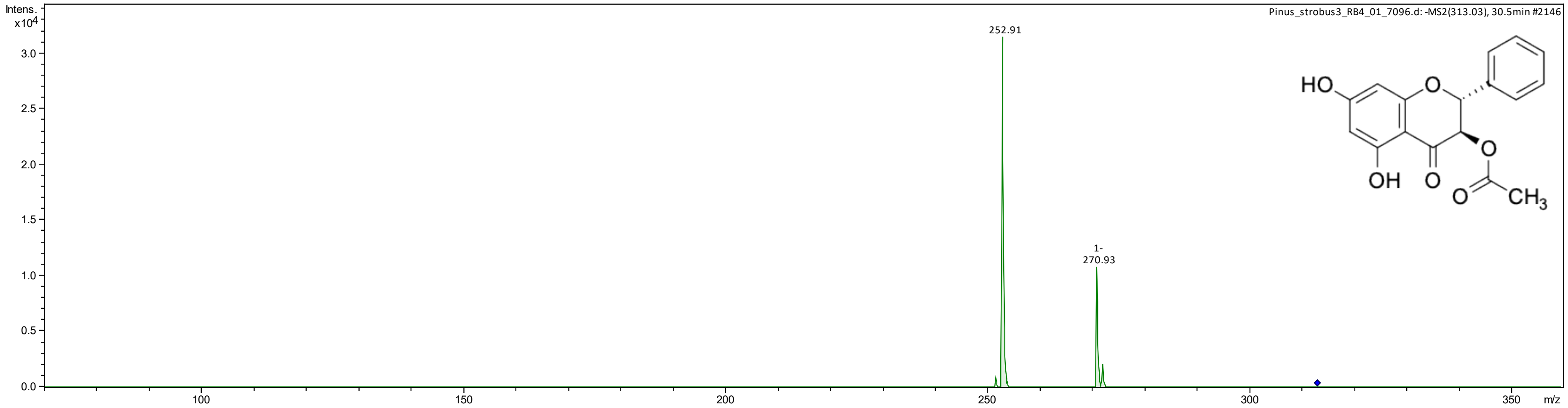

Figure S84. ESI-MS² spectrum (negative ion mode) of molecular ion at m/z 313 – compound (**36**).

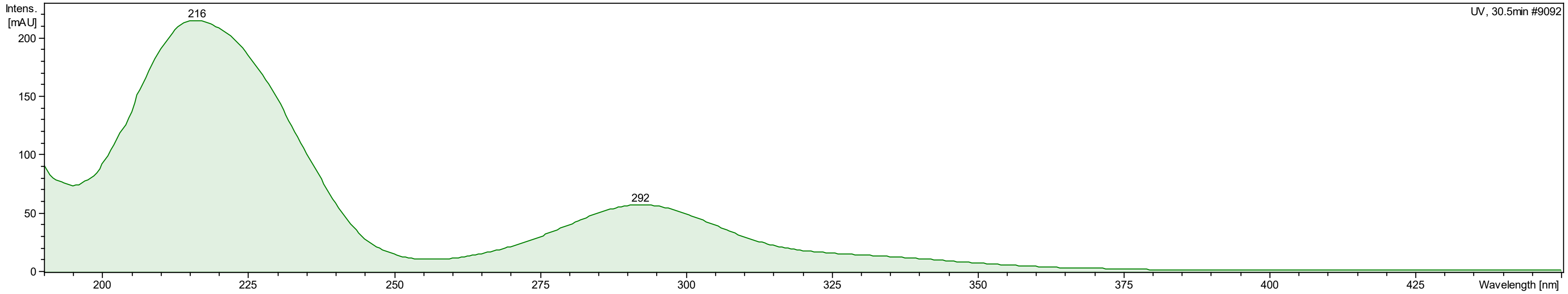

Figure S85. UV spectrum of compound (**36**).

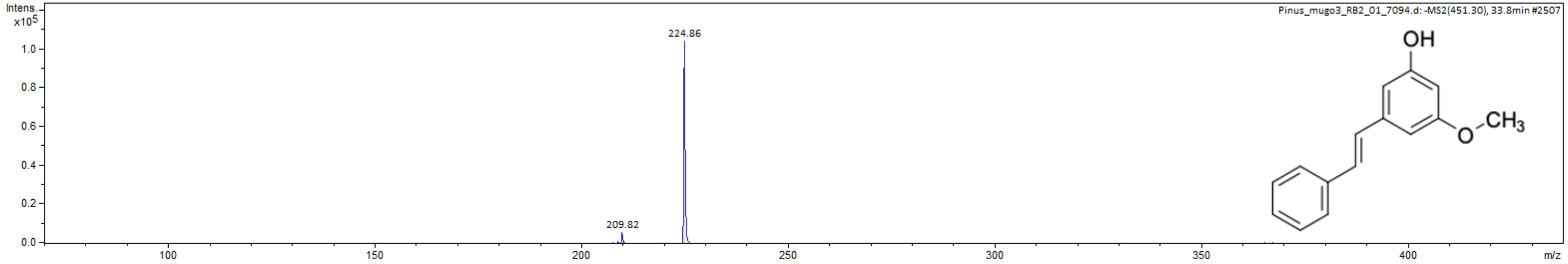

Figure S86. ESI-MS² spectrum (positive ion mode) of molecular ion at m/z 227 – compound (**37**).

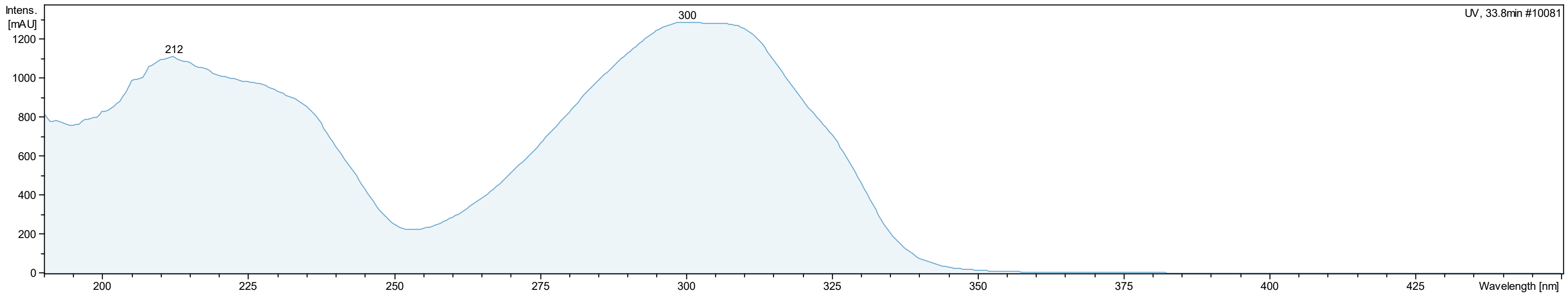

Figure S87. UV spectrum of compound (**37**).

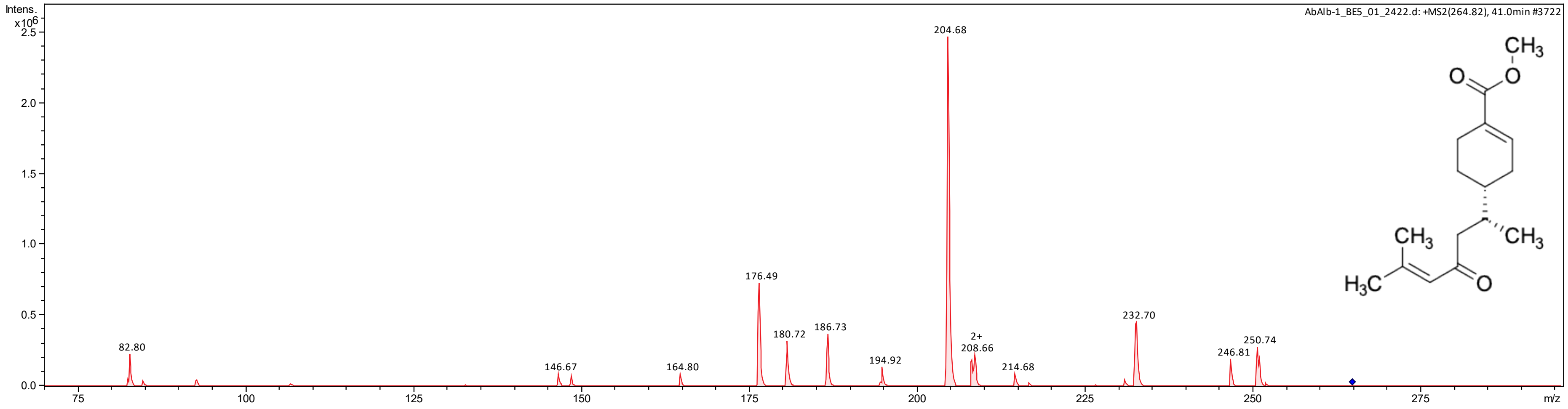

Figure S88. ESI-MS² spectrum (positive ion mode) of molecular ion at m/z 265 – compound (**38**).

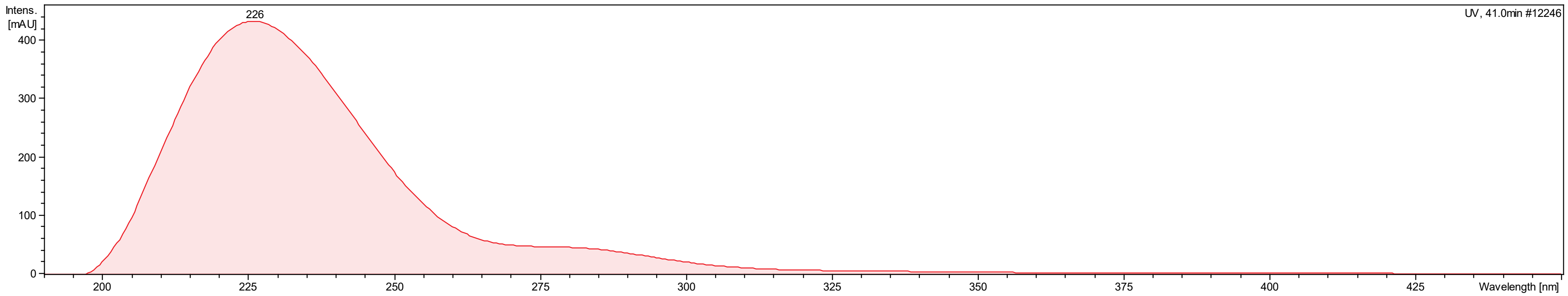

Figure S89. UV spectrum of compound (**38**).

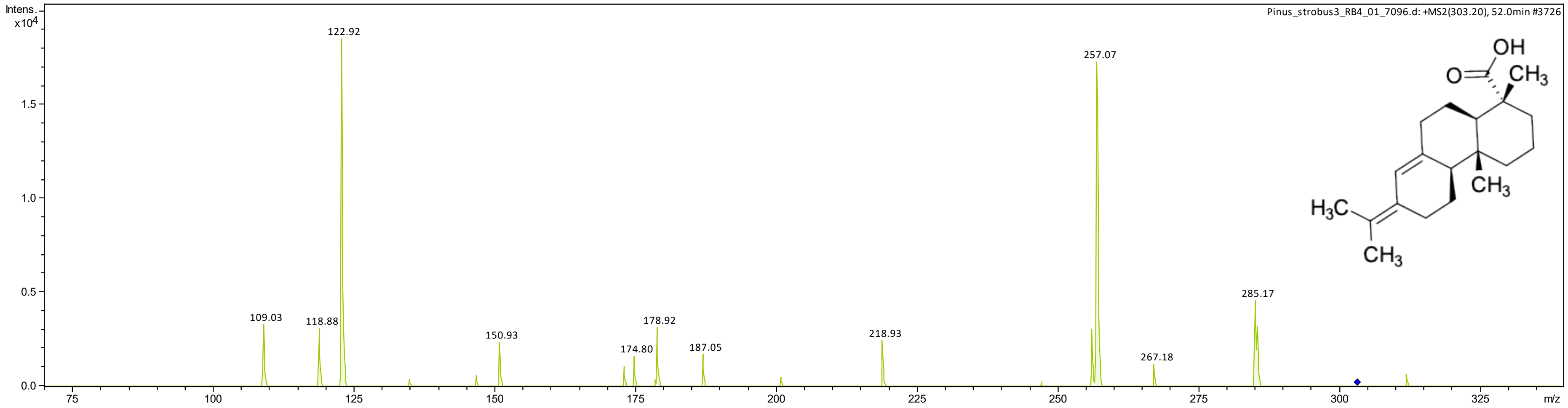

Figure S90. ESI-MS² spectrum (positive ion mode) of molecular ion at m/z 303 – compound (**39**).

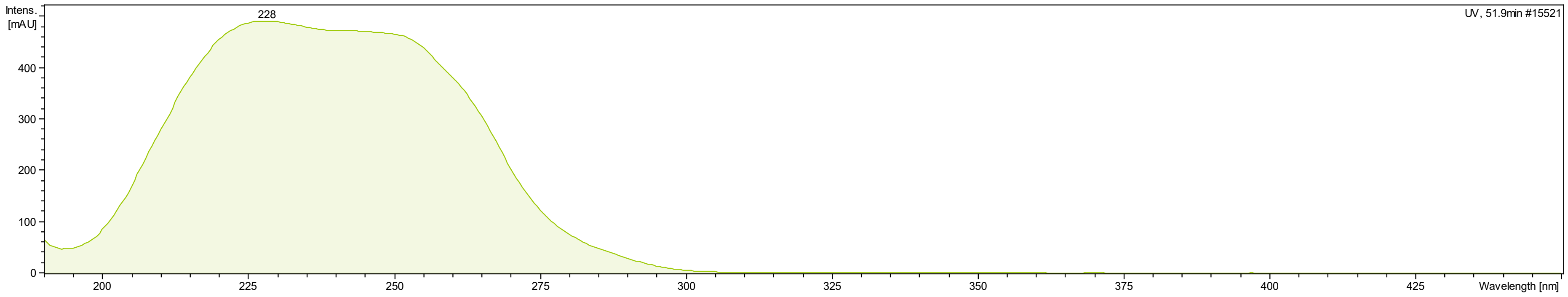

Figure S91. UV spectrum of compound (**39**).

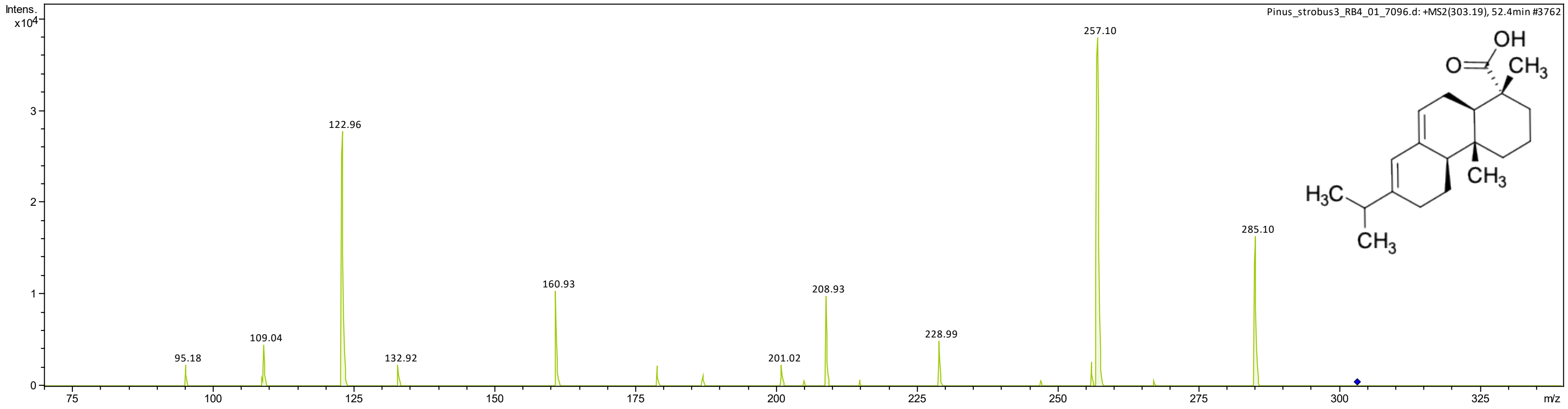

Figure S92. ESI-MS² spectrum (positive ion mode) of molecular ion at m/z 303 – compound (**40**).

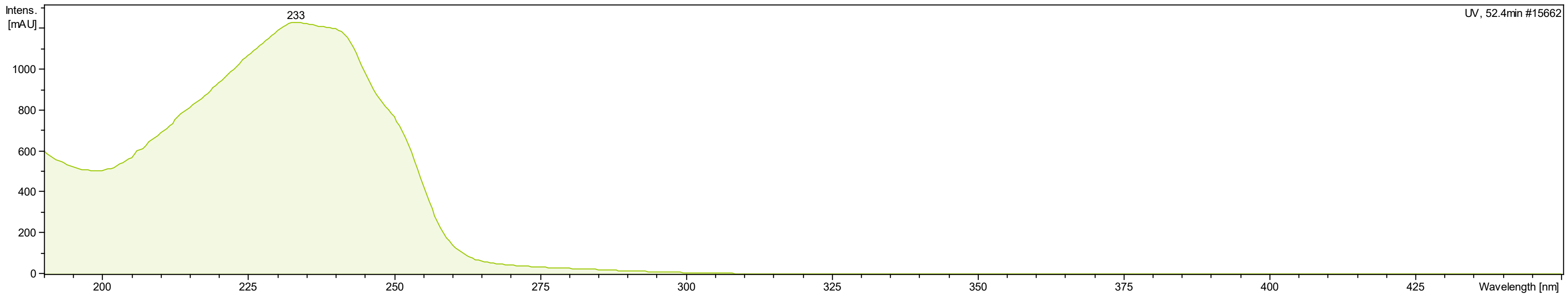

Figure S93. UV spectrum of compound (**40**).

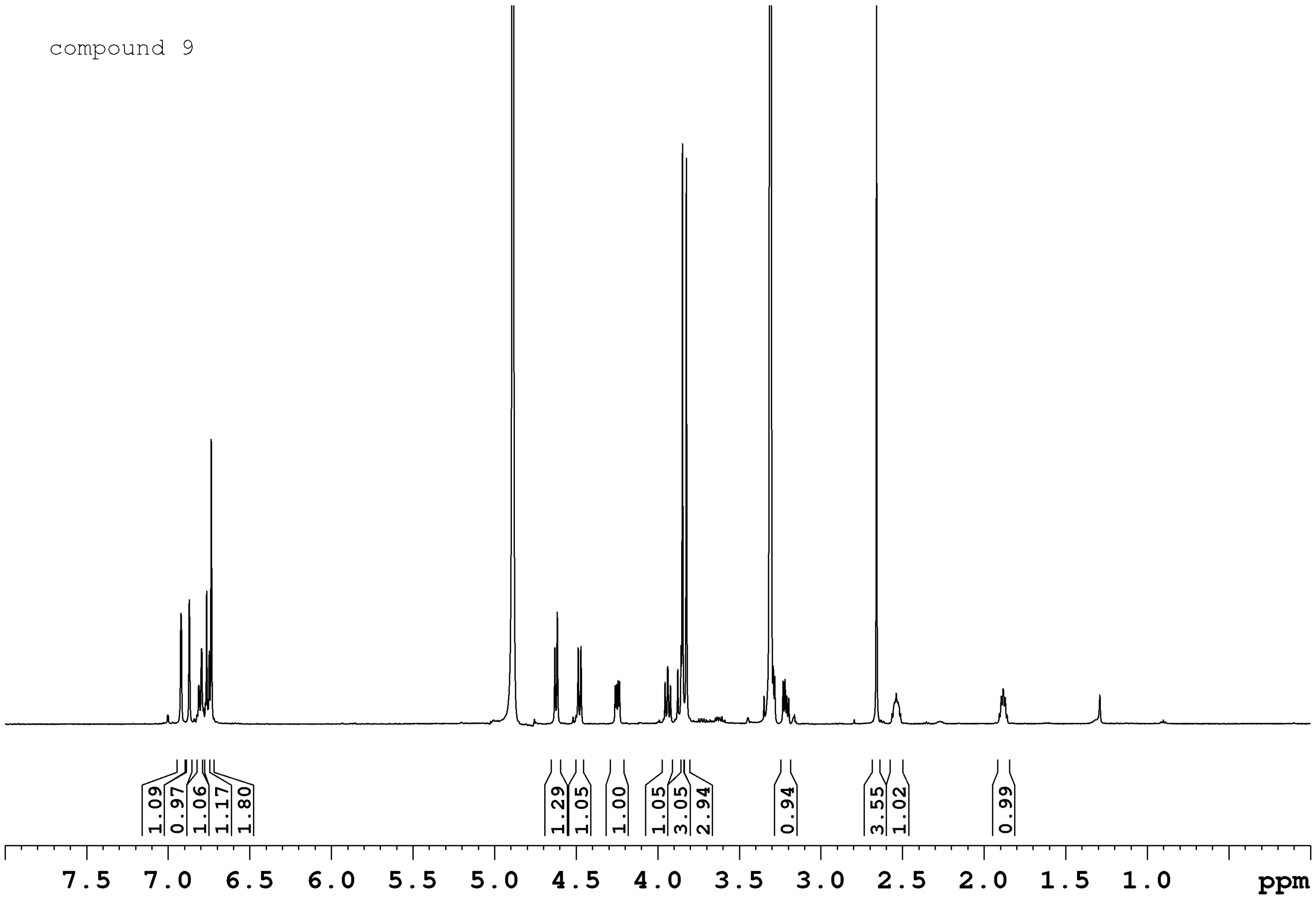

Figure S94. ^{1}H NMR spectrum of compound (**9**).

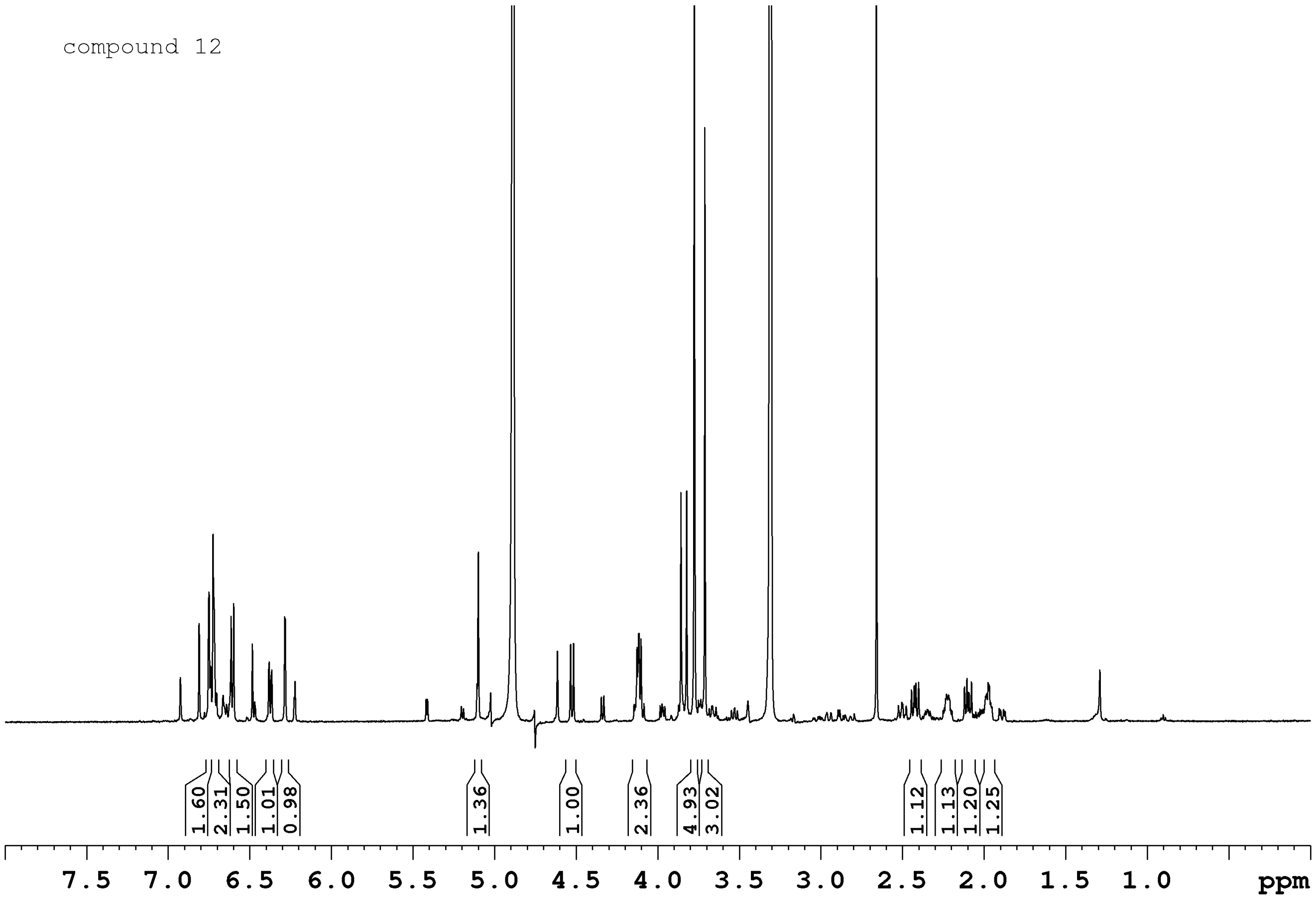

Figure S95. ^{1}H NMR spectrum of compound (**12**).

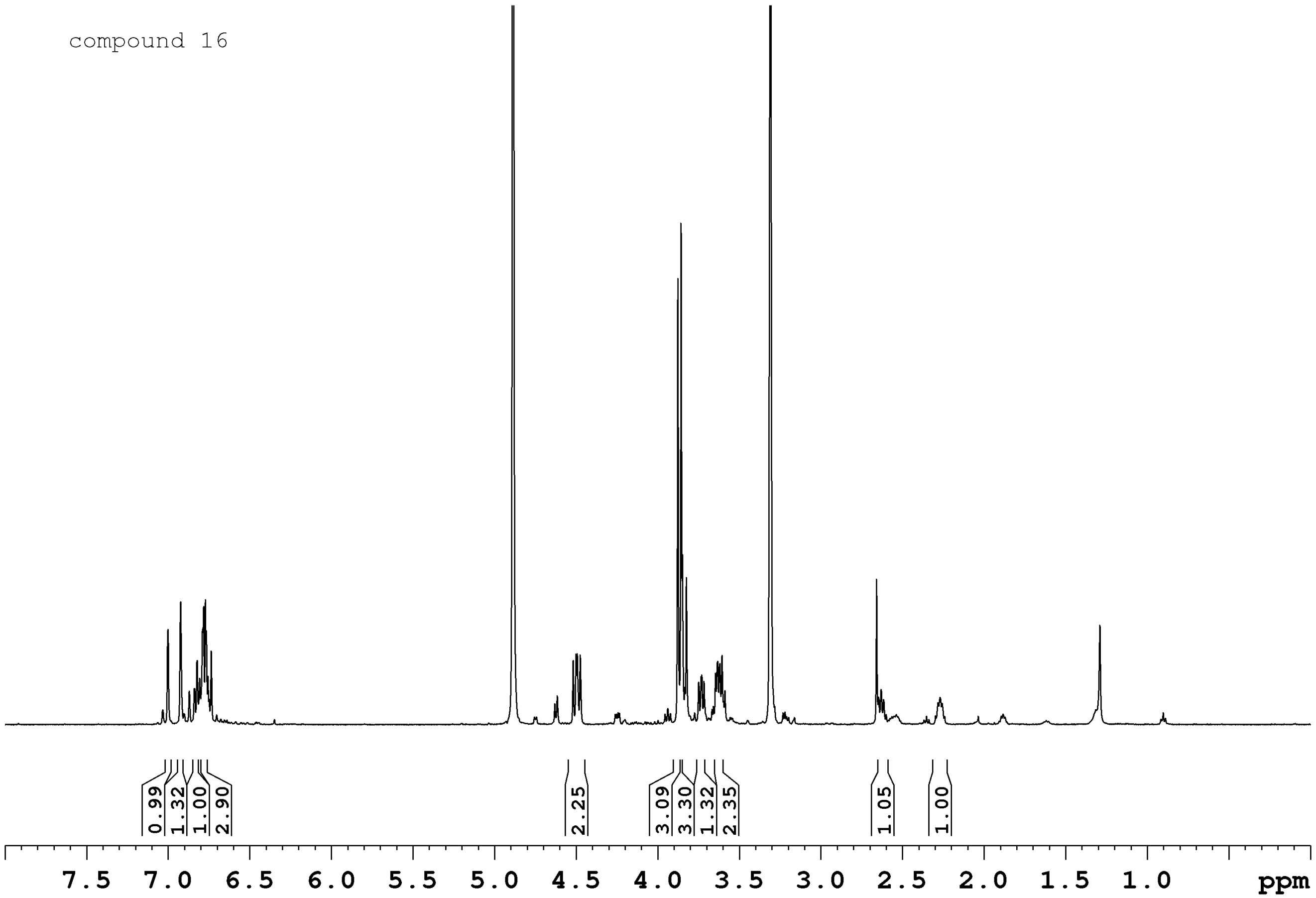

Figure S96. ^{1}H NMR spectrum of compound (**16**).

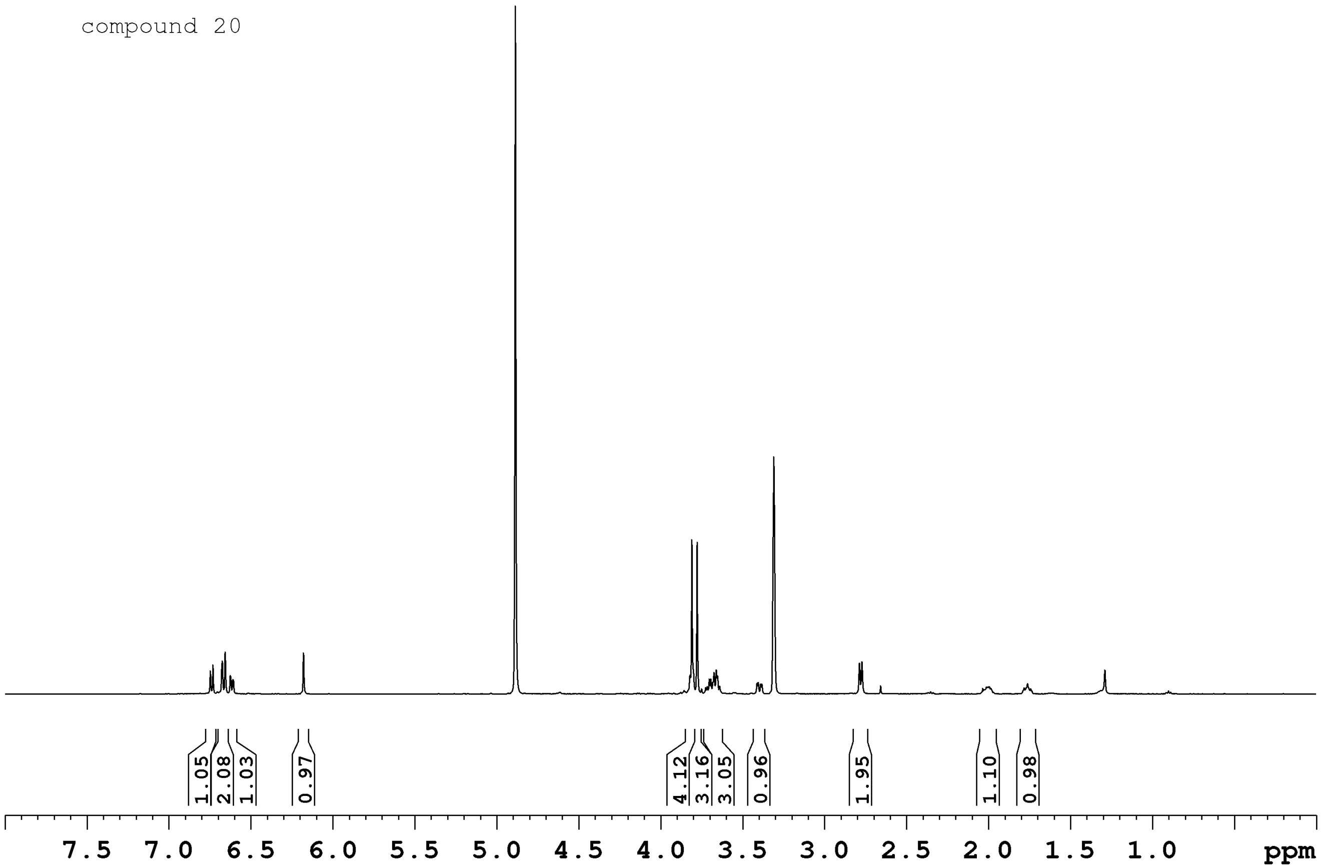

Figure S97. ^{1}H NMR spectrum of compound (**20**).

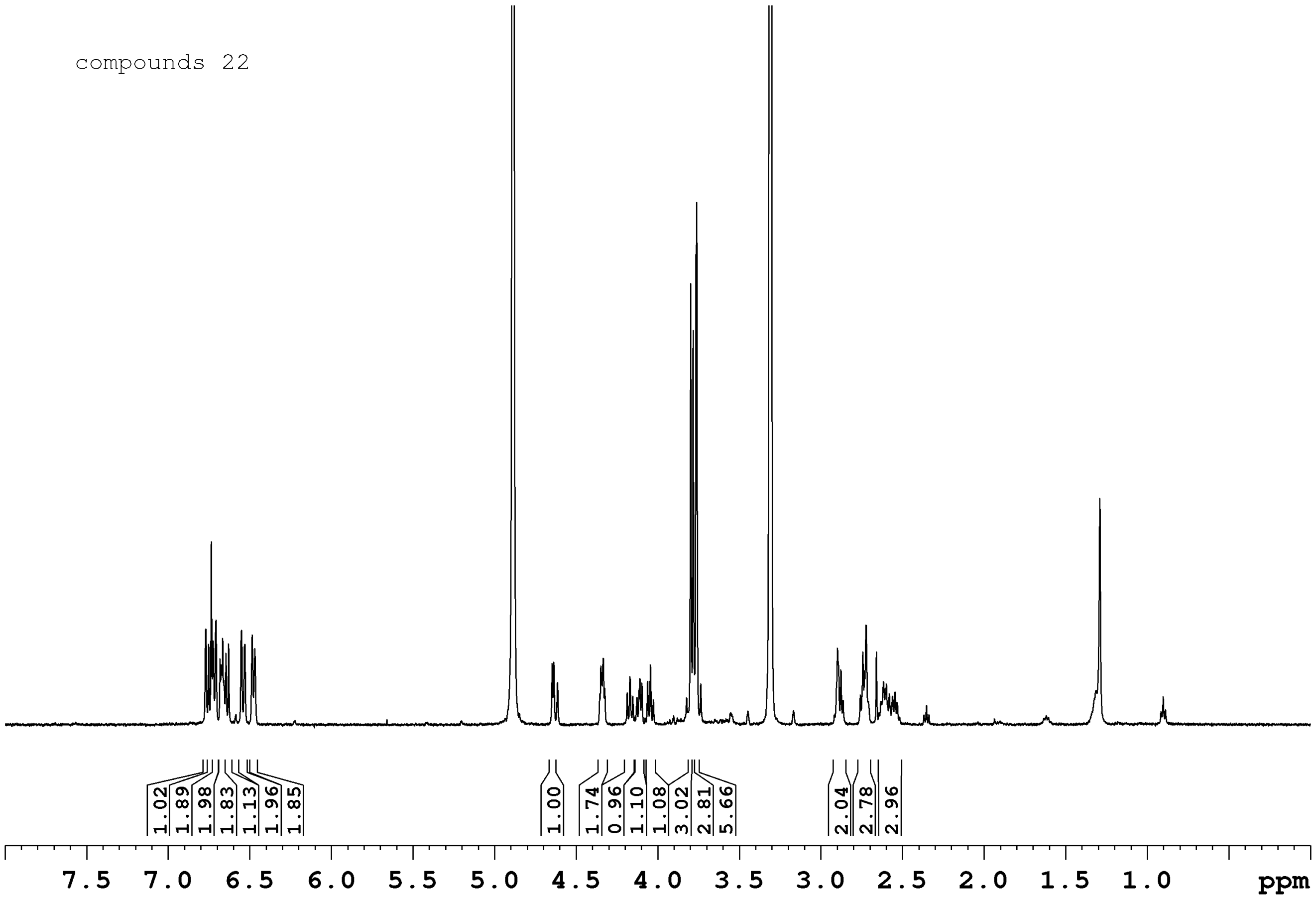

Figure S98. ^{1}H NMR spectrum of compound (**22**).

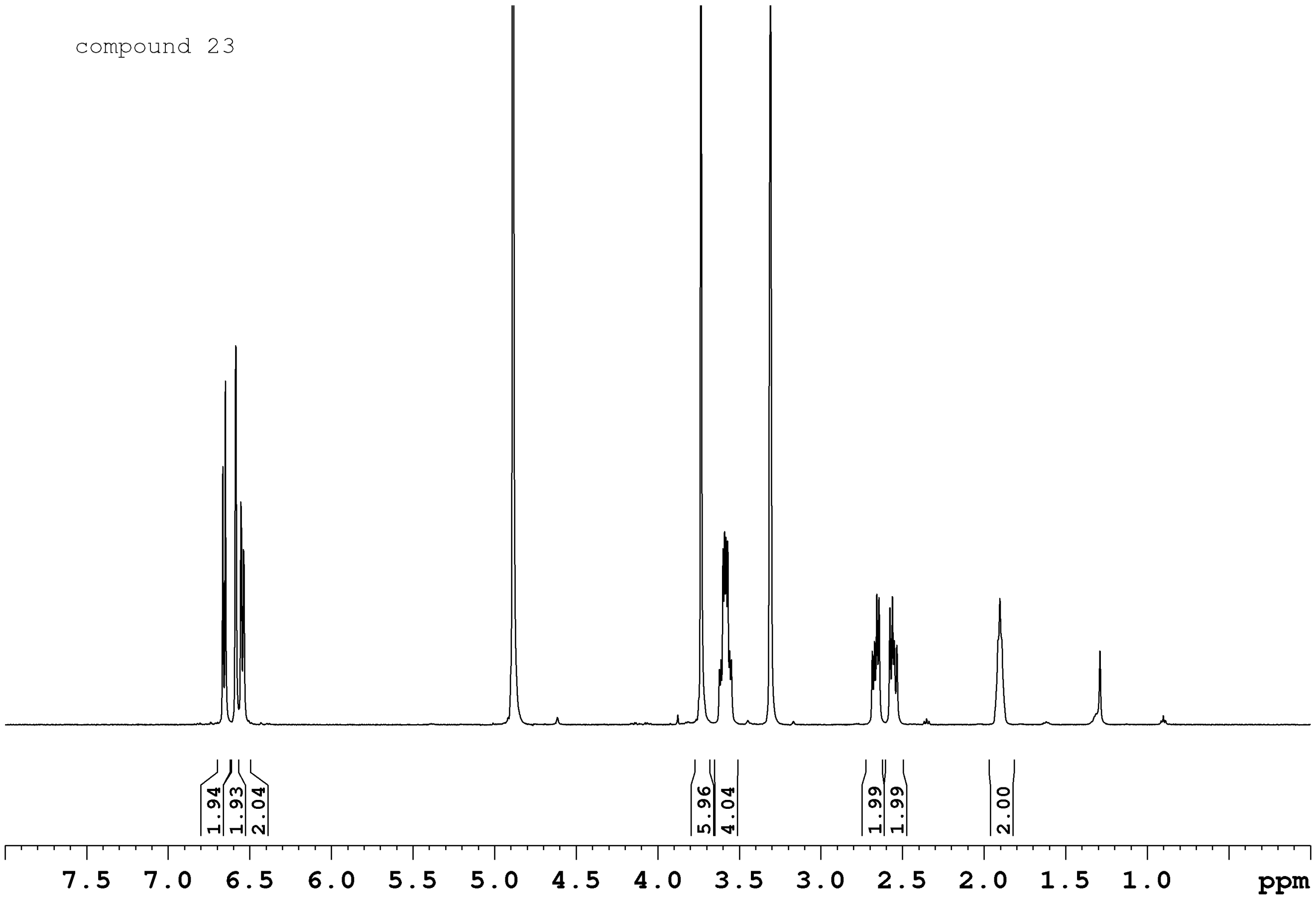

Figure S99. ^{1}H NMR spectrum of compound (**23**).

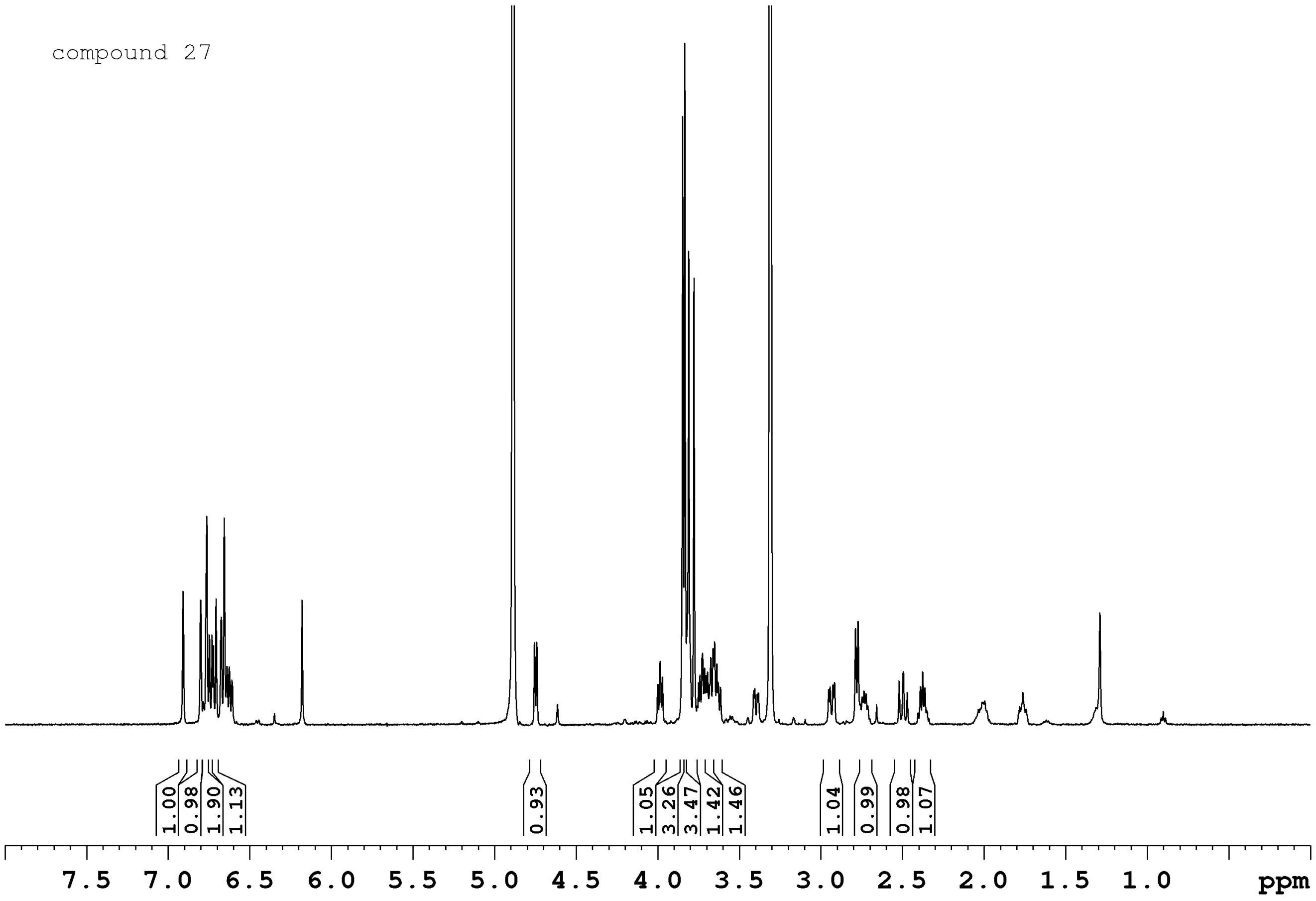

Figure S100. ^{1}H NMR spectrum of compound (**27**).

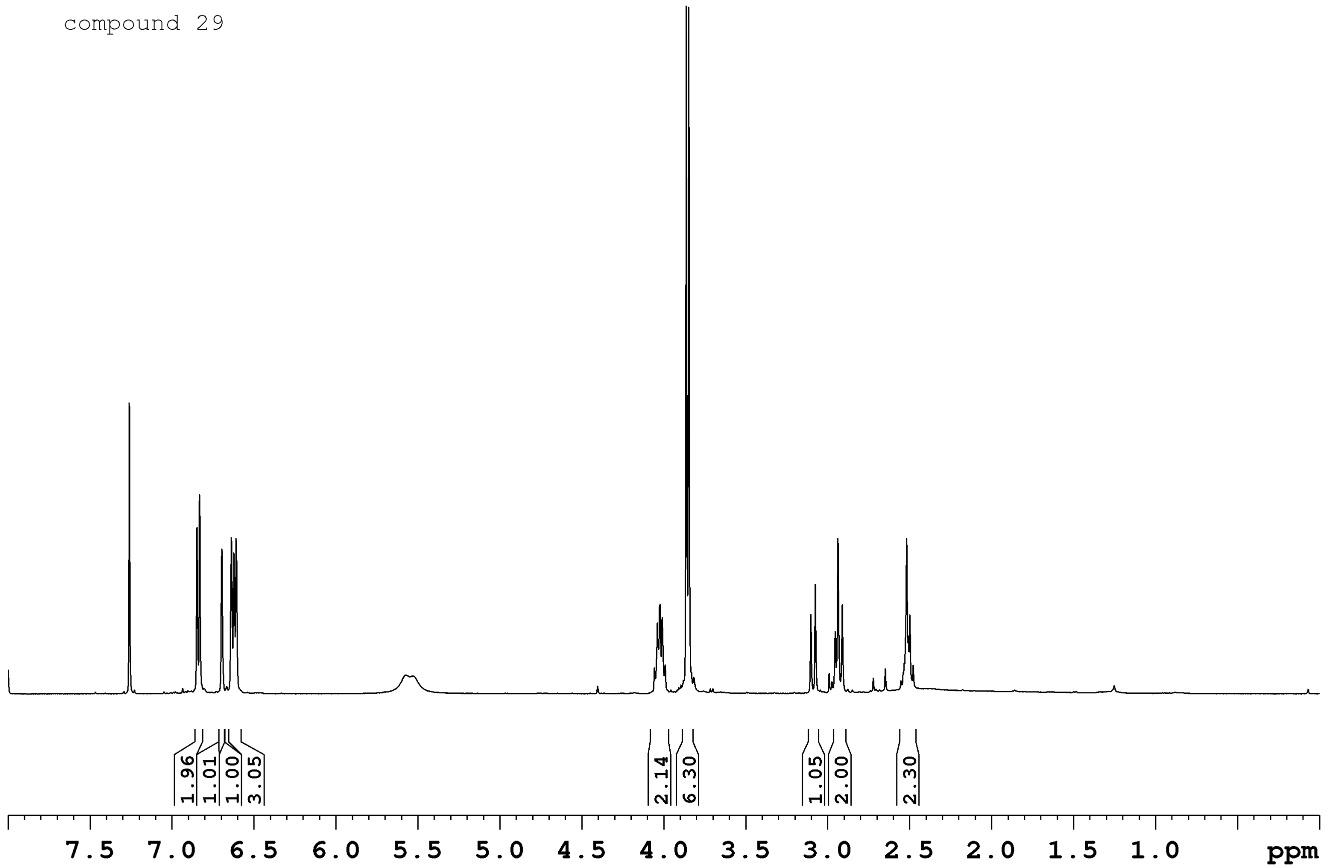

Figure S101. ^{1}H NMR spectrum of compound (**29**).

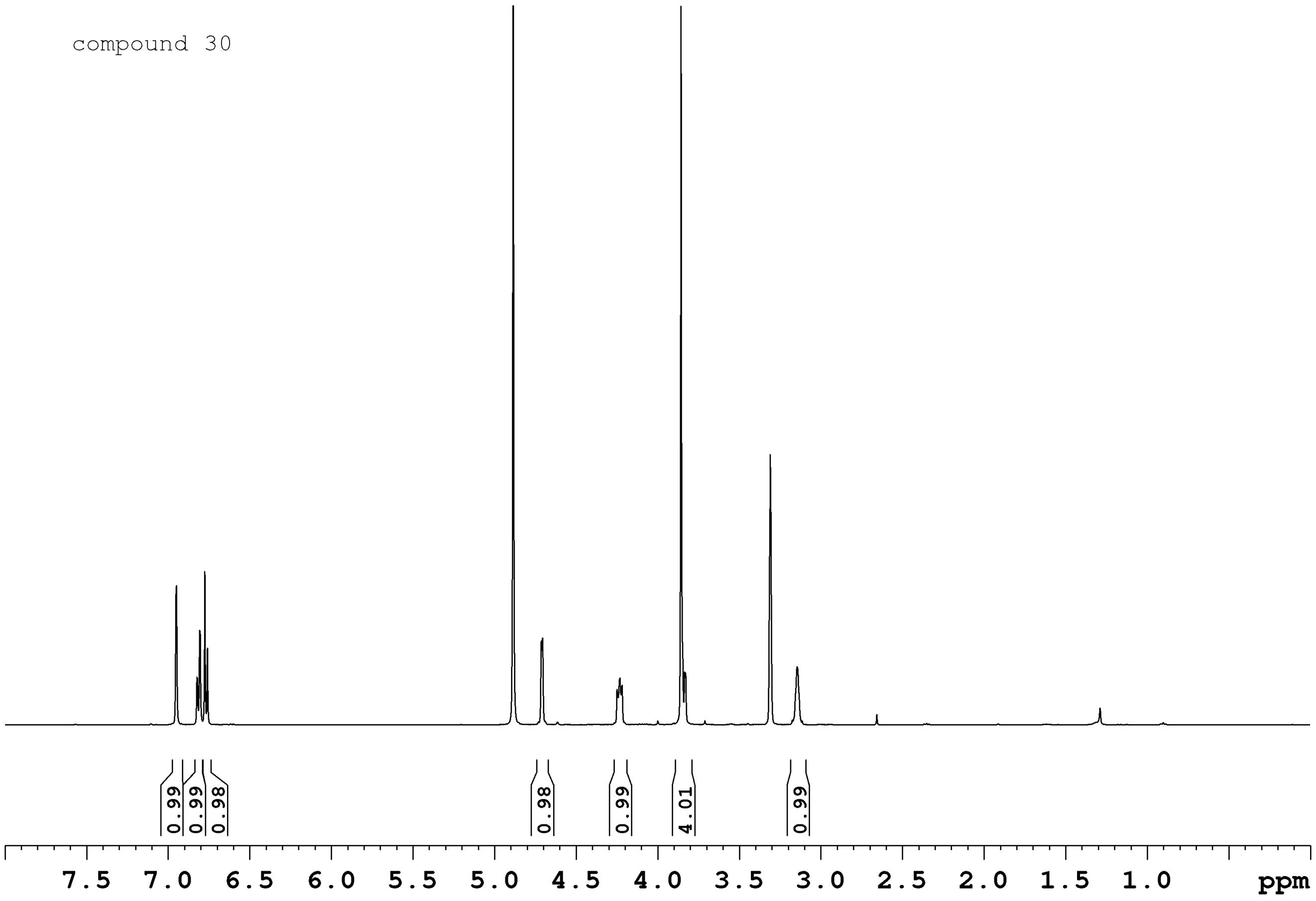

Figure S102. ^1H NMR spectrum of compound (**30**).

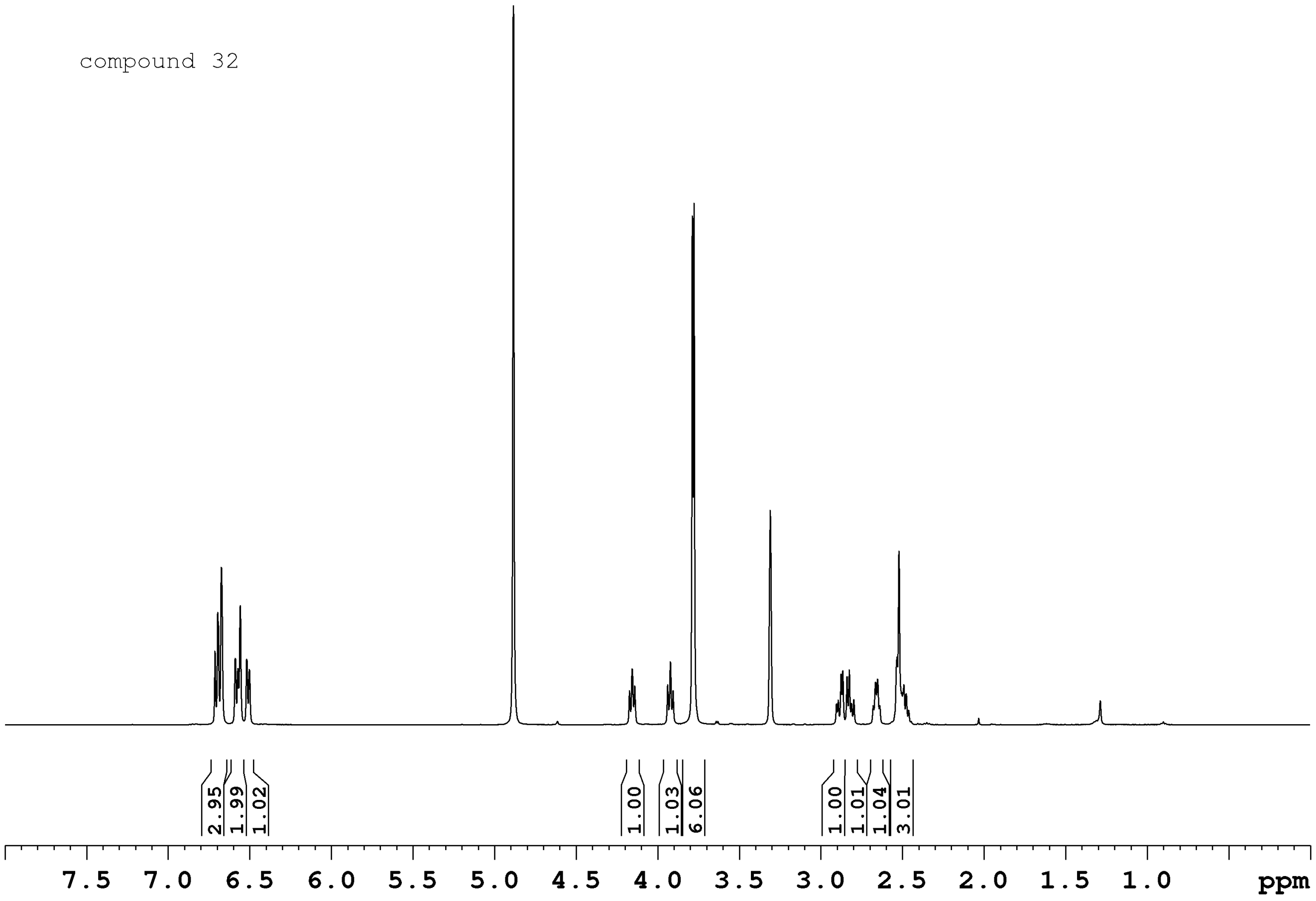

Figure S103. ^{1}H NMR spectrum of compound (**32**).

Table S1. ^{1}H chemical shifts in ppm for compounds **9**, **12**, **16**, **20**, **22**, **23**, **27**, **30** and **32** in methanol-d4 and **29** in chloroform-d (500 MHz, 295 K).

atom	**9**	**12**	**16**	**20**	**22***	**23**	**27**	**29**	**30**	**32**
H2	6.92	6.29	6.92	6.18	6.53; 6.55	6.59	6.91	6.70	6.95	6.67
H5	6.76	6.61	6.78	6.67	6.64; 6.67	6.66	6.77	6.84	6.76	6.71
H6	6.81	6.37	6.80		6.48; 6.48	6.55	6.77	6.63	6.81	6.58
H7a	4.62	2.10	4.51	2.78	2.59; 2.73	2.56	4.75	2.92	4.71	2.82
H7b	-	2.41	-	2.78	2.73; 2.88	2.67	-	3.09	-	2.89
H8	1.88	1.99	2.27	2.00	2.73; 2.90	1.90	2.38	-	3.15	2.66
H9a	3.21	5.10	3.61	3.67	-	3.56	3.63	-	3.84	-
H9b	3.30	-	3.73	3.67	-	3.61	3.83	-	4.23	-
OMe	3.85	3.72	3.86	3.81	3.76; 3.77	3.74	3.85	3.85	3.86	3.79
H2′	6.87	6.75	7.00	6.68	6.71; 6.77	6.59	6.80	6.61	6.95	6.56
H5′	6.74	6.73	6.78	6.74	6.74; 6.74	6.66	6.72	6.84	6.76	6.68
H6′	6.74	6.72	6.83	6.62	6.66; 6.73	6.55	6.65	6.63	6.81	6.51
H7′a	4.48	4.53	4.48	3.81	4.34; 4.64	2.56	2.50	2.51	4.71	2.53
H7′b	-	-	-	-	-	2.67	2.94	2.95	-	2.53
H8′	2.54	2.23	2.63	1.76	2.55; 2.62	1.90	2.74	2.51	3.15	2.49
H9′a	3.94	4.11	3.63	3.40	4.05; 4.17	3.56	3.72	4.01	3.84	3.92
H9′b	4.25	4.11	3.63	3.71	4.11; 4.34	3.61	3.99	4.04	4.23	4.16
OMe′	3.82	3.78	3.88	3.78	3.78; 3.80	3.74	3.83	3.86	3.86	3.78

* isolated as a mixture of diastereoisomers at C7′

Table S2. Canonical SMILES of identified compounds

Compounds	Canonical SMILES
1.	C1C(C(OC2=CC(=CC(=C21)O)O)C3=CC(=C(C(=C3)O)O)O)O
2.	C1C(C(OC2=C1C(=CC(=C2C3C(C(OC4=CC(=CC(=C34)O)O)C5=CC(=C(C(=C5)O)O)O)O)O)C6=CC(=C(C(=C6)O)O)O)O
3.	C1C(C(OC2=C1C(=CC(=C2C3C(C(OC4=CC(=CC(=C34)O)O)C5=CC(=C(C(=C5)O)O)O)O)O)C6=CC(=C(C(=C6)O)O)O)O
4.	C1C(C(OC2=C1C(=CC(=C2C3C(C(OC4=C(C(=CC(=C34)O)O)C5C(C(OC6=CC(=CC(=C56)O)O)C7=CC(=C(C(=C7)O)O)O)C8=CC(=C(C(=C8)O)O)O)O)O)O)C9=CC(=C(C(=C9)O)O)O)O
5.	C1C(C(OC2=CC(=CC(=C21)O)O)C3=CC(=C(C(=C3)O)O)O)O
6.	C1C(C(OC2=C1C(=CC(=C2C3C(C(OC4=CC(=CC(=C34)O)O)C5=CC(=C(C(=C5)O)O)O)O)O)C6=CC(=C(C(=C6)O)O)O)O
7.	C1C(C(OC2=CC(=CC(=C21)O)O)C3=CC(=C(C(=C3)O)O)O)O
8.	C1=C(C=C(C(=C1O)O)O)C2C(C(=O)C3=C(C=C(C=C3O2)O)O)O
9.	COC1=C(C=CC(=C1)C2C(C(CO2)C(C3=CC(=C(C(=C3)O)OC)O)CO)O
10.	C1=CC(=C(C=C1C=CC2=CC(=CC(=C2)OC3C(C(C(C(O3)CO)O)O)O)O)O)O
11.	C1=CC(=CC=C1C2C(C(=O)C3=C(C=C(C=C3O2)O)O)O)O
12.	COC1=C(C=CC(=C1)CC2C(COC2O)C(C3=CC(=C(C(=C3)O)OC)O)O
13.	C1=CC(=C(C=C1C2C(C(=O)C3=C(C=C(C=C3O2)OC4C(C(C(C(O4)CO)O)O)O)O)O)O)O
14.	C1=CC(=C(C=C1C=CC2=CC(=CC(=C2)OC3C(C(C(C(O3)CO)O)O)O)O)O)O
15.	C1C(C(OC2=C1C(=CC(=C2C3C(C(OC4=CC(=CC(=C34)O)O)C5=CC(=C(C(=C5)O)O)O)O)O)C6=CC(=C(C(=C6)O)O)O)O
16.	COC1=C(C=CC(=C1)C2C(C(CO2)C(C3=CC(=C(C(=C3)O)OC)O)CO)O
17.	C1=CC(=CC=C1C=CC2=CC(=CC(=C2)OC3C(C(C(C(O3)CO)O)O)O)O)O
18.	C1=CC(=C(C=C1C2C(C(=O)C3=C(C=C(C=C3O2)O)O)O)O)O
19.	COC1=C(C=CC(=C1)C=CC2=CC(=CC(=C2)OC3C(C(C(C(O3)CO)O)O)O)O)O
20.	COC1=C(C=C2C(C(C(CC2=C1)CO)CO)C3=CC(=C(C(=C3)O)OC)O
21.	COC1=C(C=C2C(C(C(CC2=C1)C(=O)O)CO)C3=CC(=C(C(=C3)O)OC)O
22.	COC1=C(C=CC(=C1)CC2COC(=O)C2C(C3=CC(=C(C(=C3)O)OC)O)O
23.	COC1=C(C=CC(=C1)CC(CO)C(CC2=CC(=C(C(=C2)O)OC)CO)O
24.	C1C(OC2=CC(=CC(=C2C1=O)O)O)C3=CC(=C(C=C3)O)O
25.	C1=C(C=C(C(=C1O)O)O)C2=C(C(=O)C3=C(C=C(C=C3O2)O)O)O
26.	COC1=C(C=CC(=C1)CC(CO)C(CC2=CC(=C(C(=C2)OC(CO)C(C3=CC(=C(C(=C3)O)OC)O)OC)CO)O
27.	COC1=C(C=CC(=C1)CC2COC(C2CO)C3=CC(=C(C(=C3)O)OC)O
28.	COC1=C(C=CC(=C1)CC2COC(C2CO)C3=CC(=C(C(=C3)OC(CO)C(C4=CC(=C(C(=C4)O)OC)O)OC)O
29.	COC1=C(C=CC(=C1)CC2COC(=O)C2(CC3=CC(=C(C(=C3)O)OC)O)O
30.	COC1=C(C=CC(=C1)C2C3COC(C3CO2)C4=CC(=C(C(=C4)O)OC)O
31.	C1=CC(=C(C=C1C2=C(C(=O)C3=C(C=C(C=C3O2)O)O)O)O)O
32.	COC1=C(C=CC(=C1)CC2COC(=O)C2CC3=CC(=C(C(=C3)O)OC)O
33.	C1=CC=C(C=C1)C2C(C(=O)C3=C(C=C(C=C3O2)O)O)O
34.	C1=CC=C(C=C1)C=CC2=CC(=CC(=C2)O)O
35.	C1C(OC2=CC(=CC(=C2C1=O)O)O)C3=CC=CC=C3
36.	CC(=O)OC1C(OC2=CC(=CC(=C2C1=O)O)O)C3=CC=CC=C3
37.	COC1=CC(=CC(=C1)C=CC2=CC=C(C=C2)O)OC
38.	COC1=CC(=CC(=C1)O)C=CC2=CC=CC=C2

39.	CC(CC(=O)C=C(C)C)C1CCC(=CC1)C(=O)OC
40.	COC1=CC(=CC(=C1)C=CC2=CC=CC=C2)OC
41.	CC(=C1CCC2C(=C1)CCC3C2(CCCC3(C)C(=O)O)C)C
42.	CC(C)C1=CC2=CCC3C(C2CC1)(CCCC3(C)C(=O)O)C
